# A global assessment of a large monocot family highlights the need for group-specific analyses of invasiveness

**DOI:** 10.1093/aobpla/plw009

**Published:** 2016-02-12

**Authors:** Desika Moodley, Şerban Procheş, John R. U. Wilson

**Affiliations:** 1 School of Environmental Sciences, University of KwaZulu-Natal, Westville Campus, Private Bag X54001, Durban 4000, South Africa; 2 Invasive Species Programme, South African National Biodiversity Institute, Kirstenbosch Research Centre, Claremont 7735, South Africa; 3 Centre for Invasion Biology, Department of Botany and Zoology, Stellenbosch University, Private Bag X1, Matieland 7602, South Africa

**Keywords:** Araceae, biological invasions, boosted regression trees, invasiveness, predictions, stages of invasion, traits

## Abstract

Significant progress has been made in understanding biological invasions recently, and one of the key findings is that the determinants of naturalization and invasion success vary from group to group. Here, we explore this variation for one of the largest plant families in the world, the Araceae. This group provides an excellent opportunity for identifying determinants of invasiveness in herbaceous plants, since it is one of the families most popular with horticulturalists, with species occupying various habitats and comprising many different life forms. We first developed a checklist of 3494 species of Araceae using online databases and literature sources. We aimed to determine whether invasiveness across the introduction–naturalization–invasion continuum is associated to particular traits within the family, and whether analyses focussed on specific life forms can reveal any mechanistic correlates. Boosted regression tree models were based on species invasion statuses as the response variables, and traits associated with human use, biological characteristics and distribution as the explanatory variables. The models indicate that biological traits such as plant life form and pollinator type are consistently strong correlates of invasiveness. Additionally, large-scale correlates such as the number of native floristic regions and number of introduced regions are also influential at particular stages in the invasion continuum. We used these traits to build a phenogram showing groups defined by the similarity of characters. We identified nine groups that have a greater tendency to invasiveness (including *Alocasia*, the Lemnoideae and *Epipremnum*). From this, we propose a list of species that are not currently invasive for which we would recommend a precautionary approach to be taken. The successful management of plant invasions will depend on understanding such context-dependent effects across taxonomic groups, and across the different stages of the invasion process.

## Abstract (precis)

There are several emerging generalizations in invasion biology, but often the factors determining invasiveness are group-specific. Similar to other plant families, Araceae species (arums or aroids) with large native ranges and that have been widely introduced are more likely to become invasive. What is unique to the family is the great diversity of growth forms, some more likely to become invasive than others. We identify nine lineages in the family that have a greater tendency to invasiveness (including the duckweed lineage, as well as the genera *Alocasia* and *Epipremnum*). A precautionary approach should be taken for such clades.

## Introduction

Trade and transport of goods by humans have connected regions across the globe (Hulme 2009; Pyšek *et al*. 2010). These pathways break down geographic barriers, which results in thousands of species being introduced outside their native ranges (Wilson *et al*. 2009; Pyšek *et al*. 2011). Of the introduced species, some are able to reproduce and form self-replacing populations to become naturalized, but only a small subset progress to become invasive (Williamson and Fitter 1996; Richardson *et al*. 2000; Lockwood *et al*. 2005; Blackburn *et al*. 2011). Identifying why some species become invasive in the introduced range while others do not is one of the most important but challenging questions in invasion ecology. By improving our understanding of the drivers linked to biological invasions, we can also develop better management practices and predict potential invasions.

The conceptualized invasion process comprises a series of barriers that a species must overcome to become naturalized and invasive in the introduced range (Richardson *et al*. 2000; Blackburn *et al*. 2011). A general understanding over the last several decades is that invasive species possess particular traits that allow them to overcome the invasion barriers in the introduced range. In the literature, species traits such as rapid growth rates and high reproductive output (Grotkopp and Rejmánek 2007; Pyšek and Richardson 2007; van Kleunen *et al*. 2010), as well as their introduction history, such as high propagule pressure and a long residence time (Pyšek *et al*. 2009*b*; Simberloff 2009), have been shown to be important determinants of invasiveness, but their relative importance varies across studies. The likelihood of invasiveness has also been predicted by attributes of the native range, such as large range sizes, and environmental similarity with the introduced range (Guisan and Thuiller 2005; Hui *et al*. 2011). In addition, different traits become important at different stages of the invasion process (Richardson and Pyšek 2012). For example, a large proportion of the alien plants have been introduced by humans over many years via the horticultural pathway, and this facilitates invasions through high propagule pressure and long residence times (Dehnen-Schmutz and Touza 2008; Lambdon *et al*. 2008; Pyšek *et al*. 2009*b*).

Although there are several hypotheses explaining traits driving invasiveness, identifying a general suite of traits has proved difficult (Richardson and Pyšek 2006; Jeschke *et al*. 2012). To date, empirical evidence shows that different sets of traits become important in different situations and the determinants of invasiveness are context dependent (e.g. Thompson *et al*. 1995; Rejmánek 1996; Prinzing *et al*. 2002; Pyšek *et al*. 2009*a*; van Kleunen *et al*. 2010; Funk 2013; Moodley *et al*. 2013). Furthermore, while some species perform better with the predicted invasive traits, it is not a feature shared by all invasive species (Alpert *et al*. 2000; Lloret *et al*. 2005; Richardson and Pyšek 2006; Tecco *et al*. 2010). One line of reasoning is that invasive species are associated with invasion syndromes. For example, invasion success may be specific to particular taxonomic groups, habitats or species life-history traits (Pyšek *et al*. 2012; Kueffer *et al*. 2013; Perkins and Nowak 2013). Therefore, instead of trying to identify general trends between invasive and non-invasive species across a wide range of taxa, it would be ideal to conduct in-depth case studies within taxonomic groups.

Araceae, also known as the arum or aroid family, is one of the oldest and the third largest monocotyledonous family in the world, after orchids and grasses (Mayo *et al*. 1997; Nauheimer *et al*. 2012). A unique feature of all species in this family is that their inflorescences consist of a spadix and a spathe (Chartier *et al*. 2014). Aroids mostly occur in the tropics where they are concentrated in Southeast Asia, tropical America and the Malay Archipelago, and they comprise diverse life forms that occupy a wide range of habitats such as aquatic, terrestrial and ephiphytic (Grayum 1990; Mayo *et al*. 1997; Cabrera *et al*. 2008). In addition, aroids have been used for decades as a food source, for medicinal purposes and in horticulture (Croat 1994; Mayo *et al*. 1997; Kubitzki 1998). Given their large diversity and distribution, as well as their long history of introduction, Araceae serves as an excellent taxonomic group for identifying determinants of invasiveness in herbaceous plants.

In this study, we focussed on introduction dynamics, characteristics of species’ native ranges and biological traits to identify correlates of invasiveness within the Araceae family. Given that there are a variety of life forms in Araceae, we hypothesized that when all species were analysed together, the only factors that would be significantly correlated to invasiveness would be factors seen to have a consistent influence across previously studied groups (e.g. native range size). However, repeating the analyses separately for different life forms would reveal specific mechanistic correlates of invasiveness. Our objectives were therefore to (i) create a species inventory using databases and literature sources; (ii) describe the invasion status of all species; (iii) identify which factors (native range characteristics, introduction dynamics and biological traits) influence introduction, naturalization and invasion success and whether this varied for different life forms; and (iv) predict which species will become invasive in future.

## Methods

### Global aroid database

Currently, there are no global databases listing all species belonging to Araceae. However, recent publications by Boyce and Croat (2011 onwards) provide the number of published and estimated species for each genus. This key resource gave us an initial idea of the aroid taxonomy. In order to create a comprehensive species inventory that includes data on accepted genera, species and synonyms, we surveyed a wide range of online databases (eMonocot, International aroid society, The Plant List, USDA Germplasm Resources Information Network and World Checklist of Selected Plant Families). Given the large number of estimated and undescribed species in this family, it is likely that there are aroid species that we did not include in our list.

### Species status

The status of introduced, naturalized and invasive species is described in a wide variety of sources (e.g. on the internet, in published and unpublished literature). Since the criteria for defining naturalized and invasive species differ across studies, it is important to use reliable sources (Falk-Petersen *et al*. 2006). We used multiple sources that contain a broad range of taxa, habitats and ecosystem types. This included (i) online databases (Atlas of living Australia, Calflora, Center for Invasive Species and Ecosystem Health, DAISIE, eMonocot, FloraBase, GBIF, GCW, GISD, HEAR, Invasive species of Japan, Randall (2007) and The PLANTS database), (ii) published literature (New Zealand naturalized plant checklist) and (iii) expert opinion (A. Haigh, N. Köster, R. Li, G. Seznec, P. Boyce, pers. comm.).

### Determinants of invasiveness

Explanatory variables related to biological traits, biogeographical factors and human usage were selected to predict invasiveness (Table 1). We used these traits and factors because they were shown to be important drivers of invasiveness in other taxonomic groups such as Australian acacias (Castro-Díez *et al*. 2011; Gibson *et al*. 2011), Cactaceae (Novoa *et al*. 2015), pines (Zenni and Simberloff 2013) and Proteaceae (Moodley *et al*. 2013). Binary response variables were categorized into three groups: non-introduced vs introduced (but not naturalized) species, introduced (but not naturalized) vs naturalized (but not invasive) species and naturalized (but not invasive) vs invasive species. These groupings describe the stages that species need to successfully transition through to become invasive (Blackburn *et al*. 2011).

**Table 1.** Summary of traits used as explanatory variables in the analyses for identifying potential drivers of invasiveness in Araceae. The number of species for which data were available is shown (out of a total of 3494 species). The range and median values for integer variables are shown in parentheses.

Trait	Levels	Number of species	Type of variable
Introduction dynamics	Food source; medicine; fibre production; horticulture; agroforestry; phytoremediation	546	Categorical
	Total number of uses	546	Integer (1–5; 1)
	Number of introduced regions (proxy for propagule pressure)	514	Integer (1–50; 1)
Native range	34 floristic native regions classified according to Good (1974)	3490	Categorical, binary
	Total number of native regions (proxy for range size)	3490	Integer (1–31; 1)
	Habitat (desert and xeric shrubland; Mediterranean forests, woodland and scrub; temperate mixed forest; tropical dry forest; tropical moist forest)	3494	Categorical
Biological traits	Pollinator type (bees; beetles; flies; combination)	3250	Categorical
	Flower sexuality (bisexual; unisexual)	3470	Categorical, binary
	Regeneration mechanism (seed; vegetative; both)	444	Categorical
	Life form chamaephyte; epiphyte; geophyte; helophyte; hemicryptophyte; hemiepiphyte; hyrdophyte; lithophyte; phanerophyte	3426	Categorical

### Statistical analyses

All analyses were performed in the R software version 2.15.1 (R Development Core Team 2012). We used boosted regression trees (BRTs) to assess the relationship of the explanatory variables with the three transition stages, first using all species belonging to Araceae, followed by models developed for particular life forms. The BRT models were fitted using the ‘gbm.step’ function from the gbm package version 1.6-3.2 (Ridgeway 2012).

Boosted regression trees are an advanced machine learning technique that applies an iterative method that sequentially builds multiple simple models, using the residuals from each subset of data during model fitting, to produce one ensemble model (Friedman 2001; Elith *et al*. 2008). This technique improves the models’ predictive performance (Elith *et al*. 2006). Among some of the advantages of this technique are that it can be fitted to a variety of response types (e.g. Gaussian, Poisson and binomial), it handles complex interactions between variables more efficiently than traditional methods (i.e. generalized linear models), it identifies important predictor variables and it addresses issues like missing data and outliers (Friedman 2002; Elith *et al*. 2008).

Elith *et al*. (2008) provide details on selecting optimal settings for model fitting. These settings include the learning rate (shrinkage parameter that determines the contribution of each tree to the growing model) and tree complexity (specifies the number of nodes on each tree which controls whether interactions are fitted) which must be adjusted to produce a model comprising at least 1000 trees. Boosted regression tree results include a measure of the comparative strength of association between the response variable and predictor variables (i.e. percentage deviance explained), and a cross-validation (CV) coefficient that indicates the degree to which the model fits withheld data.

For this study, we first built preliminary models for each stage of the invasion continuum using all the predictor variables listed in Table 1 so that we could identify those with the greatest predictive contributions and reduce the overall number of variables in our analyses. The models were built with the default 10-fold cross-validation. The relative influence of predictor variables is determined by how often a variable was selected for splitting, weighted by the improvement of the model results (Elith *et al*. 2008). From these results, we only kept predictors that contributed at least 5 % to the models. From those, we performed a correlation test using Kendall's rank correlation. As none of the predictors were strongly correlated (*r*^2^ < 0.65), all were retained in the model. The models that were developed for particular life forms were only run for the introduction stage because of small datasets.

Boosted regression tree model calibration is prone to overfitting, and there are several ways to reduce this behaviour. A key approach of the model building process is to use validation processes that require a proportion of the dataset to be withheld. Here, cross-validation was performed using 75 % of the data for training the model and the remaining 25 % for testing. We used the caret package, version 6.0-24 (Kuhn 2014), which creates random training and test sets while stratifying by the *y* variable. To evaluate model performance, we used the average percentage deviance explained and the average cross-validation area under the receiver operating characteristic (AUC) curve. Hosmer and Lemeshow (2000) state that an AUC value between 0.7 and 0.8 can be regarded as an acceptable model performance, 0.8 and 0.9 is excellent and higher than 0.9 is considered outstanding. A value of 0.5 or lower indicates that predictions are worse than random. Due to the relatively low number of invasive Araceae, we could not fit training and testing datasets for the invasion model. Therefore, we only used 10-fold CV for model development and the cross-validation AUC value for evaluation (Elith *et al*. 2008). Cross-validation provides a means for testing the model on withheld portions of data, while still using the full dataset at some stage to fit the model. The optimal parameter settings that were used in the final models are presented in Table 2.

**Table 2.** Optimal parameter settings used in calibrating the BRTs that produced the best performing introduction–naturalization–invasion models. To reduce overfitting, we used cross-validation that was performed by splitting 75 % of the data for training the model and 25 % for testing. We tested various learning rates (0.1–0.0005), bag fractions (0.1–0.8) and levels of tree complexity (1–5). By trial and error, we determined the most effective algorithm parameters for our dataset, which is depicted below.

	Introduction model	Naturalization model	Invasion model
Sample size (*n*)			
Full dataset	3494	514	46
Training data	2621	386	–
Test data	873	128	–
Parameters			
Learning rate	0.001	0.001	0.001
Tree complexity	3	3	3
Bag fraction	0.5	0.5	0.75

Lastly, using predictors that met the BRT criteria (i.e. predictors that contributed at least 5 % to the model), we either built generalized linear models with binomial errors or used independent *t*-tests. This step provided insight into the individual explanation potential of each variable.

### Predicting potentially invasive species

Using published literature, the first step was to examine the family tree and only select monophyletic groups. This selection controlled for phylogenetic effects as best as possible. Given that very few genera have published species level phylogenies, and most genera contain only non-introduced species, we decided to only include genera with known invasive species records. However, most of the invasive genera also lacked complete species level phylogenies. Selecting groups with invasive genera was important as it allowed inferring potentially invasive species in a more insightful manner (i.e. the selected groups comprised traits that are already known to confer invasiveness). In addition, it is assumed that species that have the potential to become invasive will be ones that (i) have relatives that are invasive and (ii) have similar traits as invasive species. These two assumptions were used to formulate criteria to shortlist genera that have a known history of invasiveness, and should be scrutinized further.

Second, using the results from the BRT analyses, we scored species on traits that have already been shown to facilitate naturalization and invasion success in Araceae. Following species scoring, we removed uninformative character states from the matrix. Finally, we constructed the phenograms using Jaccard's index and the unweighted pair group method with arithmetic mean (UPGMA) implemented in the FreeTree software version 0.9.1.50 (Pavlicek *et al*. 1999), which ranked species based on their overall similarity of characters. The phenograms clustered species based on the statistical similarity of their traits and also reflect evolutionary relatedness since only monophyletic groups were selected (see above). This allowed us to match species clusters with their associated invasion status. We used this approach as a tool to predict species that are not yet invasive but likely pose a relatively high invasion risk.

## Results

### Global aroid list

**Figure 1.**
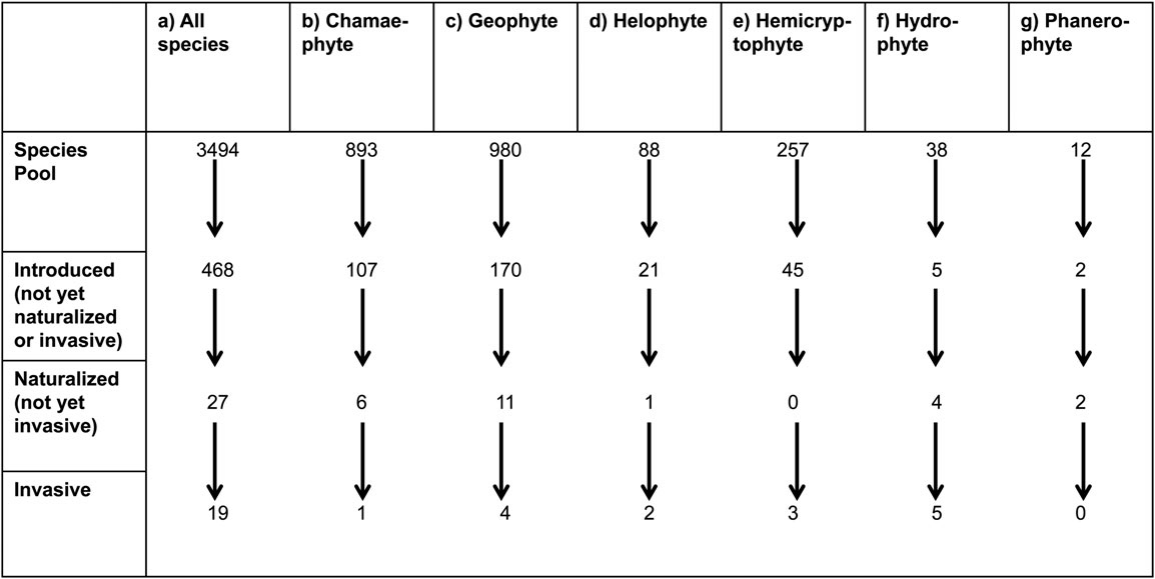
Numbers of Araceae species at different stages along the introduction–naturalization–invasion continuum. The selected plant life forms that are depicted here tend to be introduced more often.

The Araceae database comprises 115 genera with 3494 species worldwide, predominantly tropical in their distribution **[see Supporting Information—Table S1]**. Relatively few species (13 %) have been introduced (not yet naturalized or invasive) outside their native ranges, with 27 species classified as naturalized (not yet invasive) and 19 as invasive (Fig. 1A). Chamaephytes (Fig. 1B) and geophytes (Fig. 1C) contain the largest numbers of species, as well as large proportions of introduced (not naturalized or invasive) species (12 and 17 %, respectively), but they have low numbers of invasive species. Helophytes have the greatest proportion of introduced (not naturalized or invasive) species (24 %) and also a relatively high proportion of naturalized (not invasive) and invasive species (Fig. 1D). 18 % of hemicryptophyte species had been introduced (not naturalized or invasive), but none were naturalized (not invasive) and only 1 % was invasive (Fig. 1E). Hydrophytes seem to be the most successful. 13 % of hydrophyte species have been introduced (not naturalized or invasive), 11 % naturalized (not invasive), and 13 % invasive (Fig. 1F). In contrast, phanerophytes have a large proportion of introduced (not naturalized or invasive) species and naturalized (not invasive) species (17 %) but no invasive species (Fig. 1G).

### Model performance

The predictive performance for the models varied from acceptable (for the introduction and invasion model) to outstanding (for the naturalization model). The final BRT introduction model explained 13 % of the mean total deviance (1 − mean residual deviance/mean total deviance). The test data AUC score was 0.72, and the full dataset cross-validation coefficient AUC score was 0.70 ± 0.011 (mean ± standard error). The naturalization model accounted for 59 % of the total deviance, and the test data AUC score was 0.98, while the cross-validation coefficient AUC score was 0.93 ± 0.021. The invasion model accounted for 36 % of the total deviance, and the cross-validation coefficient AUC score was 0.74 ± 0.072.

### Factors associated with species’ native range, introduction dynamics and biological traits in explaining introduction, naturalization and invasion success

**Figure 2.**
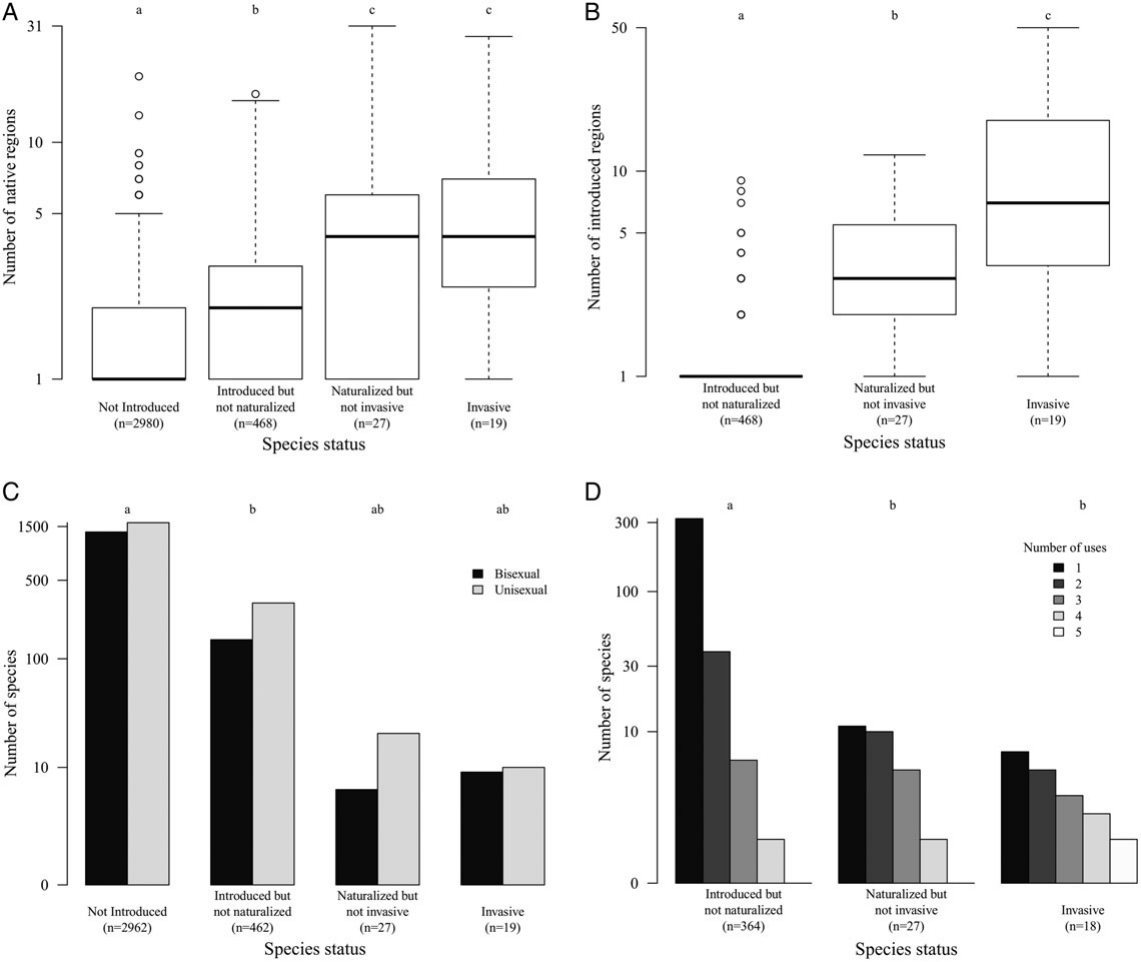

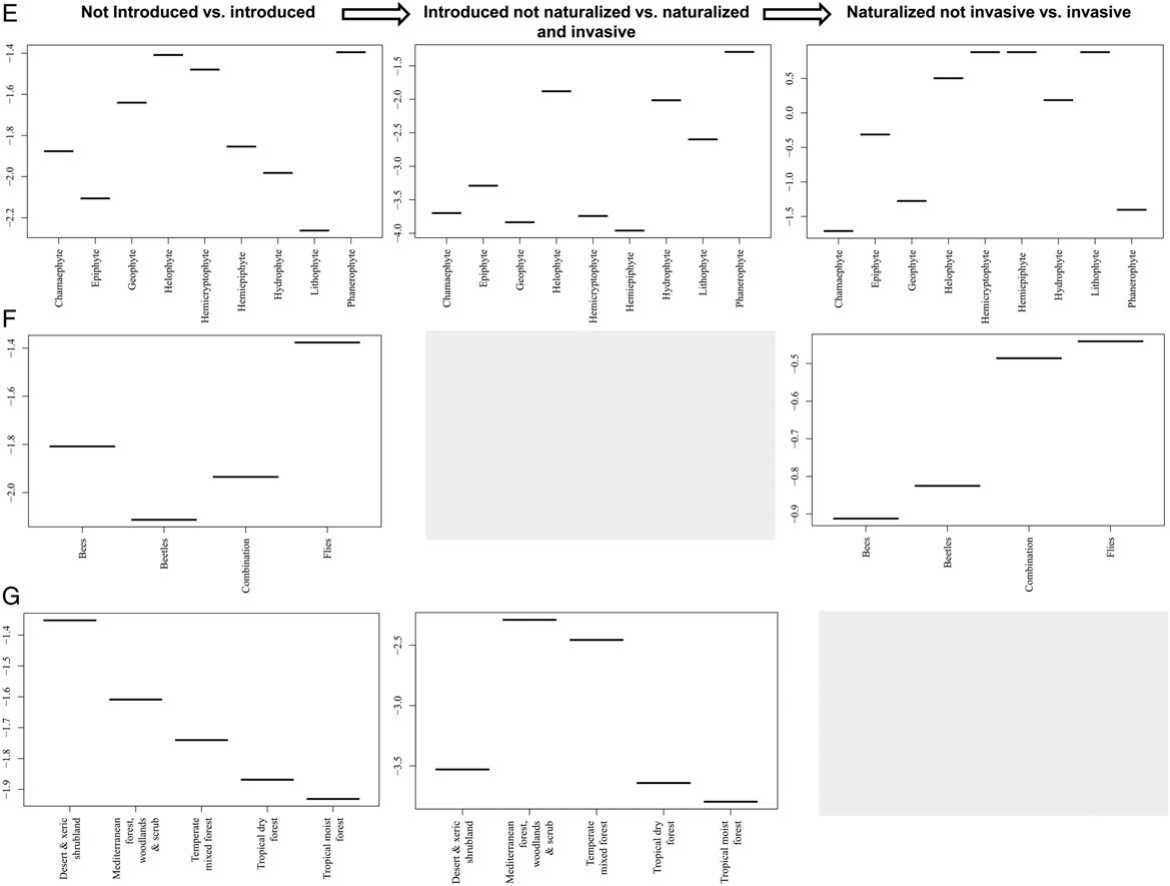
The relationship between the introduction status of Araceae species and the parameters found to have a significant effect using BRTs. (A) Invasive taxa have larger native range sizes. Native range size is measured here in terms of the number of floristic regions based on Good's (1974) classification. Araceae naturally occur in 34 of the 37 floristic regions. (B) Invasive species tend to have been introduced to more regions than naturalized species, and almost 90 % of species that have been introduced to only one region have not yet naturalized. (C) Species with unisexual flowers tend to have overcome more of the barriers to invasion than species with bisexual flowers. (D) Species with a broad range of uses have naturalized and become invasive more often. Six different categories of human usage were considered: food source, medicine, fibre production, horticulture, agroforestry and phytoremediation. (E) Different life forms varied in their importance at different stages of the invasion. (F) Species that were fly pollinated or had a combination of pollinator types were introduced and became invasive relatively more frequently than bee- or beetle-pollinated species. (G) Species native to Mediterranean and temperate mixed forests tend to naturalize more often. There were few data on the human uses of species that had not been introduced outside their native range, and so this category was excluded. In (A and B), the box is the interquartile range, and the bold centre line is the median. Different letters denote different values using Tukey's multiple comparisons of means test. In (E–G), tests were done using the original data, though the panels actually show plots of the fitted functions produced by BRTs, which indicate the effect on species presence/absence across the INI stages (*y*-axes) by each predictor variable (*x*-axes). For the relative contribution of each variable to the total deviance explained, see Table 3. Grey panels indicate factors with low importance in the INI continuum, and therefore excluded from the model.

The number of native floristic regions, which we used as a proxy for range size, was an important predictor for introduction [Table 3; **Supporting Information—Fig. S1**]. Species that occur over more floristic regions in their native range tend to be introduced more often (Fig. 2A; *F*_3,3490_ = 46.7, *P* < 0.001).

**Table 3.** Variables shown in the BRT analyses to have the greatest influence on the prediction of introduction, naturalization and invasion. The percentage contribution of a variable is based on the number of times the variable is selected for splitting, weighted by the squared improvement to the model as a result of each split and averaged over all trees. For each model, the contribution of the variables is scaled to add up to 100 %, with higher numbers indicating stronger influence on the response.

Model	Variable	Percentage contribution
Introduction	Number of native regions	30.00
	Life form	26.00
	Pollinator type	17.70
	Species native to Polynesia	9.90
	Flower sexuality	8.20
	Habitat	8.20
Naturalization	Number of introduced regions	65.90
	Life form	16.00
	Habitat	9.80
	Number of uses	8.30
Invasion	Life form	48.90
	Number of introduced regions	35.30
	Pollinator type	15.90

The number of introduced regions, which we used as a proxy for propagule pressure, was an important predictor of naturalization and invasion (Table 3). This suggests that species that are introduced to more regions in their new range tend to overcome the naturalization and invasion barriers (Fig. 2B; *F*_2,511_ = 266, *P* < 0.001).

Flower sexuality was significant for species overcoming the introduction barrier (Table 3). Relative to non-introduced species, there are significantly more unisexual flowers among introduced species, but there are no significant differences across the naturalization and invasion stages (Fig. 2C; *F*_3,3466_ = 11.29, *P* < 0.001). Tropical climbers largely comprise species with unisexual flowers, which explains why species with this flower type are likely to be introduced.

Data on the purpose of introduction were limited, as only 12 % of the species had information on human usage (*n* = 409). Nevertheless, we found number of uses to be an important predictor of naturalization (Table 3). Introduced species that had failed to naturalize tended to have fewer uses than naturalized and invasive species (Fig. 2D; *F*_2,406_ = 53.55, *P* < 0.001).

In comparison with other plant life forms, chamaephytes (*z* = −19.165; *P* < 0.001), geophytes (*z* = 3.587; *P* < 0.001), helophytes (*z* = 3.626; *P* < 0.001), hemicryptophytes (*z* = 2.386; *P* = 0.0170), hydrophytes (*z* = 3.940; *P* < 0.001) and phanerophytes (*z* = 1.980; *P* = 0.0477) have been introduced more frequently outside their native ranges. After introduction, hydrophytes (*z* = 4.870; *P* < 0.001) are the most successful in overcoming the combination of the naturalization and invasion barriers (Fig. 2E). These successful species are mainly used as ornamentals (including plants used in gardens, landscaping, cut flowers, aquariums and ponds) or as a food source. This demonstrates that horticulture provides a major pathway for plant invasions in Araceae. Even though life form was the most important factor across all stages (Table 3), we did not find a significant difference between the different life forms for the invasion stage. This can be attributed to the large number of naturalized species across the range of life forms that were able to become invasive.

The method of pollination was an important correlate for species introduction and invasion (Table 3). Species pollinated by bees (*z* = −7.930; *P* < 0.001) and flies (*z* = 3.149; *P* = 0.00164) were introduced more often. Although not significant, the combination of pollinators (*z* = 0.007; *P* > 0.05) and fly-pollinated (*z* = 0.007; *P* > 0.05) species are more invasive (Fig. 2F). Pollination by flies is typical of plants in the Araceae family. Fly-pollinated species being able to overcome the introduction barrier is probably an artefact of human use, since these species comprise popular ornamental plants that are used for their unique inflorescences (e.g. *Amorphophallus*, *Anthurium*, *Arisaema* and *Zantedeschia*), decorative foliage (e.g. *Philodendron* and *Schismatoglottis*) or as aquarium plants (e.g. *Cryptocoryne*). Nevertheless, these pollinators highlight a specialized pollination syndrome in Araceae.

The type of habitat a species occupies in its native range was an important correlate of introduction and naturalization (Table 3). Although most of the species originating in desert and xeric shrublands are introduced (*z* = −2.587; *P* = 0.00969), they have not yet been recorded to naturalize or invade (Fig. 2G). Species native to humid regions, Mediterranean forests (*z* = −3.569; *P* = 0.00289) and temperate mixed forests (*z* = −3.922; *P* < 0.001), in particular, tend to overcome the introduction and naturalization barriers.

From the 34 floristic regions that Araceae occupy, species native to the Polynesian province were introduced more often (Table 3). While larger floristic regions such as Malaysia and Euro-Siberia were more important in terms of the total number of invasive species originating there, Polynesia had the largest proportion of introduced species (64 %), with 24 % classified as naturalized and 12 % as invasive.

Lastly, after incorporating particular life forms into the analyses, we did not find specific correlates of invasiveness that differed from the original model; therefore, we rejected the second hypothesis. We found the number of introduced regions and reproductive characteristics to be important for chamaephytes; the number of native floristic regions, pollinator type, species native to West African rainforests and human use were important for epiphytes; and the number of introduced regions and reproductive characteristics were important for geophytes. In addition, we did not find a strong clustering in life forms across the family **[see Supporting Information—Fig. S2]**.

### Predicting potentially invasive species

From the BRT models, we identified eight characteristics that facilitate species to overcome the introduction-naturalization-invasion (INI) barriers **[see Supporting Information—Table S2]**. Of the 15 invasive genera in Araceae, we constructed phenograms inclusive of 14 genera. The arguments used to identify potentially invasive species from the phenogram were based on (i) overall similarity in the character states of species, (ii) whether species group with naturalized or invasive species and (iii) whether species cluster with naturalized or invasive sister groups. From the nine monophyletic groups, species with a high risk of becoming invasive are listed in Table 4, and their respective phenograms are illustrated in **Supporting Information—Fig. S3**.

**Table 4.** A list of potentially invasive Araceae species constructed from model-based statistical inferences (i.e. UPGMA phenograms). These species are placed into groupings that are based on evolutionary relatedness (i.e. monophyletic groups) and similar ecological traits. Phenograms are illustrated in **Supporting Information—Fig. S3**.

Monophyletic group	No. of species evaluated	No. of potentially high-risk species	Potentially invasive species list **[see** **Supporting Information—Fig. S3****]**	Comments
*Alocasia*	77	5	*Alocasia longiloba*, *Alocasia odora*, *Alocasia acuminata*, *Alocasia brisbanensis*, *Alocasia hypnosa*	High likelihood for the listed non-introduced and introduced species to become invasive
*Amydrium*, *Anadendrum*, *Epipremnum*, *Monstera*, *Rhaphidophora*, *Scindapsus*	82	38	*Monstera adansonii* var. *adansonii*, *Monstera deliciosa*, *Anadendrum microstachyum*, *Anadendrum latifolium*	Most species in this group are not yet introduced; however, since this group already contains two invasive species, all species that are not listed requires further evaluation
*Ariopsis*, *Colocasia*, *Remusatia*, *Steudnera*	20	11	*Remusatia hookeriana*, *Remusatia pumila*, *Colocasia affinis*	One cluster contains the invasive *Colocasia esculenta*; therefore, species in this group requires more attention
*Arophyton*, *Carlephyton*, *Colletogyne*, *Peltandra*, *Typhonodorum*	2	1	*Peltandra sagittifolia*	*Peltandra virginica* is invasive and sister species. *Peltandra sagittifolia* has been introduced outside its native range
*Arum*, *Biarum*, *Dracunculus*, *Eminium*, *Helicodiceros*, *Sauromatum*, *Theriophonum*, *Typhonium*	55	23	*Arum maculatum*, *Dracunculus vulgaris*, *Typhonium blumei*, *Typhonium roxburghii*, *Sauromatum venosum*, *Sauromatum horsfieldii*, *Typhonium trilobatum*	Many species require further evaluation. Risk assessments must be conducted prior to species introduction
*Caladium*, *Chlorospatha*, *Filarum*, *Hapaline*, *Jasarum*, *Scaphispatha*, *Syngonium*, *Ulearum*, *Xanthosoma*, *Zomicarpa* and *Zomicarpella*	169	∼107	See clusters marked with asterisks in **Supporting Information—Fig. S3F**	Large group with five naturalized, but not invasive, species and three invasive species scattered in the phenogram. All groups containing high-risk species need to be evaluated further
*Cryptocoryne*, *Lagenandra*	86	65	All species that clusters with invasive species	Phenogram shows very little structure (i.e. many species nested within groups) because fewer informative traits were used. Nevertheless, a single cluster contains the naturalized and invasive species. Therefore, all species within this group pose an invasion risk
*Gymnostachys*, *Lysichiton*, *Orontium*, *Symplocarpus*	8	6	*Lysichiton camtschatcensis*, *Symplocarpus egorovii*, *Symplocarpus foetidus*, *Symplocarpus nabekuraensis*, *Symplocarpus nipponicus*, *Symplocarpus renifolius*	High likelihood for non-introduced and introduced species to become invasive
*Lemna*, *Spirodela*, *Wolffia* and *Wolffiella*	31	8	*Lemna aequinoctialis*, *Lemna minor*, *Lemna perpusilla*, *Spirodela oligorrhiza*, *Wolffia arrhiza*, *Wolffia brasiliensis*, *Wolffiella lingulata*, *Wolffiella welwitschii*	Many invasive species in this group. The listed non-invasive species have a high invasion risk because they cluster with the invasive species

## Discussion

Identifying characteristics of successful invaders has been a major goal in invasion biology (Rejmánek 1996; Rejmánek and Richardson 1996; Rejmánek *et al*. 2005; Pyšek and Richardson 2007; Richardson *et al*. 2011; Richardson and Pyšek 2012). Our results support the understanding that although some invasive traits are shared between invasive species, this is not consistent among all taxa and they are context specific (Alpert *et al*. 2000; Richardson and Pyšek 2006; Theoharides and Dukes 2007; Moodley *et al*. 2013; Potgieter *et al*. 2014; Novoa *et al*. 2015). Our main observations were that species that have large native floristic ranges are more likely to be introduced, and introduced species that are introduced to more regions are more likely to naturalize and invade; life form is consistently a major predictor; and pollinator type might also be an important correlate and this is arguably specific to Araceae. Additionally, we found that particular traits or a combination of traits become important at different stages of the invasion continuum.

The importance of native range size (measured here in terms of the number of floristic regions) is consistent with other studies (Rejmánek 1996; Pyšek *et al*. 2009*a*; Hui *et al*. 2011; Procheş *et al*. 2012; Moodley *et al*. 2013), which also show that species with larger native ranges are more likely to be introduced and become naturalized. A large native distribution is often correlated with invasiveness because there is a higher probability that wide-ranging species will be picked up and intentionally or accidentally introduced (Blackburn and Jeschke 2009). It is also reflective of species being tolerant to a wide range of environmental conditions that pre-adapt them to survive and become established in the new region (Goodwin *et al*. 1999; Pyšek *et al*. 2009*a*).

High introduction efforts across novel ranges translate to a high propagule pressure. This finding is also in agreement with other studies (Colautti *et al*. 2006; Pauchard and Shea 2006; Moodley *et al*. 2013; Zenni and Simberloff 2013), where higher propagule pressure facilitates naturalization and invasion. This concept is based on the principle that species that are introduced across a wide area of the new region have a better chance of landing in localities that are suitable for establishment (Lockwood *et al*. 2005).

A large proportion of plant invasions result from horticultural introductions (Reichard and White 2001; Dehnen-Schmutz *et al*. 2007*b*; Keller *et al*. 2011). Araceae are often used in horticulture, with hundreds of species and cultivars. Araceae that are used by humans for more purposes have a higher probability of being introduced and becoming naturalized. In addition, the invasion stage included species with the most number of uses. Other studies also found that species used by humans have a greater chance of becoming established in the introduced region because of a higher probability of being transported and higher propagule pressure (Pyšek *et al*. 2003; Thuiller *et al*. 2006; van Kleunen *et al*. 2007).

Plant life form is a common predictor of invasiveness for Araceae species since it is shared across the INI stages. This includes species that are (i) classified as hydrophytes and (ii) used for ornamental purposes. Araceae species that conform to these categories often reproduce vegetatively, and this regeneration strategy is frequently linked to invasiveness (Kolar and Lodge 2001). Although vegetative reproduction is not associated with long-distance spread, it can play an important role in the establishment of invasive species under suitable conditions in their new range (Daehler 1998; Lloret *et al*. 2005). Given that hydrophytes are more likely to overcome the introduction and naturalization barriers, species belonging to this life form pose a greater invasion risk. Furthermore, once species overcome the introduction and naturalization barriers, species of any life form have the potential to become invasive.

Ornamental species topped the list of invasive Araceae. It is well known that species deliberately introduced for ornamental purposes are associated with successful invasions because high market availability allows for high propagule pressure (Dehnen-Schmutz *et al*. 2007*a*; Dehnen-Schmutz 2011). Species comprising invasive life forms with a potential for ornamental use should be carefully evaluated prior to introduction, and management plans specific for these plants should be put in place. In addition, any species that is likely to be introduced with high propagule pressure poses a high risk, and therefore, efforts to reduce propagule pressure may successfully prevent a proportion of invasions.

The main centres of origin and diversity of aroids are tropical regions such as tropical Asia and tropical America (Croat 1998). However, we found that species native to the Polynesian province were more successful in overcoming the introduction barriers. Forests in these Paleotropical regions are classified as one of the most wide-ranging and species-rich terrestrial habitats in the world (Whitmore 1984) across taxa, and the Araceae are no exception. A higher introduction effort of wide-ranging species could be attributable to a higher abundance and tolerance to diverse conditions in any new area and so a relatively higher ease of cultivation (Forcella and Wood 1984; Goodwin *et al*. 1999; Prinzing *et al*. 2002; Dehnen-Schmutz *et al*. 2007*b*). In addition, since Polynesia is made up of islands, introduction effort from these islands is a key driver for Araceae dispersal. It is also possible that some of these ‘native’ Polynesian species were introduced by humans (and so pre-selected for an ability to be introduced), though this remains to be determined.

Some model groups demonstrate strong mechanistic correlation to invasion, such as *Phytophthora* susceptibility in Proteaceae (Moodley *et al*. 2013) and vegetative dispersal in Cactaceae (Novoa *et al*. 2015). In Araceae, we found that most correlates are universal. However, specialized pollinator types (e.g. flies and beetles) were important for introduction and invasion, and this factor might be specific to Araceae. Most Araceae species are dependent on specialized pollinators (*n* = 900 beetles, *n* = 653 flies), and this may be limiting species that cannot spread vegetatively from becoming invasive. Species that require specialized pollination can encounter barriers to invasion when there is a lack of suitable pollinators or pollinator functional groups in their new range (Geerts and Pauw 2012). The prevention and management of potentially high-risk species is required to help reduce the threats posed by invasive alien species. On one hand, there should be management plans put in place for species that are already introduced or species with a few naturalizing populations, but which pose an invasion risk (e.g. prohibit further dissemination of potentially invasive species, remove high-risk species or issue permits for the possession of high-risk species, and consider attempting eradication or containment). On the other hand, prevention is the best line of defence and can be applied to species that are not yet introduced but have similar traits to naturalized and invasive species. For instance, groups that so far lack invasive species may contain potentially invasive species, which have not been given an opportunity to invade. Therefore, phenograms should also be used for non-invasive groups that comprise species with the same suite of characteristics as the invasive groups. Screening high-risk species using a simple method based on evolutionary history and trait similarity is a conceptual step forward that provides a general framework in trying to predictive invasiveness; however, this has ample room for improvement. In practice, this will contribute towards the battle against invasive species, since risk assessment has its greatest impact when integrated into early invasive alien species management planning (Hulme 2012; Wilson *et al*. 2013).

## Conclusions

Araceae conforms to some, but not all, of the emerging generalizations in invasion biology. In line with many other studies, Araceae species that have been widely introduced (i.e. high propagule pressure) and that have large native range sizes are more likely to be invasive. However, unlike many other groups, there was little evidence of a link between invasiveness and regeneration mechanism (i.e. by seed, vegetative or both). Instead, there was a significant effect of plant life form and pollinator syndrome. Moreover, the importance of factors varied across the INI continuum.

Since the mechanisms associated with invasiveness differ between taxa and across the INI continuum, group and stage-specific analyses are required. As more complete phylogenies and better knowledge of traits become available, these analyses are likely to become increasingly sophisticated and able to produce valuable insights into risk assessments.

## Sources of Funding

This work was supported by the South African National Department of Environment Affairs through its funding of the South African National Biodiversity Institute Invasive Species Programme.

## Contributions by the Authors

D.M. provided the theoretical framework, data collection, data analyses and write-up. Ş.P. and J.R.U.W. provided ideas and helped with the write-up.

## Conflict of Interest Statement

None declared.

## Supporting Information

The following additional information is available in the online version of this article –

**Figure S1.** Fitted function plots produced from the boosted regression tree models for species categorized in the (A) introduction, (B) naturalization and (C) invasion stages.

**Figure S2.** Stick phylogeny of Araceae lineages. Black squares correspond to each clade and their associated life form(s). For further details on the phylogeny, see Cusimano *et al*. (2011). The tree reveals that life forms are spread across the phylogeny.

**Figure S3.** Phenograms illustrating species that have a potential to become invasive based on shared traits within the following monophyletic groups: (A) Lemnoideae, (B) *Alocasia* (C) *Amydrium*, (D) *Ariopsis*, (E) *Arum*, (F) *Caladium*, (G) *Cryptocoryne* and (H) *Gymnostachys*.

**Table S1.** A comprehensive species checklist developed for Araceae in 2013–14.

**Table S2.** Eight characteristics used to construct phenograms for invasive genera.

Additional Information
