# Supplementary material for: A global assessment of a large monocot family highlights the need for group-specific analyses of invasiveness
Source: AoB Plants. 2016 Feb 12;8:plw009. doi: 10.1093/aobpla/plw009 (PMC4804228; doi:10.1093/aobpla/plw009)
Supplement: Additional Information [file supp_8_plw009_index.html]

A global assessment of a large monocot family highlights the need for group-specific analyses of invasiveness — A global assessment of a large monocot family highlights the need for group-specific analyses of invasiveness — Additional Information 

# A global assessment of a large monocot family highlights the need for group-specific analyses of invasiveness

## Additional Information

Additional Information

- Supplementary File 1 - docx file
- Supplementary File 2 - docx file
- Supplementary File 3 - docx file
- Supplementary File 4 - tif file
- Supplementary File 5 - docx file
