# Supplementary material for: A global assessment of a large monocot family highlights the need for group-specific analyses of invasiveness
Source: AoB Plants. 2016 Feb 12;8:plw009. doi: 10.1093/aobpla/plw009 (PMC4804228; doi:10.1093/aobpla/plw009)
Supplement: Additional Information [file supp_plw009_plw009supp_file1.docx]

| **Scientific name** |
| --- |
| Aglaodorum griffithii |
| Aglaonema brevispathum |
| Aglaonema chermsiriwattanae |
| Aglaonema cochinchinense |
| Aglaonema commutatum var. commutatum |
| Aglaonema commutatum var. elegans |
| Aglaonema commutatum var. maculatum |
| Aglaonema commutatum var. warburgii |
| Aglaonema cordifolium |
| Aglaonema costatum |
| Aglaonema crispum |
| Aglaonema densinervium |
| Aglaonema flemingianum |
| Aglaonema hookerianum |
| Aglaonema marantifolium |
| Aglaonema modestum |
| Aglaonema nebulosum |
| Aglaonema nitidum |
| Aglaonema ovatum |
| Aglaonema philippinense var. philippinense |
| Aglaonema philippinense var. stenophyllum |
| Aglaonema pictum |
| Aglaonema pumilum |
| Aglaonema rotundum |
| Aglaonema simplex |
| Aglaonema tricolor |
| Aglaonema vittatum |
| Alloschemone inopinata |
| Alloschemone occidentalis |
| Alocasia acuminata |
| Alocasia aequiloba |
| Alocasia alba |
| Alocasia arifolia |
| Alocasia atropurpurea |
| Alocasia augustiana |
| Alocasia baginda |
| Alocasia balgooyi |
| Alocasia beccarii |
| Alocasia boa |
| Alocasia boyceana |
| Alocasia brancifolia |
| Alocasia brisbanensis |
| Alocasia cadieri |
| Alocasia celebica |
| Alocasia chaii |
| Alocasia clypeolata |
| Alocasia cucullata |
| Alocasia culionensis |
| Alocasia cuprea |
| Alocasia decipiens |
| Alocasia decumbens |
| Alocasia devansayana |
| Alocasia fallax |
| Alocasia flabellifera |
| Alocasia flemingiana |
| Alocasia fornicata |
| Alocasia gageana |
| Alocasia grata |
| Alocasia hainanica |
| Alocasia heterophylla |
| Alocasia hollrungii |
| Alocasia hypnosa |
| Alocasia hypoleuca |
| Alocasia infernalis |
| Alocasia inornata |
| Alocasia jiewhoei |
| Alocasia kerinciensis |
| Alocasia lancifolia |
| Alocasia lauterbachiana |
| Alocasia lecomtei |
| Alocasia longiloba |
| Alocasia macrorrhizos |
| Alocasia maquilingensis |
| Alocasia megawatiae |
| Alocasia melo |
| Alocasia micholitziana |
| Alocasia miniuscula |
| Alocasia monticola |
| Alocasia navicularis |
| Alocasia nebula |
| Alocasia nicolsonii |
| Alocasia nycteris |
| Alocasia odora |
| Alocasia pangeran |
| Alocasia peltata |
| Alocasia perakensis |
| Alocasia portei |
| Alocasia princeps |
| Alocasia principiculus |
| Alocasia puber |
| Alocasia puteri |
| Alocasia pyrospatha |
| Alocasia ramosii |
| Alocasia reginae |
| Alocasia reginula |
| Alocasia reversa |
| Alocasia ridleyi |
| Alocasia robusta |
| Alocasia sanderi |
| Alocasia sarawakensis |
| Alocasia scabriuscula |
| Alocasia scalprum |
| Alocasia simonsiana |
| Alocasia sinuata |
| Alocasia suhirmaniana |
| Alocasia venusta |
| Alocasia wentii |
| Alocasia wongii |
| Alocasia zebrina |
| Ambrosina bassii |
| Amorphophallus aberrans |
| Amorphophallus abyssinicus subsp. abyssinicus |
| Amorphophallus abyssinicus subsp. akeassii |
| Amorphophallus abyssinicus subsp. unyikae |
| Amorphophallus albispathus |
| Amorphophallus albus |
| Amorphophallus amygdaloides |
| Amorphophallus andranogidroensis |
| Amorphophallus angolensis subsp. angolensis |
| Amorphophallus angolensis subsp. maculatus |
| Amorphophallus angulatus |
| Amorphophallus angustispathus |
| Amorphophallus ankarana |
| Amorphophallus annulifer |
| Amorphophallus antsingyensis |
| Amorphophallus aphyllus |
| Amorphophallus asper |
| Amorphophallus asterostigmatus |
| Amorphophallus atrorubens |
| Amorphophallus atroviridis |
| Amorphophallus bangkokensis |
| Amorphophallus barthlottii |
| Amorphophallus baumannii |
| Amorphophallus beccarii |
| Amorphophallus bequaertii |
| Amorphophallus bonaccordensis |
| Amorphophallus borneensis |
| Amorphophallus boyceanus |
| Amorphophallus brachyphyllus |
| Amorphophallus brevispathus |
| Amorphophallus bufo |
| Amorphophallus bulbifer |
| Amorphophallus calabaricus subsp. calabaricus |
| Amorphophallus calabaricus subsp. mayoi |
| Amorphophallus canaliculatus |
| Amorphophallus carneus |
| Amorphophallus chlorospathus |
| Amorphophallus cicatricifer |
| Amorphophallus cirrifer |
| Amorphophallus coaetaneus |
| Amorphophallus commutatus |
| Amorphophallus consimilis |
| Amorphophallus corrugatus |
| Amorphophallus costatus |
| Amorphophallus coudercii |
| Amorphophallus croatii |
| Amorphophallus curvistylis |
| Amorphophallus dactylifer |
| Amorphophallus declinatus |
| Amorphophallus decus-silvae |
| Amorphophallus discophorus |
| Amorphophallus dracontioides |
| Amorphophallus dunnii |
| Amorphophallus dzui |
| Amorphophallus eburneus |
| Amorphophallus echinatus |
| Amorphophallus eichleri |
| Amorphophallus elatus |
| Amorphophallus elegans |
| Amorphophallus elliottii |
| Amorphophallus erythrororrhachis |
| Amorphophallus excentricus |
| Amorphophallus flotoi |
| Amorphophallus forbesii |
| Amorphophallus fuscus |
| Amorphophallus galbra |
| Amorphophallus gallaensis |
| Amorphophallus gallowayi |
| Amorphophallus gigas |
| Amorphophallus glaucophyllus |
| Amorphophallus gliruroides |
| Amorphophallus glossophyllus |
| Amorphophallus goetzei |
| Amorphophallus gomboczianus |
| Amorphophallus gracilior |
| Amorphophallus gracilis |
| Amorphophallus haematospadix |
| Amorphophallus harmandii |
| Amorphophallus hayi |
| Amorphophallus henryi |
| Amorphophallus hetterscheidii |
| Amorphophallus hewittii |
| Amorphophallus hildebrandtii |
| Amorphophallus hirsutus |
| Amorphophallus hirtus |
| Amorphophallus hohenackeri |
| Amorphophallus hottae |
| Amorphophallus impressus |
| Amorphophallus incurvatus |
| Amorphophallus infundibuliformis |
| Amorphophallus interruptus |
| Amorphophallus johnsonii |
| Amorphophallus josefbogneri |
| Amorphophallus julaihii |
| Amorphophallus juliae |
| Amorphophallus kachinensis |
| Amorphophallus kiusianus |
| Amorphophallus konjac |
| Amorphophallus konkanensis |
| Amorphophallus koratensis |
| Amorphophallus krausei |
| Amorphophallus lacourii |
| Amorphophallus lambii |
| Amorphophallus lanuginosus |
| Amorphophallus laoticus |
| Amorphophallus lewallei |
| Amorphophallus linearis |
| Amorphophallus linguiformis |
| Amorphophallus longicomus |
| Amorphophallus longiconnectivus |
| Amorphophallus longispathaceus |
| Amorphophallus longistylus |
| Amorphophallus longituberosus |
| Amorphophallus lunatus |
| Amorphophallus luzoniensis |
| Amorphophallus lyratus |
| Amorphophallus macrorhizus |
| Amorphophallus mangelsdorffii |
| Amorphophallus manta |
| Amorphophallus margaritifer |
| Amorphophallus margretae |
| Amorphophallus maximus subsp. maximus |
| Amorphophallus maximus subsp. fischeri |
| Amorphophallus maxwellii |
| Amorphophallus mekongensis |
| Amorphophallus merrillii |
| Amorphophallus mildbraedii |
| Amorphophallus minor |
| Amorphophallus mossambicensis |
| Amorphophallus muelleri |
| Amorphophallus mullendersii |
| Amorphophallus myosuroides |
| Amorphophallus mysorensis |
| Amorphophallus napalensis |
| Amorphophallus napiger |
| Amorphophallus natolii |
| Amorphophallus niahensis |
| Amorphophallus nicolsonianus |
| Amorphophallus obovoideus |
| Amorphophallus obscurus |
| Amorphophallus ochroleucus |
| Amorphophallus ongsakulii |
| Amorphophallus operculatus |
| Amorphophallus opertus |
| Amorphophallus paeoniifolius |
| Amorphophallus palawanensis |
| Amorphophallus paucisectus |
| Amorphophallus pendulus |
| Amorphophallus perakensis |
| Amorphophallus pilosus |
| Amorphophallus plicatus |
| Amorphophallus polyanthus |
| Amorphophallus prainii |
| Amorphophallus preussii |
| Amorphophallus prolificus |
| Amorphophallus purpurascens |
| Amorphophallus pusillus |
| Amorphophallus putii |
| Amorphophallus pygmaeus |
| Amorphophallus ranchanensis |
| Amorphophallus reflexus |
| Amorphophallus rhizomatosus |
| Amorphophallus richardsiae |
| Amorphophallus rostratus |
| Amorphophallus rugosus |
| Amorphophallus sagittarius |
| Amorphophallus salmoneus |
| Amorphophallus saraburensis |
| Amorphophallus saururus |
| Amorphophallus scaber |
| Amorphophallus schmidtiae |
| Amorphophallus scutatus |
| Amorphophallus serrulatus |
| Amorphophallus sinuatus |
| Amorphophallus sizemoreae |
| Amorphophallus smithsonianus |
| Amorphophallus sparsiflorus |
| Amorphophallus spectabilis |
| Amorphophallus staudtii |
| Amorphophallus stuhlmannii subsp. stuhlmannii |
| Amorphophallus stuhlmannii subsp. congoensis |
| Amorphophallus subcymbiformis |
| Amorphophallus sumawongii |
| Amorphophallus suwidjianus |
| Amorphophallus sylvaticus |
| Amorphophallus symonianus |
| Amorphophallus synandrifer |
| Amorphophallus taurostigma |
| Amorphophallus tenuispadix |
| Amorphophallus tenuistylis |
| Amorphophallus teuszii |
| Amorphophallus thaiensis |
| Amorphophallus tinekeae |
| Amorphophallus titanum |
| Amorphophallus tonkinensis |
| Amorphophallus tuberculatus |
| Amorphophallus variabilis |
| Amorphophallus venustus |
| Amorphophallus verticillatus |
| Amorphophallus vogelianus |
| Amorphophallus xiei |
| Amorphophallus yuloensis |
| Amorphophallus yunnanensis |
| Amorphophallus zenkeri subsp. zenkeri |
| Amorphophallus zenkeri subsp. mannii |
| Amydrium hainanense |
| Amydrium humile |
| Amydrium medium |
| Amydrium sinense |
| Amydrium zippelianum |
| Anadendrum affine var. affine |
| Anadendrum affine var. semivestitum |
| Anadendrum angustifolium |
| Anadendrum badium |
| Anadendrum cordatum |
| Anadendrum ellipticum |
| Anadendrum griseum |
| Anadendrum latifolium |
| Anadendrum marcesovaginatum |
| Anadendrum marginatum |
| Anadendrum microstachyum |
| Anadendrum montanum |
| Anadendrum superans |
| Anaphyllopsis americana |
| Anaphyllopsis cururuana |
| Anaphyllopsis pinnata |
| Anaphyllum beddomei |
| Anaphyllum wightii |
| Anchomanes abbreviatus |
| Anchomanes boehmii |
| Anchomanes dalzielii |
| Anchomanes difformis |
| Anchomanes giganteus |
| Anchomanes nigritianus |
| Anthurium abelaezii |
| Anthurium acanthospadix |
| Anthurium acaule |
| Anthurium acebeyae |
| Anthurium achupallense |
| Anthurium acutangulum |
| Anthurium acutibacca |
| Anthurium acutifolium var. acutifolium |
| Anthurium acutifolium var. herrerae |
| Anthurium acutissimum |
| Anthurium acutum |
| Anthurium aduncum |
| Anthurium affine |
| Anthurium agnatum |
| Anthurium alatipedunculatum |
| Anthurium alatum |
| Anthurium albertiae |
| Anthurium albidum |
| Anthurium albispatha |
| Anthurium albobueyense |
| Anthurium albovirescens |
| Anthurium alcatrazense |
| Anthurium alegriasense |
| Anthurium algentryi |
| Anthurium alluriquinense |
| Anthurium alstonii |
| Anthurium alticola |
| Anthurium amargalense |
| Anthurium ameliae |
| Anthurium amnicola |
| Anthurium amoenum var. amoenum |
| Anthurium amoenum var. humile |
| Anthurium anceps |
| Anthurium anchicayense |
| Anthurium ancuashii |
| Anthurium andicola |
| Anthurium andinum |
| Anthurium andraeanum |
| Anthurium andreslovinense |
| Anthurium angelopolinense |
| Anthurium angosturense |
| Anthurium angustatum |
| Anthurium angustilaminatum subsp. angustilaminatum |
| Anthurium angustilaminatum subsp. cibuserpentis |
| Anthurium angustilobum |
| Anthurium angustisectum |
| Anthurium angustispadix |
| Anthurium anorianum |
| Anthurium antioquiense |
| Anthurium antonioanum |
| Anthurium antrophyoides |
| Anthurium apanui |
| Anthurium apaporanum |
| Anthurium apiaense |
| Anthurium arenasense |
| Anthurium argyrostachyum |
| Anthurium aripoense |
| Anthurium arisaemoides |
| Anthurium aristatum |
| Anthurium armeniense |
| Anthurium aroense |
| Anthurium arusiense |
| Anthurium asplundii |
| Anthurium atamainii |
| Anthurium atramentarium |
| Anthurium atropurpureum var. atropurpureum |
| Anthurium atropurpureum var. arenicola |
| Anthurium atropurpureum var. thomasii |
| Anthurium atroviride |
| Anthurium augustinum |
| Anthurium aureum |
| Anthurium auritum |
| Anthurium austin-smithii |
| Anthurium aylwardianum |
| Anthurium baguense |
| Anthurium bakeri |
| Anthurium balaoanum |
| Anthurium balslevii |
| Anthurium barbacoasense |
| Anthurium barclayanum |
| Anthurium barreranum |
| Anthurium barrieri |
| Anthurium barryi |
| Anthurium basirotundum |
| Anthurium bayae |
| Anthurium beckii |
| Anthurium bellum |
| Anthurium beltianum |
| Anthurium benktsparrei |
| Anthurium bernardii |
| Anthurium berriozabalense |
| Anthurium berryi |
| Anthurium besseae |
| Anthurium betanianum |
| Anthurium bicordoense |
| Anthurium bimarginatum |
| Anthurium binotii |
| Anthurium birdseyanum |
| Anthurium bittneri |
| Anthurium bocainense |
| Anthurium boekei |
| Anthurium bogneri |
| Anthurium bogotense |
| Anthurium bonplandii subsp. bonplandii |
| Anthurium bonplandii subsp. guayanum |
| Anthurium boosianum |
| Anthurium boudetii |
| Anthurium brachypodum |
| Anthurium bradeanum |
| Anthurium bragae |
| Anthurium bredemeyeri |
| Anthurium brenesii |
| Anthurium brent-berlinii |
| Anthurium brevipedunculatum |
| Anthurium brevipes |
| Anthurium breviscapum |
| Anthurium brevispadix |
| Anthurium brittonianum |
| Anthurium bromelicola subsp. bromelicola |
| Anthurium bromelicola subsp. bahiense |
| Anthurium brownii |
| Anthurium bucayanum |
| Anthurium buchtienii |
| Anthurium buganum |
| Anthurium bullianum |
| Anthurium bullosum |
| Anthurium burgeri |
| Anthurium bushii |
| Anthurium cabrerense |
| Anthurium cabuyalense |
| Anthurium cachabianum |
| Anthurium cainarachense |
| Anthurium caldodsonii |
| Anthurium calimense |
| Anthurium callejasii |
| Anthurium caloveboranum |
| Anthurium campii |
| Anthurium camposii |
| Anthurium canaliculatum |
| Anthurium candolleanum |
| Anthurium caperatum |
| Anthurium caraboboense |
| Anthurium caramantae |
| Anthurium carchiense |
| Anthurium cardenasii |
| Anthurium carinatum |
| Anthurium caripense |
| Anthurium carneospadix |
| Anthurium carnosum |
| Anthurium carpishense |
| Anthurium cartiense |
| Anthurium cartilagineum |
| Anthurium cataniapoense |
| Anthurium caucanum |
| Anthurium caucavallense |
| Anthurium caulorrhizum |
| Anthurium ceratiinum |
| Anthurium ceronii |
| Anthurium cerrateae |
| Anthurium cerrobaulense |
| Anthurium cerrocampanense |
| Anthurium cerropelonense |
| Anthurium cerropirrense |
| Anthurium chacoense |
| Anthurium chamberlainii |
| Anthurium chamulense subsp. chamulense |
| Anthurium chamulense subsp. oaxacanum |
| Anthurium chiapasense subsp. chiapasense |
| Anthurium chiapasense subsp. tlaxiacense |
| Anthurium chimborazense |
| Anthurium chinchipense |
| Anthurium chinimense |
| Anthurium chiriquense |
| Anthurium chocoense |
| Anthurium chorense |
| Anthurium chorranum |
| Anthurium chrysolithos |
| Anthurium churutense |
| Anthurium cinereopetiolatum |
| Anthurium cipoense |
| Anthurium circinatum |
| Anthurium cirinoi |
| Anthurium citrifolium |
| Anthurium clarinervium |
| Anthurium clarkei |
| Anthurium clathratum |
| Anthurium clavatum |
| Anthurium clavigerum |
| Anthurium cleistanthum |
| Anthurium clidemioides subsp. clidemioides |
| Anthurium clidemioides subsp. pacificum |
| Anthurium coclense |
| Anthurium cocornaense |
| Anthurium coerulescens |
| Anthurium cogolloanum |
| Anthurium coleomischum |
| Anthurium coleorrhiza |
| Anthurium collettianum |
| Anthurium collinsii |
| Anthurium colonchense |
| Anthurium colonense |
| Anthurium colonicum |
| Anthurium coloradense |
| Anthurium comtum |
| Anthurium concinnatum |
| Anthurium concolor |
| Anthurium conjunctum |
| Anthurium consimile |
| Anthurium consobrinum |
| Anthurium conspicuum |
| Anthurium constrictum |
| Anthurium conterminum |
| Anthurium corallinum |
| Anthurium cordatotriangulum |
| Anthurium cordatum |
| Anthurium cordiforme |
| Anthurium cordobense |
| Anthurium cordulatum |
| Anthurium coriaceum |
| Anthurium coripatense |
| Anthurium correae |
| Anthurium corrugatum |
| Anthurium cotejense |
| Anthurium cotobrusii |
| Anthurium cowanii |
| Anthurium crassifolium |
| Anthurium crassilaminum |
| Anthurium crassinervium var. crassinervium |
| Anthurium crassinervium var. caatingae |
| Anthurium crassiradix var. crassiradix |
| Anthurium crassiradix var. purpureospadix |
| Anthurium crassitepalum |
| Anthurium crassivenium |
| Anthurium cremersii |
| Anthurium crenatum |
| Anthurium croatii |
| Anthurium crystallinum |
| Anthurium cuasicanum |
| Anthurium cubense |
| Anthurium cucullispathum |
| Anthurium cultrifolium |
| Anthurium cupreonitens |
| Anthurium cupreum |
| Anthurium cupulispathum |
| Anthurium curicuriariense |
| Anthurium curtispadix |
| Anthurium curvilaminum |
| Anthurium curvispadix |
| Anthurium cuspidatum |
| Anthurium cuspidiferum |
| Anthurium cutucuense |
| Anthurium cuyabenoense |
| Anthurium cylindratum |
| Anthurium cymbiforme |
| Anthurium cymbispatha |
| Anthurium daguense |
| Anthurium darcyi |
| Anthurium davidsei |
| Anthurium davidsoniae |
| Anthurium debilipeltatum |
| Anthurium debilis |
| Anthurium decurrens |
| Anthurium deflexum |
| Anthurium delannayi |
| Anthurium dendrobates |
| Anthurium denudatum |
| Anthurium diazii |
| Anthurium digitatum |
| Anthurium diversicaudex |
| Anthurium dolichocnemum |
| Anthurium dolichophyllum |
| Anthurium dolichostachyum |
| Anthurium dombeyanum |
| Anthurium dominicense |
| Anthurium dorbayae |
| Anthurium draconopterum |
| Anthurium dressleri |
| Anthurium dukei |
| Anthurium durandii |
| Anthurium dussii |
| Anthurium dwyeri |
| Anthurium dylanii |
| Anthurium ecuadorense |
| Anthurium effusilobum subsp. effusilobum |
| Anthurium effusilobum subsp. pallidispadix |
| Anthurium effusispathum |
| Anthurium eggersii |
| Anthurium eichleri |
| Anthurium emarginatum |
| Anthurium eminens subsp. eminens |
| Anthurium eminens subsp. longispadix |
| Anthurium ensifolium |
| Anthurium ericae |
| Anthurium ernestii var. ernestii |
| Anthurium ernestii var. oellgaardii |
| Anthurium erskinei |
| Anthurium erythrostachyum |
| Anthurium esmeraldense |
| Anthurium espinae |
| Anthurium eximium |
| Anthurium expansum |
| Anthurium exstipulatum |
| Anthurium fasciale |
| Anthurium fatoense |
| Anthurium faustomirandae |
| Anthurium fendleri |
| Anthurium fernandezii |
| Anthurium filiforme |
| Anthurium flavescens |
| Anthurium flavidum |
| Anthurium flavolineatum |
| Anthurium flavoviride |
| Anthurium flexile subsp. flexile |
| Anthurium flexile subsp. muelleri |
| Anthurium folsomianum |
| Anthurium fontellanum |
| Anthurium fontoides |
| Anthurium foreroanum |
| Anthurium forgetii |
| Anthurium formosum |
| Anthurium fornicifolium |
| Anthurium fosteri |
| Anthurium fragae |
| Anthurium fragrans |
| Anthurium fragrantissimum |
| Anthurium fraseri |
| Anthurium friedrichsthalii |
| Anthurium funiferum |
| Anthurium furcatum |
| Anthurium fuscopunctatum |
| Anthurium fusiforme |
| Anthurium gaffurii |
| Anthurium galactospadix |
| Anthurium galeanoae |
| Anthurium galeottii |
| Anthurium galileanum |
| Anthurium gaskinii |
| Anthurium gaudichaudianum |
| Anthurium gehrigeri |
| Anthurium geitnerianum |
| Anthurium genferryae |
| Anthurium geniculatum |
| Anthurium gentryi |
| Anthurium giganteum |
| Anthurium ginesii |
| Anthurium giraldoi |
| Anthurium gladiifolium |
| Anthurium glanduligerum |
| Anthurium glaucophyllum |
| Anthurium glaucospadix |
| Anthurium globosum |
| Anthurium gomesianum |
| Anthurium gonzalezii |
| Anthurium gracile |
| Anthurium gracililaminum |
| Anthurium gracilipedunculatum |
| Anthurium gracilispadix |
| Anthurium grande |
| Anthurium grandicataphyllum |
| Anthurium grandifolium |
| Anthurium grex-avium |
| Anthurium gualeanum |
| Anthurium guanacense |
| Anthurium guanchezii |
| Anthurium guatemalense |
| Anthurium guayaquilense |
| Anthurium gustavii |
| Anthurium gymnopus |
| Anthurium hacumense |
| Anthurium hagsaterianum |
| Anthurium halmoorei |
| Anthurium hamiltonii |
| Anthurium hammelii |
| Anthurium harrisii |
| Anthurium hastifolium |
| Anthurium hatschbachii |
| Anthurium hebetatilaminum |
| Anthurium hebetatum |
| Anthurium herthae |
| Anthurium hieronymi |
| Anthurium hinoideum |
| Anthurium hodgei |
| Anthurium hoehnei |
| Anthurium hoffmannii |
| Anthurium holm-nielsenii |
| Anthurium holquinianum |
| Anthurium hookeri |
| Anthurium hornitense |
| Anthurium huacamayoense |
| Anthurium huallagense |
| Anthurium huampamiense |
| Anthurium huanucense |
| Anthurium huashikatii |
| Anthurium huautlense |
| Anthurium huberi |
| Anthurium huixtlense |
| Anthurium humboldtianum subsp. humboldtianum |
| Anthurium humboldtianum subsp. viridispadix |
| Anthurium humoense |
| Anthurium hutchisonii |
| Anthurium hygrophilum |
| Anthurium ianthinopodum |
| Anthurium icanense |
| Anthurium idmense |
| Anthurium illepidum |
| Anthurium iltisii |
| Anthurium imperiale |
| Anthurium impolitum |
| Anthurium incomptum |
| Anthurium inconspicuum |
| Anthurium incurvatum |
| Anthurium incurvum |
| Anthurium infectorium |
| Anthurium intermedium |
| Anthurium interruptum |
| Anthurium inzanum |
| Anthurium ionanthum |
| Anthurium iramireziae |
| Anthurium isidroense |
| Anthurium jaramilloi |
| Anthurium jefense |
| Anthurium jenmanii |
| Anthurium jesusii |
| Anthurium jilekii |
| Anthurium jimenae |
| Anthurium joaquinense |
| Anthurium johnmackii |
| Anthurium johnsoniae |
| Anthurium josei |
| Anthurium julianii |
| Anthurium julospadix |
| Anthurium jureianum |
| Anthurium kajekai |
| Anthurium kallunkiae |
| Anthurium kamemotoanum |
| Anthurium karstenianum |
| Anthurium kastelskii |
| Anthurium kayapii |
| Anthurium knappiae |
| Anthurium krukovii |
| Anthurium kugkumasii |
| Anthurium kunayalense |
| Anthurium kunthii var. kunthii |
| Anthurium kunthii var. cylindricum |
| Anthurium kusuense |
| Anthurium lacerdae |
| Anthurium laciniosum |
| Anthurium lactifructum |
| Anthurium lancea |
| Anthurium lancetillense |
| Anthurium lancifolium var. lancifolium |
| Anthurium lancifolium var. albifructum |
| Anthurium langendoenii |
| Anthurium langsdorffii |
| Anthurium lanjouwii |
| Anthurium latemarginatum |
| Anthurium latissimum |
| Anthurium laucheanum |
| Anthurium lautum |
| Anthurium lechlerianum |
| Anthurium lehmannii |
| Anthurium lennartii |
| Anthurium lentii |
| Anthurium leonianum |
| Anthurium leonii |
| Anthurium leptocaule |
| Anthurium leuconeurum |
| Anthurium leveaui |
| Anthurium lezamae |
| Anthurium lhotzkyanum |
| Anthurium libanoense |
| Anthurium licium |
| Anthurium lievenii |
| Anthurium ligulare |
| Anthurium lilacinum |
| Anthurium limonense |
| Anthurium lindenianum |
| Anthurium lindmanianum |
| Anthurium lineolatum |
| Anthurium linganii |
| Anthurium lingua |
| Anthurium linguifolium |
| Anthurium llanense |
| Anthurium llewellynii |
| Anthurium lloense |
| Anthurium loefgrenii |
| Anthurium lojtnantii |
| Anthurium longicaudatum |
| Anthurium longicuspidatum |
| Anthurium longifolium |
| Anthurium longigeniculatum |
| Anthurium longipeltatum |
| Anthurium longipes |
| Anthurium longispadiceum |
| Anthurium longissimilobum |
| Anthurium longissimum subsp. longissimum |
| Anthurium longissimum subsp. nirguense |
| Anthurium longistamineum |
| Anthurium longistipitatum |
| Anthurium longiusculum |
| Anthurium loretense |
| Anthurium louisii |
| Anthurium lucens |
| Anthurium lucidum |
| Anthurium lucioi |
| Anthurium lucorum |
| Anthurium luschnathianum |
| Anthurium lutescens |
| Anthurium luteynii |
| Anthurium luxurians |
| Anthurium lygrum |
| Anthurium lynniae |
| Anthurium maasii |
| Anthurium macarenense |
| Anthurium macbridei |
| Anthurium macdanielii |
| Anthurium machetioides |
| Anthurium macleanii |
| Anthurium macphersonii |
| Anthurium macrocephalum |
| Anthurium macrolonchium |
| Anthurium macrophyllum |
| Anthurium macropodum |
| Anthurium macrospadix |
| Anthurium macrourum |
| Anthurium maculosum |
| Anthurium madisonianum |
| Anthurium magdae |
| Anthurium magnificum |
| Anthurium magnifolium |
| Anthurium maguirei |
| Anthurium malagaense |
| Anthurium malianum |
| Anthurium manabianum |
| Anthurium mancuniense |
| Anthurium manuanum |
| Anthurium marense |
| Anthurium margaricarpum |
| Anthurium marginellum |
| Anthurium mariae |
| Anthurium maricense |
| Anthurium marinoanum |
| Anthurium marleenianum |
| Anthurium marmoratum |
| Anthurium martianum |
| Anthurium masfense |
| Anthurium maximiliani |
| Anthurium maximum |
| Anthurium megapetiolatum |
| Anthurium melastomatis |
| Anthurium membranaceum |
| Anthurium mendietae |
| Anthurium merlei |
| Anthurium metallicum |
| Anthurium miaziense |
| Anthurium michelii |
| Anthurium microphyllum |
| Anthurium microspadix |
| Anthurium minarum |
| Anthurium mindense |
| Anthurium miniatum |
| Anthurium modicum |
| Anthurium molaui |
| Anthurium molle |
| Anthurium montanum |
| Anthurium monteverdense |
| Anthurium monticola var. monticola |
| Anthurium monticola var. attenuatum |
| Anthurium monzonense |
| Anthurium moonenii |
| Anthurium morae |
| Anthurium morii |
| Anthurium moronense |
| Anthurium mostaceroi |
| Anthurium mourae |
| Anthurium multinervium |
| Anthurium multisulcatum |
| Anthurium munchiquense |
| Anthurium myosuroides |
| Anthurium myosurus |
| Anthurium nakamurae |
| Anthurium nangaritense |
| Anthurium nanum |
| Anthurium napaeum |
| Anthurium narinoense |
| Anthurium navasii |
| Anthurium naviculare |
| Anthurium nelsonii |
| Anthurium nemorale |
| Anthurium nemoricola |
| Anthurium nervatum |
| Anthurium nicolasianum |
| Anthurium nigrescens |
| Anthurium nigropunctatum |
| Anthurium niqueanum |
| Anthurium nitens |
| Anthurium nitidulum |
| Anthurium nitidum |
| Anthurium nizandense |
| Anthurium novitaense |
| Anthurium nubicola |
| Anthurium nutibarense |
| Anthurium nymphaeifolium |
| Anthurium obliquatum |
| Anthurium oblongocordatum |
| Anthurium obpyriforme |
| Anthurium obscurinervium |
| Anthurium obtusatum |
| Anthurium obtusifolium |
| Anthurium obtusilobum |
| Anthurium obtusum subsp. obtusum |
| Anthurium obtusum subsp. puntarenense |
| Anthurium occidentale |
| Anthurium ochranthum |
| Anthurium ochreatum |
| Anthurium ocotepecense |
| Anthurium oerstedianum |
| Anthurium oreodoxa |
| Anthurium oreophilum |
| Anthurium organense |
| Anthurium orientale |
| Anthurium orteganum |
| Anthurium ottobuchtienii |
| Anthurium ottonis |
| Anthurium ovatifolium |
| Anthurium oxyanthum |
| Anthurium oxybelium |
| Anthurium oxycarpum |
| Anthurium oxyphyllum |
| Anthurium oxystachyum |
| Anthurium pachylaminum |
| Anthurium pachyspathum |
| Anthurium pageanum |
| Anthurium palacioanum |
| Anthurium palenquense |
| Anthurium pallatangense |
| Anthurium pallens |
| Anthurium pallidicaudex |
| Anthurium pallidiflorum |
| Anthurium palmarense |
| Anthurium palmatum |
| Anthurium paludosum |
| Anthurium panamense |
| Anthurium panduriforme |
| Anthurium papillilaminum |
| Anthurium paradisicum |
| Anthurium paraguasense |
| Anthurium paraguayense var. paraguayense |
| Anthurium paraguayense var. coroicoanum |
| Anthurium parambae |
| Anthurium parasiticum |
| Anthurium pariense |
| Anthurium parile |
| Anthurium parvispathum |
| Anthurium parvum |
| Anthurium pastasanum |
| Anthurium patens |
| Anthurium pauciflorum |
| Anthurium paucinerve |
| Anthurium payaminoense |
| Anthurium pedatoradiatum subsp. pedatoradiatum |
| Anthurium pedatoradiatum subsp. helleborifolium |
| Anthurium pedatum |
| Anthurium pedunculare |
| Anthurium pellucidopunctatum |
| Anthurium peltatum |
| Anthurium peltigerum |
| Anthurium penae |
| Anthurium pendens |
| Anthurium pendulifolium |
| Anthurium penningtonii |
| Anthurium pentaphyllum var. pentaphyllum |
| Anthurium pentaphyllum var. bombacifolium |
| Anthurium perijanum |
| Anthurium perviride |
| Anthurium pescadilloense |
| Anthurium petrophilum |
| Anthurium phyllobaris |
| Anthurium pichinchae |
| Anthurium pichindense |
| Anthurium pilonense |
| Anthurium pinkleyi |
| Anthurium pirottae |
| Anthurium pirrense |
| Anthurium pittieri var. pittieri |
| Anthurium pittieri var. fogdeniorum |
| Anthurium pittieri var. morii |
| Anthurium piurensis |
| Anthurium plantagineum |
| Anthurium platyglossum |
| Anthurium platyrhizum |
| Anthurium plowmanii |
| Anthurium plurisulcatum |
| Anthurium pluviaticum |
| Anthurium podophyllum |
| Anthurium pohlianum |
| Anthurium polydactylum |
| Anthurium polynervium |
| Anthurium polyneuron |
| Anthurium polyphlebium |
| Anthurium polyschistum |
| Anthurium polystictum |
| Anthurium porcesitoense |
| Anthurium potarense |
| Anthurium pradoense |
| Anthurium praealtum |
| Anthurium pranceanum |
| Anthurium prolatum |
| Anthurium prominens |
| Anthurium promininerve |
| Anthurium protensum subsp. protensum |
| Anthurium protensum subsp. arcuatum |
| Anthurium pseudospectabile |
| Anthurium psilostachyum |
| Anthurium ptarianum |
| Anthurium puberulinervium |
| Anthurium puberulum |
| Anthurium pucayacuense |
| Anthurium pulcachense |
| Anthurium pulchellum |
| Anthurium pulidoae |
| Anthurium pulverulentum var. pulverulentum |
| Anthurium pulverulentum var. adsimile |
| Anthurium punctatum |
| Anthurium punkuyocense |
| Anthurium purdieanum |
| Anthurium purpureospathum |
| Anthurium purpureum |
| Anthurium queirozianum |
| Anthurium quinindense |
| Anthurium quinquenervium |
| Anthurium quinquesulcatum |
| Anthurium quipuscoae |
| Anthurium radiatum |
| Anthurium radicans |
| Anthurium raimundii |
| Anthurium ramoncaracasii |
| Anthurium ramonense |
| Anthurium ramosense |
| Anthurium ramosii |
| Anthurium ranchoanum |
| Anthurium ravenii |
| Anthurium recavum |
| Anthurium redolens |
| Anthurium reflexinervium |
| Anthurium regale |
| Anthurium remotigeniculatum |
| Anthurium remotum |
| Anthurium renteriae |
| Anthurium resectum |
| Anthurium reticulatum |
| Anthurium retiferum |
| Anthurium rhizophorum |
| Anthurium rhodorhizum |
| Anthurium ribeiroi |
| Anthurium rigidifolium |
| Anthurium rimbachii |
| Anthurium riodocense |
| Anthurium riofrioi |
| Anthurium riograndicola |
| Anthurium riojaense |
| Anthurium rionegrense |
| Anthurium riparium |
| Anthurium rivulare |
| Anthurium rodrigueziae |
| Anthurium roezlii |
| Anthurium rojasiae |
| Anthurium roraimense |
| Anthurium roseospadix |
| Anthurium rotundatum |
| Anthurium rotundilobum |
| Anthurium rotundistigmatum |
| Anthurium rubrifructum |
| Anthurium rubrivellus |
| Anthurium rugulosum |
| Anthurium rupestre |
| Anthurium rupicola |
| Anthurium rzedowskii |
| Anthurium saccardoi |
| Anthurium sagawae |
| Anthurium sagittale |
| Anthurium sagittaria |
| Anthurium sagittatum |
| Anthurium sagittellum |
| Anthurium salgarense |
| Anthurium salvadorense |
| Anthurium salvinii |
| Anthurium samamaense |
| Anthurium sanctifidense |
| Anthurium sanguineum |
| Anthurium santaritensis |
| Anthurium santiagoense |
| Anthurium sapense |
| Anthurium sarmentosum |
| Anthurium sarukhanianum |
| Anthurium scaberulum |
| Anthurium scandens subsp. scandens |
| Anthurium scandens subsp. pusillum |
| Anthurium scherzerianum |
| Anthurium schlechtendalii subsp. schlechtendalii |
| Anthurium schlechtendalii subsp. jimenezii |
| Anthurium schottianum |
| Anthurium schunkei |
| Anthurium sebastianense |
| Anthurium seibertii |
| Anthurium seleri |
| Anthurium sellowianum |
| Anthurium septuplinervium |
| Anthurium shinumas |
| Anthurium siccisilvarum |
| Anthurium sidneyi |
| Anthurium sierpense |
| Anthurium signatum |
| Anthurium silanchense |
| Anthurium silverstonei |
| Anthurium silvicola |
| Anthurium silvigaudens |
| Anthurium simonii |
| Anthurium simpsonii |
| Anthurium sinuatum |
| Anthurium siqueirae |
| Anthurium smaragdinum |
| Anthurium smithii |
| Anthurium sodiroanum |
| Anthurium soejartoi subsp. soejartoi |
| Anthurium soejartoi subsp. ascendens |
| Anthurium solitarium |
| Anthurium solomonii |
| Anthurium soukupii |
| Anthurium sparreorum |
| Anthurium spathiphyllum |
| Anthurium spathulifolium |
| Anthurium spectabile |
| Anthurium splendidum |
| Anthurium standleyi |
| Anthurium stephanii |
| Anthurium stipitatum |
| Anthurium straminopetiolum |
| Anthurium striatipes |
| Anthurium striatum |
| Anthurium striolatum |
| Anthurium stuebelii |
| Anthurium subaequans |
| Anthurium subcarinatum |
| Anthurium subcaudatum |
| Anthurium subcoerulescens |
| Anthurium subcordatum subsp. subcordatum |
| Anthurium subcordatum subsp. chlorocardium |
| Anthurium subhastatum |
| Anthurium subovatum |
| Anthurium subrotundum |
| Anthurium subsagittatum |
| Anthurium subscriptum |
| Anthurium subsignatum |
| Anthurium subtriangulare |
| Anthurium subtrilobum |
| Anthurium subtruncatum |
| Anthurium subulatum |
| Anthurium sucrii |
| Anthurium sulcatum |
| Anthurium superbum subsp. superbum |
| Anthurium superbum subsp. brentberlinii |
| Anthurium supianum |
| Anthurium supraglandulum |
| Anthurium suramaense |
| Anthurium sylvestre |
| Anthurium sytsmae |
| Anthurium tacarcunense |
| Anthurium tachiranum |
| Anthurium talmonii |
| Anthurium tamaense |
| Anthurium tarapotense |
| Anthurium tatei |
| Anthurium teimosoanum |
| Anthurium tenaense |
| Anthurium tenerum |
| Anthurium tenuicaule |
| Anthurium tenuifolium |
| Anthurium tenuispica |
| Anthurium teribense |
| Anthurium ternifolium |
| Anthurium terryae |
| Anthurium testaceum |
| Anthurium thompsoniae |
| Anthurium thrinax |
| Anthurium tikunorum |
| Anthurium tilaranense |
| Anthurium timplowmanii |
| Anthurium titanium |
| Anthurium tolimense |
| Anthurium tomasiae |
| Anthurium tonduzii |
| Anthurium tonianum |
| Anthurium torraense |
| Anthurium treleasei |
| Anthurium tremulum |
| Anthurium trianae |
| Anthurium tricarinatum |
| Anthurium triciafrankiae |
| Anthurium trifidum |
| Anthurium trilobum |
| Anthurium trinervium |
| Anthurium triphyllum |
| Anthurium trisectum |
| Anthurium truncatulum |
| Anthurium truncatum |
| Anthurium truncicola |
| Anthurium tsamajainii |
| Anthurium tunquii |
| Anthurium tutense |
| Anthurium tysonii |
| Anthurium uasadiensis |
| Anthurium uleanum var. uleanum |
| Anthurium uleanum var. nanayense |
| Anthurium umbraculum |
| Anthurium umbricola |
| Anthurium umbrosum |
| Anthurium unense |
| Anthurium upalaense |
| Anthurium urbanii |
| Anthurium urvilleanum |
| Anthurium utleyorum |
| Anthurium validifolium |
| Anthurium validinervium |
| Anthurium vallense |
| Anthurium vanderknaapii |
| Anthurium variegatum |
| Anthurium variilobum |
| Anthurium vaupesianum |
| Anthurium veitchii |
| Anthurium velutinum |
| Anthurium venadoense |
| Anthurium venosum |
| Anthurium ventanasense |
| Anthurium verapazense |
| Anthurium verrucosum |
| Anthurium versicolor var. versicolor |
| Anthurium versicolor var. azuayense |
| Anthurium vestitum |
| Anthurium victorii |
| Anthurium vientense |
| Anthurium vinillense |
| Anthurium viridescens |
| Anthurium viridispathum |
| Anthurium vittariifolium |
| Anthurium vomeriforme |
| Anthurium wagenerianum |
| Anthurium wallisii |
| Anthurium walujewii |
| Anthurium warintsense |
| Anthurium warocqueanum |
| Anthurium watermaliense |
| Anthurium wattii |
| Anthurium weberbaueri |
| Anthurium wedelianum subsp. wedelianum |
| Anthurium wedelianum subsp. viridispadix |
| Anthurium wendlingeri |
| Anthurium werffii |
| Anthurium werneri |
| Anthurium whitmorei |
| Anthurium willdenowii |
| Anthurium willifordii |
| Anthurium wintersii |
| Anthurium wurdackii |
| Anthurium xanthoneurum |
| Anthurium xanthophylloides |
| Anthurium yamayakatense |
| Anthurium yarumalense |
| Anthurium yatacuense |
| Anthurium yetlense |
| Anthurium yungasense |
| Anthurium yurimaguense |
| Anthurium yutajense |
| Anthurium zappiae |
| Anthurium zeneidae |
| Anthurium zuloagae |
| Anubias afzelii |
| Anubias barteri var. barteri |
| Anubias barteri var. angustifolia |
| Anubias barteri var. caladiifolia |
| Anubias barteri var. glabra |
| Anubias barteri var. nana |
| Anubias gigantea |
| Anubias gilletii |
| Anubias gracilis |
| Anubias hastifolia |
| Anubias heterophylla |
| Anubias pynaertii |
| Apoballis acuminatissima |
| Apoballis belophylla |
| Apoballis brevipes |
| Apoballis grandiflora |
| Apoballis hastifolia |
| Apoballis javanica |
| Apoballis longicaulis |
| Apoballis mutata |
| Apoballis okadae |
| Apoballis ovata |
| Apoballis rupestris |
| Apoballis sagittifolia |
| Aridarum borneense |
| Aridarum burttii |
| Aridarum caulescens |
| Aridarum crassum |
| Aridarum incavatum |
| Aridarum minimum |
| Aridarum montanum |
| Aridarum nicolsonii |
| Aridarum purseglovei |
| Aridarum rostratum |
| Ariopsis peltata |
| Ariopsis protanthera |
| Arisaema abei |
| Arisaema aequinoctiale |
| Arisaema agasthyanum |
| Arisaema album |
| Arisaema amurense |
| Arisaema anomalum |
| Arisaema aprile |
| Arisaema aridum |
| Arisaema asperatum |
| Arisaema auriculatum |
| Arisaema austroyunnanense |
| Arisaema averyanovii |
| Arisaema balansae |
| Arisaema bannaense |
| Arisaema barbatum |
| Arisaema barnesii |
| Arisaema bockii |
| Arisaema bonatianum |
| Arisaema bottae |
| Arisaema brucei |
| Arisaema calcareum |
| Arisaema candidissimum |
| Arisaema caudatum |
| Arisaema chuanxiense |
| Arisaema chumponense |
| Arisaema ciliatum |
| Arisaema clavatum |
| Arisaema concinnum |
| Arisaema condaoense |
| Arisaema consanguineum subsp. consanguineum |
| Arisaema consanguineum subsp. kelung-insulare |
| Arisaema constrictum |
| Arisaema cordatum |
| Arisaema costatum |
| Arisaema cucullatum |
| Arisaema dahaiense |
| Arisaema decipiens |
| Arisaema dracontium |
| Arisaema echinatum |
| Arisaema echinoides |
| Arisaema ehimense |
| Arisaema elephas |
| Arisaema enneaphyllum |
| Arisaema erubescens |
| Arisaema exappendiculatum |
| Arisaema fargesii |
| Arisaema filiforme |
| Arisaema fimbriatum subsp. fimbriatum |
| Arisaema fimbriatum subsp. bakerianum |
| Arisaema flavum subsp. flavum |
| Arisaema flavum subsp. tibeticum |
| Arisaema formosanum |
| Arisaema franchetianum |
| Arisaema fraternum |
| Arisaema galeatum |
| Arisaema garrettii |
| Arisaema ghaticum |
| Arisaema grapsospadix |
| Arisaema griffithii |
| Arisaema hainanense |
| Arisaema handelii |
| Arisaema heterocephalum subsp. heterocephalum |
| Arisaema heterocephalum subsp. okinawaense |
| Arisaema heterophyllum |
| Arisaema hunanense |
| Arisaema ilanense |
| Arisaema inclusum |
| Arisaema intermedium |
| Arisaema ishizuchiense subsp. ishizuchiense |
| Arisaema ishizuchiense subsp. brevicollum |
| Arisaema iyoanum |
| Arisaema jacquemontii |
| Arisaema jethompsonii |
| Arisaema jingdongense |
| Arisaema kawashimae |
| Arisaema kerrii |
| Arisaema kishidae |
| Arisaema kiushianum |
| Arisaema kuratae |
| Arisaema lackneri |
| Arisaema laminatum |
| Arisaema leschenaultii |
| Arisaema lichiangense |
| Arisaema lidaense |
| Arisaema lihengianum |
| Arisaema limbatum |
| Arisaema linearifolium |
| Arisaema lingyunense |
| Arisaema lobatum |
| Arisaema longipedunculatum |
| Arisaema macrospathum |
| Arisaema maekawae |
| Arisaema mairei |
| Arisaema maximowiczii subsp. maximowiczii |
| Arisaema maximowiczii subsp. tashiroi |
| Arisaema maxwellii |
| Arisaema meleagris |
| Arisaema menglaense |
| Arisaema microspadix |
| Arisaema mildbraedii |
| Arisaema minamitanii |
| Arisaema minus |
| Arisaema monophyllum var. monophyllum |
| Arisaema monophyllum var. atrolinguum |
| Arisaema mooneyanum |
| Arisaema muratae |
| Arisaema muricaudatum |
| Arisaema murrayi var. murrayi |
| Arisaema murrayi var. sahyadricum |
| Arisaema murrayi var. sonubeniae |
| Arisaema nagiense |
| Arisaema nambae |
| Arisaema negishii |
| Arisaema nepenthoides |
| Arisaema nikoense var. nikoense |
| Arisaema nikoense var. australe |
| Arisaema nikoense var. kaimontanum |
| Arisaema nilamburense |
| Arisaema odoratum |
| Arisaema ogatae |
| Arisaema omkoiense |
| Arisaema ornatum |
| Arisaema ovale var. ovale |
| Arisaema ovale var. inaense |
| Arisaema ovale var. sadoense |
| Arisaema pachystachyum |
| Arisaema pallidum |
| Arisaema parisifolia |
| Arisaema parvum |
| Arisaema pattaniense |
| Arisaema penicillatum |
| Arisaema petelotii |
| Arisaema petiolulatum |
| Arisaema pianmaense |
| Arisaema pingbianense |
| Arisaema polyphyllum |
| Arisaema prazeri |
| Arisaema propinquum |
| Arisaema psittacus |
| Arisaema quinatum |
| Arisaema quinquelobatum |
| Arisaema ramulosum |
| Arisaema ringens |
| Arisaema rostratum |
| Arisaema roxburghii |
| Arisaema rubrirhizomatum |
| Arisaema ruwenzoricum |
| Arisaema sachalinense |
| Arisaema saddlepeakense |
| Arisaema sarracenioides |
| Arisaema saxatile |
| Arisaema sazensoo |
| Arisaema schimperianum |
| Arisaema scortechinii |
| Arisaema seppikoense |
| Arisaema serratum var. serratum |
| Arisaema serratum var. izuense |
| Arisaema serratum var. mayebarae |
| Arisaema serratum var. suwoense |
| Arisaema setosum |
| Arisaema siamicum |
| Arisaema siangense |
| Arisaema sikokianum |
| Arisaema silvestrii |
| Arisaema sinii |
| Arisaema sizemoreae |
| Arisaema smitinandii |
| Arisaema somalense |
| Arisaema souliei |
| Arisaema speciosum var. speciosum |
| Arisaema speciosum var. mirabile |
| Arisaema speciosum var. ziroense |
| Arisaema sukotaiense |
| Arisaema taiwanense var. taiwanense |
| Arisaema taiwanense var. brevipedunculatum |
| Arisaema tengtsungense |
| Arisaema ternatipartitum |
| Arisaema thunbergii subsp. thunbergii |
| Arisaema thunbergii subsp. autumnale |
| Arisaema thunbergii subsp. geomundoense |
| Arisaema thunbergii subsp. urashima |
| Arisaema tortuosum subsp. tortuosum |
| Arisaema tortuosum var. neglectum |
| Arisaema tortuosum subsp. sivadasanii |
| Arisaema tosaense |
| Arisaema translucens |
| Arisaema triphyllum |
| Arisaema tsangpoense |
| Arisaema tuberculatum |
| Arisaema ulugurense |
| Arisaema umbrinum |
| Arisaema undulatifolium subsp. undulatifolium |
| Arisaema undulatifolium subsp. uwajimense |
| Arisaema utile |
| Arisaema vexillatum |
| Arisaema victoriae |
| Arisaema wangmoense |
| Arisaema wardii |
| Arisaema wattii |
| Arisaema wilsonii |
| Arisaema wrayi |
| Arisaema xuanweiense |
| Arisaema yamatense subsp. yamatense |
| Arisaema yamatense subsp. sugimotoi |
| Arisaema yanxianum |
| Arisaema yunnanense |
| Arisaema zhui |
| Arisarum proboscideum |
| Arisarum simorrhinum |
| Arisarum vulgare subsp. vulgare |
| Arisarum vulgare subsp. clusii |
| Arisarum vulgare subsp. hastatum |
| Arophyton buchetii |
| Arophyton crassifolium |
| Arophyton humbertii |
| Arophyton pedatum |
| Arophyton rhizomatosum |
| Arophyton simplex |
| Arophyton tripartitum var. tripartitum |
| Arophyton tripartitum var. masoalaense |
| Arum alpinariae |
| Arum apulum |
| Arum balansanum |
| Arum besserianum |
| Arum byzantinum |
| Arum concinnatum |
| Arum creticum |
| Arum cylindraceum subsp. cylindraceum |
| Arum cylindraceum subsp. pitsyllianum |
| Arum cyrenaicum |
| Arum dioscoridis var. dioscoridis |
| Arum dioscoridis var. cyprium |
| Arum dioscoridis var. philistaeum |
| Arum dioscoridis var. syriacum |
| Arum elongatum |
| Arum euxinum |
| Arum gratum |
| Arum hainesii |
| Arum hygrophilum |
| Arum idaeum |
| Arum italicum subsp. italicum |
| Arum italicum subsp. albispathum |
| Arum italicum subsp. canariense |
| Arum italicum subsp. neglectum |
| Arum jacquemontii |
| Arum korolkowii |
| Arum lucanum |
| Arum maculatum |
| Arum megobrebi |
| Arum nigrum |
| Arum orientale subsp. orientale |
| Arum orientale subsp. longispathum |
| Arum palaestinum |
| Arum pictum |
| Arum purpureospathum |
| Arum rupicola var. rupicola |
| Arum rupicola var. virescens |
| Arum sintenisii |
| Asterostigma cryptostylum |
| Asterostigma cubense |
| Asterostigma lividum |
| Asterostigma lombardii |
| Asterostigma luschnathianum |
| Asterostigma reticulatum |
| Asterostigma riedelianum |
| Asterostigma tweedieanum |
| Bakoa brevipedunculata |
| Bakoa lucens |
| Bakoa nakamotoi |
| Biarum aleppicum |
| Biarum angustatum |
| Biarum auraniticum |
| Biarum bovei |
| Biarum carduchorum |
| Biarum carratracense |
| Biarum crispulum |
| Biarum davisii |
| Biarum dispar |
| Biarum ditschianum |
| Biarum eximium |
| Biarum fraasianum |
| Biarum kotschyi |
| Biarum marmarisense |
| Biarum mendax |
| Biarum olivieri |
| Biarum pyrami var. pyrami |
| Biarum pyrami var. serotinum |
| Biarum rhopalospadix |
| Biarum straussii |
| Biarum syriacum |
| Biarum tenuifolium subsp. tenuifolium |
| Biarum tenuifolium subsp. abbreviatum |
| Biarum tenuifolium subsp. arundanum |
| Biarum tenuifolium subsp. galianii |
| Biarum tenuifolium subsp. idomenaeum |
| Biarum tenuifolium subsp. zelebori |
| Bognera recondita |
| Bucephalandra gigantea |
| Bucephalandra motleyana |
| Caladium andreanum |
| Caladium bicolor |
| Caladium clavatum |
| Caladium coerulescens |
| Caladium humboldtii |
| Caladium lindenii |
| Caladium macrotites |
| Caladium picturatum |
| Caladium praetermissum |
| Caladium schomburgkii |
| Caladium smaragdinum |
| Caladium steyermarkii |
| Caladium ternatum |
| Caladium tuberosum |
| Calla palustris |
| Callopsis volkensii |
| Carlephyton diegoense |
| Carlephyton glaucophyllum |
| Carlephyton madagascariense |
| Cercestis afzelii |
| Cercestis camerunensis |
| Cercestis congoensis |
| Cercestis dinklagei |
| Cercestis hepperi |
| Cercestis ivorensis |
| Cercestis kamerunianus |
| Cercestis mirabilis |
| Cercestis sagittatus |
| Cercestis taiensis |
| Chlorospatha amalfiensis |
| Chlorospatha antioquiensis |
| Chlorospatha atropurpurea |
| Chlorospatha besseae |
| Chlorospatha betancurii |
| Chlorospatha bogneri |
| Chlorospatha callejasii |
| Chlorospatha castula |
| Chlorospatha cogolloi |
| Chlorospatha corrugata |
| Chlorospatha croatiana subsp. croatiana |
| Chlorospatha croatiana subsp. enneaphylla |
| Chlorospatha cutucuensis |
| Chlorospatha dodsonii |
| Chlorospatha feuersteiniae |
| Chlorospatha gentryi |
| Chlorospatha hammeliana |
| Chlorospatha hastifolia |
| Chlorospatha ilensis |
| Chlorospatha kolbii |
| Chlorospatha kressii |
| Chlorospatha lehmannii |
| Chlorospatha longipoda |
| Chlorospatha luteynii |
| Chlorospatha macphersonii |
| Chlorospatha mirabilis |
| Chlorospatha nicolsonii |
| Chlorospatha planadensis |
| Chlorospatha ricaurtensis |
| Colletogyne perrieri |
| Colocasia affinis |
| Colocasia antiquorum |
| Colocasia esculenta |
| Colocasia fallax |
| Colocasia gigantea |
| Colocasia mannii |
| Colocasia menglaensis |
| Colocasia oresbia |
| Croatiella integrifolia |
| Cryptocoryne affinis |
| Cryptocoryne alba |
| Cryptocoryne albida |
| Cryptocoryne annamica |
| Cryptocoryne aponogetifolia |
| Cryptocoryne auriculata |
| Cryptocoryne bangkaensis |
| Cryptocoryne beckettii |
| Cryptocoryne bogneri |
| Cryptocoryne bullosa |
| Cryptocoryne ciliata |
| Cryptocoryne cognata |
| Cryptocoryne consobrina |
| Cryptocoryne cordata var. cordata |
| Cryptocoryne cordata var. diderici |
| Cryptocoryne cordata var. evae |
| Cryptocoryne cordata var. grabowskii |
| Cryptocoryne cordata var. zonata |
| Cryptocoryne coronata |
| Cryptocoryne crispatula var. crispatula |
| Cryptocoryne crispatula var. balansae |
| Cryptocoryne crispatula var. decus-mekongensis |
| Cryptocoryne crispatula var. flaccidifolia |
| Cryptocoryne crispatula var. planifolia |
| Cryptocoryne crispatula var. tonkinensis |
| Cryptocoryne crispatula var. yunnanensis |
| Cryptocoryne cruddasiana |
| Cryptocoryne decus-silvae |
| Cryptocoryne dewitii |
| Cryptocoryne edithiae |
| Cryptocoryne elliptica |
| Cryptocoryne ferruginea |
| Cryptocoryne fusca |
| Cryptocoryne griffithii |
| Cryptocoryne hudoroi |
| Cryptocoryne ideii |
| Cryptocoryne jacobsenii |
| Cryptocoryne keei |
| Cryptocoryne lingua |
| Cryptocoryne loeiensis |
| Cryptocoryne longicauda |
| Cryptocoryne mekongensis |
| Cryptocoryne minima |
| Cryptocoryne moehlmannii |
| Cryptocoryne nevillii |
| Cryptocoryne noritoi |
| Cryptocoryne nurii |
| Cryptocoryne pallidinervia |
| Cryptocoryne parva |
| Cryptocoryne pontederiifolia |
| Cryptocoryne pygmaea |
| Cryptocoryne retrospiralis |
| Cryptocoryne schulzei |
| Cryptocoryne scurrilis |
| Cryptocoryne sivadasanii |
| Cryptocoryne spiralis var. spiralis |
| Cryptocoryne spiralis var. cognatoides |
| Cryptocoryne striolata |
| Cryptocoryne tambraparaniana |
| Cryptocoryne thwaitesii |
| Cryptocoryne uenoi |
| Cryptocoryne undulata |
| Cryptocoryne usteriana |
| Cryptocoryne versteegii |
| Cryptocoryne vietnamensis |
| Cryptocoryne villosa |
| Cryptocoryne walkeri |
| Cryptocoryne wendtii |
| Cryptocoryne yujii |
| Cryptocoryne zaidiana |
| Cryptocoryne zukalii |
| Culcasia angolensis |
| Culcasia annetii |
| Culcasia bosii |
| Culcasia brevipetiolata |
| Culcasia caudata |
| Culcasia dinklagei |
| Culcasia ekongoloi |
| Culcasia falcifolia |
| Culcasia glandulosa |
| Culcasia insulana |
| Culcasia lanceolata |
| Culcasia liberica |
| Culcasia linearifolia |
| Culcasia loukandensis |
| Culcasia mannii |
| Culcasia obliquifolia |
| Culcasia orientalis |
| Culcasia panduriformis |
| Culcasia parviflora |
| Culcasia rotundifolia |
| Culcasia sanagensis |
| Culcasia scandens |
| Culcasia seretii |
| Culcasia simiarum |
| Culcasia striolata |
| Culcasia tenuifolia |
| Culcasia yangambiensis |
| Cyrtosperma beccarianum |
| Cyrtosperma bougainvillense |
| Cyrtosperma brassii |
| Cyrtosperma carrii |
| Cyrtosperma cuspidispathum |
| Cyrtosperma giganteum |
| Cyrtosperma gressittiorum |
| Cyrtosperma hambalii |
| Cyrtosperma johnstonii |
| Cyrtosperma kokodense |
| Cyrtosperma macrotum |
| Cyrtosperma merkusii |
| Dieffenbachia aglaonematifolia |
| Dieffenbachia antioquensis |
| Dieffenbachia aurantiaca |
| Dieffenbachia beachiana |
| Dieffenbachia bechiana |
| Dieffenbachia bowmannii |
| Dieffenbachia brittonii |
| Dieffenbachia burgeri |
| Dieffenbachia cannifolia |
| Dieffenbachia concinna |
| Dieffenbachia copensis |
| Dieffenbachia cordata |
| Dieffenbachia costata |
| Dieffenbachia crebripistillata |
| Dieffenbachia daguensis |
| Dieffenbachia davidsei |
| Dieffenbachia duidae |
| Dieffenbachia elegans |
| Dieffenbachia enderi |
| Dieffenbachia fortunensis |
| Dieffenbachia fosteri |
| Dieffenbachia fournieri |
| Dieffenbachia galdamesiae |
| Dieffenbachia gracilis |
| Dieffenbachia grayumiana |
| Dieffenbachia hammelii |
| Dieffenbachia herthae |
| Dieffenbachia horichii |
| Dieffenbachia humilis |
| Dieffenbachia imperialis |
| Dieffenbachia isthmia |
| Dieffenbachia killipii |
| Dieffenbachia lancifolia |
| Dieffenbachia leopoldii |
| Dieffenbachia longispatha |
| Dieffenbachia lutheri |
| Dieffenbachia macrophylla |
| Dieffenbachia meleagris |
| Dieffenbachia nitidipetiolata |
| Dieffenbachia obliqua |
| Dieffenbachia obscurinervia |
| Dieffenbachia oerstedii |
| Dieffenbachia olbia |
| Dieffenbachia paludicola |
| Dieffenbachia panamensis |
| Dieffenbachia parlatorei |
| Dieffenbachia parvifolia |
| Dieffenbachia pittieri |
| Dieffenbachia seguine |
| Dieffenbachia shuttleworthiana |
| Dieffenbachia standleyi |
| Dieffenbachia tonduzii |
| Dieffenbachia weberbaueri |
| Dieffenbachia weirii |
| Dieffenbachia wendlandii |
| Dieffenbachia williamsii |
| Dieffenbachia wurdackii |
| Dracontioides desciscens |
| Dracontioides salvianii |
| Dracontium amazonense |
| Dracontium angustispathum |
| Dracontium asperispathum |
| Dracontium asperum |
| Dracontium bogneri |
| Dracontium croatii |
| Dracontium dubium |
| Dracontium gigas |
| Dracontium grandispathum |
| Dracontium grayumianum |
| Dracontium guianense |
| Dracontium iquitense |
| Dracontium longipes |
| Dracontium margaretae |
| Dracontium nivosum |
| Dracontium peruvianum |
| Dracontium pittieri |
| Dracontium plowmanii |
| Dracontium polyphyllum |
| Dracontium prancei |
| Dracontium purdieanum |
| Dracontium soconuscum |
| Dracontium spruceanum |
| Dracontium ulei |
| Dracunculus canariensis |
| Dracunculus vulgaris |
| Eminium albertii |
| Eminium heterophyllum |
| Eminium intortum |
| Eminium jaegeri |
| Eminium koenenianum |
| Eminium lehmannii |
| Eminium rauwolffii var. rauwolffii |
| Eminium rauwolffii var. kotschyi |
| Eminium regelii |
| Eminium spiculatum |
| Epipremnum amplissimum |
| Epipremnum aureum |
| Epipremnum carolinense |
| Epipremnum ceramense |
| Epipremnum dahlii |
| Epipremnum falcifolium |
| Epipremnum giganteum |
| Epipremnum meeboldii |
| Epipremnum moluccanum |
| Epipremnum moszkowskii |
| Epipremnum nobile |
| Epipremnum obtusum |
| Epipremnum papuanum |
| Epipremnum pinnatum |
| Epipremnum silvaticum |
| Filarum manserichense |
| Furtadoa mixta |
| Furtadoa sumatrensis |
| Gearum brasiliense |
| Gonatopus angustus |
| Gonatopus boivinii |
| Gonatopus clavatus |
| Gonatopus marattioides |
| Gonatopus petiolulatus |
| Gorgonidium beckianum |
| Gorgonidium bulbostylum |
| Gorgonidium cardenasianum |
| Gorgonidium intermedium |
| Gorgonidium mirabile |
| Gorgonidium striatum |
| Gorgonidium vargasii |
| Gorgonidium vermicidum |
| Gymnostachys anceps |
| Hapaline appendiculata |
| Hapaline benthamiana |
| Hapaline brownii |
| Hapaline celatrix |
| Hapaline colaniae |
| Hapaline ellipticifolia |
| Hapaline kerrii |
| Hapaline locii |
| Helicodiceros muscivorus |
| Hestia longifolia |
| Heteropsis boliviana |
| Heteropsis croatii |
| Heteropsis duckeana |
| Heteropsis ecuadorensis |
| Heteropsis flexuosa var. flexuosa |
| Heteropsis flexuosa var. maguirei |
| Heteropsis linearis |
| Heteropsis longispathacea |
| Heteropsis macrophylla |
| Heteropsis melinonii |
| Heteropsis oblongifolia |
| Heteropsis peruviana |
| Heteropsis rigidifolia |
| Heteropsis robusta |
| Heteropsis salicifolia |
| Heteropsis spruceana |
| Heteropsis steyermarkii |
| Heteropsis tenuispadix |
| Holochlamys beccarii |
| Homalomena adiensis |
| Homalomena aeneifolia |
| Homalomena agens |
| Homalomena ardua |
| Homalomena argentea |
| Homalomena aromatica |
| Homalomena asmae |
| Homalomena asperifolia |
| Homalomena atroviridis |
| Homalomena atrox |
| Homalomena batoeensis |
| Homalomena bellula |
| Homalomena burkilliana |
| Homalomena clandestina |
| Homalomena cochinchinensis |
| Homalomena confusa |
| Homalomena consobrina |
| Homalomena cordata |
| Homalomena corneri |
| Homalomena crinipes |
| Homalomena cristata |
| Homalomena curtisii |
| Homalomena curvata |
| Homalomena davidiana |
| Homalomena debilicrista |
| Homalomena distans |
| Homalomena doctersii |
| Homalomena elegans |
| Homalomena elegantula |
| Homalomena erythropus subsp. erythropus |
| Homalomena erythropus subsp. allenii |
| Homalomena expedita |
| Homalomena gadutensis |
| Homalomena gaudichaudii |
| Homalomena giamensis |
| Homalomena gillii |
| Homalomena griffithii |
| Homalomena hainanensis |
| Homalomena hammelii |
| Homalomena hanneae |
| Homalomena hastata |
| Homalomena havilandii |
| Homalomena hendersonii |
| Homalomena hooglandii |
| Homalomena humilis |
| Homalomena impudica |
| Homalomena insignis |
| Homalomena jacobsiana |
| Homalomena josefii |
| Homalomena kalkmanii |
| Homalomena kelungensis |
| Homalomena kiahii |
| Homalomena korthalsii |
| Homalomena kualakohensis |
| Homalomena kvistii |
| Homalomena lancea |
| Homalomena lancifolia |
| Homalomena latifrons |
| Homalomena lauterbachii |
| Homalomena lindenii |
| Homalomena longipes |
| Homalomena magna |
| Homalomena major |
| Homalomena matangae |
| Homalomena megalophylla |
| Homalomena melanesica |
| Homalomena metallica |
| Homalomena minor |
| Homalomena minutissima |
| Homalomena moffleriana |
| Homalomena monandra |
| Homalomena montana |
| Homalomena nigrescens |
| Homalomena nutans |
| Homalomena obovata |
| Homalomena obscurifolia |
| Homalomena occulta |
| Homalomena ovalifolia |
| Homalomena ovata |
| Homalomena padangensis |
| Homalomena palawanensis |
| Homalomena peekelii |
| Homalomena peltata |
| Homalomena pendula |
| Homalomena philippinensis |
| Homalomena picturata |
| Homalomena pineodora |
| Homalomena pontederifolia |
| Homalomena producta |
| Homalomena pseudogeniculata |
| Homalomena pulleana |
| Homalomena punctulata |
| Homalomena pyrospatha |
| Homalomena robusta |
| Homalomena roezelii |
| Homalomena rostrata |
| Homalomena rubescens |
| Homalomena rusdii |
| Homalomena sarawakensis |
| Homalomena saxorum |
| Homalomena schlechteri |
| Homalomena scortechinii |
| Homalomena sengkenyang |
| Homalomena silvatica |
| Homalomena singaporensis |
| Homalomena soniae |
| Homalomena speariae |
| Homalomena steenisiana |
| Homalomena stollei |
| Homalomena striatieopetiolata |
| Homalomena subcordata |
| Homalomena symplocarpifolia |
| Homalomena tenuispadix |
| Homalomena terajaensis |
| Homalomena treubii |
| Homalomena truncata |
| Homalomena vagans |
| Homalomena vietnamensis |
| Homalomena vittifolia |
| Homalomena vivens |
| Homalomena wallichii |
| Homalomena wallisii |
| Homalomena wendlandii |
| Homalomena wongii |
| Homalomena zollingeri |
| Incarum pavonii |
| Jasarum steyermarkii |
| Lagenandra bogneri |
| Lagenandra dewitii |
| Lagenandra erosa |
| Lagenandra gomezii |
| Lagenandra jacobsenii |
| Lagenandra keralensis |
| Lagenandra koenigii |
| Lagenandra lancifolia |
| Lagenandra meeboldii |
| Lagenandra nairii |
| Lagenandra ovata |
| Lagenandra praetermissa |
| Lagenandra thwaitesii |
| Lagenandra toxicaria |
| Lagenandra undulata |
| Lasia concinna |
| Lasia spinosa |
| Lasimorpha senegalensis |
| Lemna aequinoctialis |
| Lemna disperma |
| Lemna gibba |
| Lemna japonica |
| Lemna minor |
| Lemna minuta |
| Lemna obscura |
| Lemna perpusilla |
| Lemna tenera |
| Lemna trisulca |
| Lemna turionifera |
| Lemna valdiviana |
| Lemna yungensis |
| Lorenzia umbrosa |
| Lysichiton americanus |
| Lysichiton camtschatcensis |
| Mangonia tweedieana |
| Mangonia uruguaya |
| Monstera acacoyaguensis |
| Monstera acuminata |
| Monstera adansonii var. adansonii |
| Monstera adansonii var. klotzschiana |
| Monstera adansonii var. laniata |
| Monstera amargalensis |
| Monstera aureopinnata |
| Monstera barrieri |
| Monstera buseyi |
| Monstera cenepensis |
| Monstera costaricensis |
| Monstera deliciosa |
| Monstera dissecta |
| Monstera dubia |
| Monstera epipremnoides |
| Monstera filamentosa |
| Monstera florescanoana |
| Monstera glaucescens |
| Monstera gracilis |
| Monstera kessleri |
| Monstera lechleriana |
| Monstera lentii |
| Monstera luteynii |
| Monstera maderaverde |
| Monstera membranacea |
| Monstera minima |
| Monstera molinae |
| Monstera obliqua |
| Monstera oreophila |
| Monstera pinnatipartita |
| Monstera pittieri |
| Monstera planadensis |
| Monstera praetermissa |
| Monstera punctulata |
| Monstera siltepecana |
| Monstera spruceana |
| Monstera standleyana |
| Monstera subpinnata |
| Monstera tenuis |
| Monstera tuberculata var. tuberculata |
| Monstera tuberculata var. brevinoda |
| Monstera vasquezii |
| Monstera xanthospatha |
| Montrichardia arborescens |
| Montrichardia linifera |
| Nephthytis afzelii var. afzelii |
| Nephthytis afzelii var. graboensis |
| Nephthytis bintuluensis |
| Nephthytis hallaei |
| Nephthytis mayombensis |
| Nephthytis poissonii var. poissonii |
| Nephthytis poissonii var. constricta |
| Nephthytis swainei |
| Ooia grabowskii |
| Ooia kinabaluensis |
| Orontium aquaticum |
| Pedicellarum paiei |
| Peltandra sagittifolia |
| Peltandra virginica |
| Philodendron acreanum |
| Philodendron acuminatissimum |
| Philodendron acutifolium |
| Philodendron adamantinum |
| Philodendron adhatodifolium |
| Philodendron advena |
| Philodendron aemulum |
| Philodendron alatum |
| Philodendron albisuccus |
| Philodendron alliodorum |
| Philodendron alternans |
| Philodendron alticola |
| Philodendron altomacaense |
| Philodendron amargalense |
| Philodendron ampamii |
| Philodendron amplisinum |
| Philodendron ampullaceum |
| Philodendron anaadu |
| Philodendron ancuashii |
| Philodendron angustialatum |
| Philodendron angustilobum |
| Philodendron angustisectum |
| Philodendron anisotomum |
| Philodendron annulatum |
| Philodendron antonioanum |
| Philodendron appendiculatum |
| Philodendron applanatum |
| Philodendron appunii |
| Philodendron aristeguietae |
| Philodendron aromaticum |
| Philodendron asplundii |
| Philodendron atabapoense |
| Philodendron aurantiifolium subsp. aurantiifolium |
| Philodendron aurantiifolium subsp. calderense |
| Philodendron aurantispadix |
| Philodendron aureimarginatum |
| Philodendron auriculatum |
| Philodendron auritum |
| Philodendron auyantepuiense |
| Philodendron avenium |
| Philodendron azulitense |
| Philodendron bahiense |
| Philodendron bakeri |
| Philodendron balaoanum |
| Philodendron barbourii |
| Philodendron barrosoanum |
| Philodendron basii |
| Philodendron basivaginatum |
| Philodendron baudoense |
| Philodendron beniteziae |
| Philodendron bernardopazii |
| Philodendron billietiae |
| Philodendron bipennifolium |
| Philodendron bipinnatifidum |
| Philodendron biribiriense |
| Philodendron blanchetianum |
| Philodendron bogotense |
| Philodendron borgesii |
| Philodendron brandii |
| Philodendron brandtianum |
| Philodendron brasiliense |
| Philodendron breedlovei |
| Philodendron brenesii |
| Philodendron brent-berlinii |
| Philodendron brevispathum |
| Philodendron brewsterense |
| Philodendron brunneicaule |
| Philodendron buchtienii |
| Philodendron buntingianum |
| Philodendron burgeri |
| Philodendron burle-marxii |
| Philodendron calatheifolium |
| Philodendron callosum subsp. callosum |
| Philodendron callosum subsp. ptarianum |
| Philodendron campii |
| Philodendron camposportoanum |
| Philodendron canaimae |
| Philodendron canicaule |
| Philodendron cardonii |
| Philodendron cardosoi |
| Philodendron carinatum |
| Philodendron cataniapoense |
| Philodendron caudatum |
| Philodendron chimantae |
| Philodendron chimboanum |
| Philodendron chinchamayense |
| Philodendron chiriquense |
| Philodendron chirripoense |
| Philodendron chrysocarpum |
| Philodendron cipoense |
| Philodendron clarkei |
| Philodendron clewellii |
| Philodendron colombianum |
| Philodendron coloradense |
| Philodendron condorcanquense |
| Philodendron conforme |
| Philodendron consanguineum |
| Philodendron consobrinum |
| Philodendron copense |
| Philodendron corcovadense |
| Philodendron cordatum |
| Philodendron coriaceum |
| Philodendron correae |
| Philodendron cotapatense |
| Philodendron cotobrusense |
| Philodendron cotonense |
| Philodendron craspedodromum |
| Philodendron crassinervium |
| Philodendron crassispathum |
| Philodendron crassum |
| Philodendron cremersii |
| Philodendron cretosum |
| Philodendron croatii |
| Philodendron cruentospathum |
| Philodendron cruentum |
| Philodendron cuneatum |
| Philodendron curvilobum |
| Philodendron daniellii |
| Philodendron danteanum |
| Philodendron dardanianum |
| Philodendron davidsei |
| Philodendron davidsonii subsp. davidsonii |
| Philodendron davidsonii subsp. bocatoranum |
| Philodendron deflexum |
| Philodendron delascioi |
| Philodendron delinksii |
| Philodendron deltoideum |
| Philodendron densivenium |
| Philodendron devansayanum |
| Philodendron devianum |
| Philodendron dioscoreoides |
| Philodendron discretivenium |
| Philodendron distantilobum |
| Philodendron divaricatum |
| Philodendron dodsonii |
| Philodendron dolichophyllum |
| Philodendron dominicalense |
| Philodendron dressleri |
| Philodendron dryanderae |
| Philodendron duckei |
| Philodendron dunstervilleorum |
| Philodendron dussii |
| Philodendron dwyeri |
| Philodendron dyscarpium var. dyscarpium |
| Philodendron dyscarpium var. ventuarianum |
| Philodendron eburneum |
| Philodendron ecordatum |
| Philodendron edenudatum |
| Philodendron edmundoi |
| Philodendron effusilobum |
| Philodendron elaphoglossoides |
| Philodendron elegans |
| Philodendron elegantulum |
| Philodendron englerianum subsp. englerianum |
| Philodendron englerianum subsp. duidae |
| Philodendron ensifolium subsp. ensifolium |
| Philodendron ensifolium subsp. campanense |
| Philodendron ensifolium subsp. colonense |
| Philodendron ernestii |
| Philodendron erubescens |
| Philodendron escuintlense |
| Philodendron exile |
| Philodendron eximium |
| Philodendron fendleri |
| Philodendron ferrugineum |
| Philodendron fibrillosum |
| Philodendron fibrosum |
| Philodendron findens |
| Philodendron flumineum |
| Philodendron follii |
| Philodendron folsomii |
| Philodendron fortunense |
| Philodendron fragile |
| Philodendron fragrantissimum |
| Philodendron fraternum |
| Philodendron furcatum |
| Philodendron giganteum |
| Philodendron gigas |
| Philodendron glanduliferum subsp. glanduliferum |
| Philodendron glanduliferum subsp. camiloanum |
| Philodendron glaziovii |
| Philodendron gloriosum |
| Philodendron goeldii |
| Philodendron gonzalezii |
| Philodendron grandifolium |
| Philodendron grandipes |
| Philodendron granulare |
| Philodendron graveolens |
| Philodendron grayumii |
| Philodendron grazielae |
| Philodendron grenandii |
| Philodendron guaiquinimae |
| Philodendron gualeanum |
| Philodendron guianense |
| Philodendron guttiferum |
| Philodendron hammelii |
| Philodendron hastatum |
| Philodendron hatschbachii |
| Philodendron hebetatum |
| Philodendron hederaceum var. hederaceum |
| Philodendron hederaceum var. kirkbridei |
| Philodendron hederaceum var. oxycardium |
| Philodendron heleniae subsp. heleniae |
| Philodendron heleniae subsp. amazonense |
| Philodendron henry-pittieri |
| Philodendron herbaceum |
| Philodendron herthae |
| Philodendron heterocraspedon |
| Philodendron heterophyllum |
| Philodendron heteropleurum |
| Philodendron holstii |
| Philodendron hooveri |
| Philodendron hopkinsianum |
| Philodendron houlletianum |
| Philodendron huanucense |
| Philodendron huashikatii |
| Philodendron huaynacapacense |
| Philodendron humile |
| Philodendron hylaeae |
| Philodendron ichthyoderma |
| Philodendron immixtum |
| Philodendron inaequilaterum |
| Philodendron inconcinnum |
| Philodendron inops |
| Philodendron insigne |
| Philodendron jacquinii |
| Philodendron jefense |
| Philodendron jodavisianum |
| Philodendron jonkerorum |
| Philodendron juninense |
| Philodendron kautskyi |
| Philodendron killipii |
| Philodendron knappiae |
| Philodendron krauseanum |
| Philodendron kroemeri |
| Philodendron krugii |
| Philodendron lacerum |
| Philodendron laticiferum |
| Philodendron latifolium |
| Philodendron lazorii |
| Philodendron leal-costae |
| Philodendron lechlerianum |
| Philodendron lehmannii |
| Philodendron lemae |
| Philodendron lentii |
| Philodendron leucanthum |
| Philodendron leyvae |
| Philodendron liesneri |
| Philodendron ligulatum var. ligulatum |
| Philodendron ligulatum var. heraclioanum |
| Philodendron ligulatum var. ovatum |
| Philodendron lindenianum |
| Philodendron lindenii |
| Philodendron linguifolium |
| Philodendron lingulatum |
| Philodendron linnaei var. linnaei |
| Philodendron linnaei var. rionegrense |
| Philodendron llanense |
| Philodendron loefgrenii |
| Philodendron longilaminatum |
| Philodendron longilobatum |
| Philodendron longipedunculatum |
| Philodendron longipes |
| Philodendron longirrhizum |
| Philodendron longistilum |
| Philodendron lundii |
| Philodendron lupinum |
| Philodendron macroglossum |
| Philodendron macropodum |
| Philodendron maculatum |
| Philodendron madronense |
| Philodendron maguirei |
| Philodendron malesevichiae |
| Philodendron mamei |
| Philodendron marahuacae |
| Philodendron maroae |
| Philodendron martianum |
| Philodendron martini |
| Philodendron mathewsii |
| Philodendron mawarinumae |
| Philodendron maximum |
| Philodendron mayoi |
| Philodendron mcphersonii |
| Philodendron megalophyllum |
| Philodendron melanochrysum |
| Philodendron melinonii |
| Philodendron mello-barretoanum |
| Philodendron membranaceum |
| Philodendron merenbergense |
| Philodendron meridense |
| Philodendron mesae |
| Philodendron mexicanum |
| Philodendron micranthum |
| Philodendron microstictum |
| Philodendron millerianum |
| Philodendron minarum |
| Philodendron misahualliense |
| Philodendron missionum |
| Philodendron modestum |
| Philodendron monsalveae |
| Philodendron montanum |
| Philodendron moonenii |
| Philodendron morii |
| Philodendron multinervum |
| Philodendron multispadiceum |
| Philodendron muricatum |
| Philodendron musifolium |
| Philodendron myrmecophilum |
| Philodendron nadruzianum |
| Philodendron nanegalense |
| Philodendron narinoense |
| Philodendron nebulense |
| Philodendron ninoanum |
| Philodendron niqueanum |
| Philodendron nullinervium |
| Philodendron oblanceolatum |
| Philodendron obliquifolium |
| Philodendron oblongum |
| Philodendron obtusilobum |
| Philodendron ochrostemon |
| Philodendron oligospermum |
| Philodendron opacum |
| Philodendron orionis |
| Philodendron ornatum |
| Philodendron pachycaule |
| Philodendron pachyphyllum |
| Philodendron palaciosii |
| Philodendron paludicola |
| Philodendron panamense |
| Philodendron panduriforme var. panduriforme |
| Philodendron  panduriforme var. reichenbachianum |
| Philodendron parvilobum |
| Philodendron pastazanum |
| Philodendron patriciae |
| Philodendron paucinervium |
| Philodendron paxianum |
| Philodendron pedatum |
| Philodendron pedunculum |
| Philodendron peperomioides |
| Philodendron peraiense |
| Philodendron perplexum |
| Philodendron phlebodes var. phlebodes |
| Philodendron phlebodes var. kermesinum |
| Philodendron pimichinese |
| Philodendron pinnatifidum |
| Philodendron pinnatilobum |
| Philodendron pipolyi |
| Philodendron pirrense |
| Philodendron placidum |
| Philodendron planadense |
| Philodendron platypetiolatum |
| Philodendron platypodum |
| Philodendron pogonocaule |
| Philodendron polliciforme |
| Philodendron popenoei |
| Philodendron populneum |
| Philodendron prominulinervium |
| Philodendron propinquum |
| Philodendron pseudauriculatum |
| Philodendron pseudoundulatum |
| Philodendron pteropus |
| Philodendron pterotum |
| Philodendron puhuangii |
| Philodendron pulchellum |
| Philodendron pulchrum |
| Philodendron purpureoviride |
| Philodendron purulhense |
| Philodendron pusillum |
| Philodendron quinquelobum |
| Philodendron quinquenervium |
| Philodendron quitense |
| Philodendron radiatum var. radiatum |
| Philodendron radiatum var. pseudoradiatum |
| Philodendron rayanum |
| Philodendron recurvifolium |
| Philodendron remifolium subsp. remifolium |
| Philodendron remifolium subsp. sabulosum |
| Philodendron renauxii |
| Philodendron reticulatum |
| Philodendron rhizomatosum |
| Philodendron rhodoaxis subsp. rhodoaxis |
| Philodendron rhodoaxis subsp. lewisii |
| Philodendron rhodospathiphyllum |
| Philodendron ricardoi |
| Philodendron rigidifolium subsp. rigidifolium |
| Philodendron rigidifolium subsp. sanctae-ritae |
| Philodendron rimachii |
| Philodendron riparium |
| Philodendron robustum |
| Philodendron rodrigueziae |
| Philodendron roezlii |
| Philodendron rojasianum |
| Philodendron romeroi |
| Philodendron roraimae var. roraimae |
| Philodendron roraimae subsp. aracamuniense |
| Philodendron roseocataphyllum |
| Philodendron roseopetiolatum |
| Philodendron roseospathum var. roseospathum |
| Philodendron roseospathum var. angustilaminatum |
| Philodendron rothschuhianum |
| Philodendron rubrocinctum |
| Philodendron rubromaculatum |
| Philodendron rudgeanum |
| Philodendron rugosum |
| Philodendron ruizii |
| Philodendron ruthianum |
| Philodendron sagittifolium |
| Philodendron samayense |
| Philodendron santodominguense |
| Philodendron saxicola |
| Philodendron scalarinerve |
| Philodendron scherberichii |
| Philodendron schottianum |
| Philodendron schottii subsp. schottii |
| Philodendron schottii subsp. talamancae |
| Philodendron scitulum |
| Philodendron scottmorianum |
| Philodendron seguine subsp. seguine |
| Philodendron seguine subsp. lingua-bovis |
| Philodendron senatocarpium |
| Philodendron serpens |
| Philodendron silverstonei |
| Philodendron simmondsii |
| Philodendron simonianum |
| Philodendron simsii |
| Philodendron simulans |
| Philodendron smithii |
| Philodendron solimoesense |
| Philodendron sonderianum |
| Philodendron sousae |
| Philodendron sparreorum |
| Philodendron speciosum |
| Philodendron sphalerum |
| Philodendron spiritus-sancti |
| Philodendron splitgerberi |
| Philodendron spruceanum |
| Philodendron squamicaule |
| Philodendron squamiferum |
| Philodendron squamipetiolatum |
| Philodendron standleyi |
| Philodendron stenolobum |
| Philodendron stenophyllum |
| Philodendron steyermarkii |
| Philodendron straminicaule |
| Philodendron striatum |
| Philodendron strictum |
| Philodendron suberosum |
| Philodendron subhastatum |
| Philodendron subincisum |
| Philodendron sucrense |
| Philodendron sulcatum |
| Philodendron sulcicaule |
| Philodendron surinamense |
| Philodendron swartiae |
| Philodendron tachirense |
| Philodendron tarmense |
| Philodendron tatei subsp. tatei |
| Philodendron tatei subsp. melanochlorum |
| Philodendron tenue |
| Philodendron tenuipes |
| Philodendron tenuispadix |
| Philodendron teretipes |
| Philodendron thalassicum |
| Philodendron thaliifolium |
| Philodendron tortum |
| Philodendron toshibae |
| Philodendron traunii |
| Philodendron triangulare |
| Philodendron tricostatum |
| Philodendron tripartitum |
| Philodendron triplum |
| Philodendron trojitense |
| Philodendron trujilloi |
| Philodendron tuerckheimii |
| Philodendron tweedieanum |
| Philodendron tysonii |
| Philodendron ubigantupense |
| Philodendron uleanum |
| Philodendron uliginosum |
| Philodendron undulatum |
| Philodendron urraoense |
| Philodendron ushanum |
| Philodendron utleyanum |
| Philodendron validinervium |
| Philodendron vargealtense |
| Philodendron variifolium |
| Philodendron venezuelense |
| Philodendron venosum |
| Philodendron ventricosum |
| Philodendron venulosum |
| Philodendron venustifoliatum |
| Philodendron venustum |
| Philodendron verapazense |
| Philodendron verrucapetiolum |
| Philodendron verrucosum |
| Philodendron victoriae |
| Philodendron vinaceum |
| Philodendron viride |
| Philodendron wadedavisii |
| Philodendron wallisii |
| Philodendron warszewiczii |
| Philodendron weberbaueri |
| Philodendron wendlandii |
| Philodendron werkhoveniae |
| Philodendron wilburii var. wilburii |
| Philodendron wilburii var. longipedunculatum |
| Philodendron williamsii |
| Philodendron wittianum |
| Philodendron woronowii |
| Philodendron wullschlaegelii |
| Philodendron wurdackii |
| Philodendron xanadu |
| Philodendron yavitense |
| Philodendron yutajense |
| Philodendron zhuanum |
| Philonotion americanum |
| Philonotion bolivaranum |
| Philonotion spruceanum |
| Phymatarum borneense |
| Pichinia disticha |
| Pinellia cordata |
| Pinellia fujianensis |
| Pinellia integrifolia |
| Pinellia pedatisecta |
| Pinellia peltata |
| Pinellia polyphylla |
| Pinellia ternata |
| Pinellia tripartita |
| Pinellia yaoluopingensis |
| Piptospatha burbidgei |
| Piptospatha elongata |
| Piptospatha impolita |
| Piptospatha insignis |
| Piptospatha manduensis |
| Piptospatha marginata |
| Piptospatha perakensis |
| Piptospatha remiformis |
| Piptospatha repens |
| Piptospatha ridleyi |
| Piptospatha truncata |
| Piptospatha viridistigma |
| Pistia stratiotes |
| Podolasia stipitata |
| Pothoidium lobbianum |
| Pothos armatus |
| Pothos atropurpurascens |
| Pothos barberianus |
| Pothos beccarianus |
| Pothos brassii |
| Pothos brevistylus |
| Pothos brevivaginatus |
| Pothos chinensis |
| Pothos clavatus |
| Pothos crassipedunculatus |
| Pothos curtisii |
| Pothos cuspidatus |
| Pothos cylindricus |
| Pothos dolichophyllus |
| Pothos dzui |
| Pothos englerianus |
| Pothos falcifolius |
| Pothos gigantipes |
| Pothos gracillimus |
| Pothos grandis |
| Pothos hellwigii |
| Pothos hookeri |
| Pothos inaequilaterus |
| Pothos insignis |
| Pothos junghuhnii |
| Pothos keralensis |
| Pothos kerrii |
| Pothos kingii |
| Pothos lancifolius |
| Pothos laurifolius |
| Pothos leptostachyus |
| Pothos longipes |
| Pothos longivaginatus |
| Pothos luzonensis |
| Pothos macrocephalus |
| Pothos mirabilis |
| Pothos motleyanus |
| Pothos oliganthus |
| Pothos ovatifolius |
| Pothos oxyphyllus |
| Pothos papuanus |
| Pothos parvispadix |
| Pothos philippinensis |
| Pothos pilulifer |
| Pothos polystachyus |
| Pothos remotiflorus |
| Pothos repens |
| Pothos roxburghii |
| Pothos salicifolius |
| Pothos scandens |
| Pothos tener |
| Pothos thomsonianus |
| Pothos touranensis |
| Pothos versteegii |
| Pothos volans |
| Pothos zippelii |
| Protarum sechellarum |
| Pseudohydrosme buettneri |
| Pseudohydrosme gabunensis |
| Pycnospatha arietina |
| Pycnospatha palmata |
| Remusatia hookeriana |
| Remusatia pumila |
| Remusatia vivipara |
| Remusatia yunnanensis |
| Rhaphidophora acuminata |
| Rhaphidophora africana |
| Rhaphidophora angustata |
| Rhaphidophora araea |
| Rhaphidophora australasica |
| Rhaphidophora balgooyi |
| Rhaphidophora banosensis |
| Rhaphidophora beccarii |
| Rhaphidophora bonii |
| Rhaphidophora brevispathacea |
| Rhaphidophora burkilliana |
| Rhaphidophora calophylla |
| Rhaphidophora chevalieri |
| Rhaphidophora conica |
| Rhaphidophora conocephala |
| Rhaphidophora corneri |
| Rhaphidophora crassicaulis |
| Rhaphidophora crassifolia |
| Rhaphidophora cravenschoddeana |
| Rhaphidophora cretosa |
| Rhaphidophora cryptantha |
| Rhaphidophora cylindrosperma |
| Rhaphidophora dahlii |
| Rhaphidophora decursiva |
| Rhaphidophora discolor |
| Rhaphidophora dulongensis |
| Rhaphidophora elliptica |
| Rhaphidophora elliptifolia |
| Rhaphidophora elmeri |
| Rhaphidophora falcata |
| Rhaphidophora floresensis |
| Rhaphidophora foraminifera |
| Rhaphidophora formosana |
| Rhaphidophora fortis |
| Rhaphidophora geniculata |
| Rhaphidophora glauca |
| Rhaphidophora gorokensis |
| Rhaphidophora guamensis |
| Rhaphidophora hayi |
| Rhaphidophora hongkongensis |
| Rhaphidophora hookeri |
| Rhaphidophora intonsa |
| Rhaphidophora intrusa |
| Rhaphidophora jubata |
| Rhaphidophora kokodensis |
| Rhaphidophora koordersii |
| Rhaphidophora korthalsii |
| Rhaphidophora laichauensis |
| Rhaphidophora lancifolia |
| Rhaphidophora latevaginata |
| Rhaphidophora liukiuensis |
| Rhaphidophora lobbii |
| Rhaphidophora luchunensis |
| Rhaphidophora maingayi |
| Rhaphidophora megaphylla |
| Rhaphidophora megasperma |
| Rhaphidophora megastigma |
| Rhaphidophora microspadix |
| Rhaphidophora mima |
| Rhaphidophora minor |
| Rhaphidophora moluccensis |
| Rhaphidophora montana |
| Rhaphidophora monticola |
| Rhaphidophora neoguineensis |
| Rhaphidophora nicolsonii |
| Rhaphidophora okapensis |
| Rhaphidophora oligosperma |
| Rhaphidophora ovoidea |
| Rhaphidophora pachyphylla |
| Rhaphidophora parvifolia |
| Rhaphidophora peepla |
| Rhaphidophora peeploides |
| Rhaphidophora perkinsiae |
| Rhaphidophora pertusa |
| Rhaphidophora petrieana |
| Rhaphidophora philippinensis |
| Rhaphidophora pilosa |
| Rhaphidophora puberula |
| Rhaphidophora sabit |
| Rhaphidophora sarasinorum |
| Rhaphidophora schlechteri |
| Rhaphidophora schottii |
| Rhaphidophora spathacea |
| Rhaphidophora spuria |
| Rhaphidophora stenophylla |
| Rhaphidophora stolleana |
| Rhaphidophora sulcata |
| Rhaphidophora sylvestris |
| Rhaphidophora talamauana |
| Rhaphidophora tenuis |
| Rhaphidophora ternatensis |
| Rhaphidophora tetrasperma |
| Rhaphidophora teysmanniana |
| Rhaphidophora todayensis |
| Rhaphidophora tonkinensis |
| Rhaphidophora typha |
| Rhaphidophora ustulata |
| Rhaphidophora versteegii |
| Rhaphidophora waria |
| Rhodospatha acosta-solisii |
| Rhodospatha arborescens |
| Rhodospatha badilloi |
| Rhodospatha bolivarana |
| Rhodospatha boliviensis |
| Rhodospatha brachypoda |
| Rhodospatha brent-berlinii |
| Rhodospatha cardonae |
| Rhodospatha densinervia |
| Rhodospatha dissidens |
| Rhodospatha falconensis |
| Rhodospatha forgetii |
| Rhodospatha guasareensis |
| Rhodospatha herrerae |
| Rhodospatha katipas |
| Rhodospatha kraenzlinii |
| Rhodospatha latifolia |
| Rhodospatha monsalveae |
| Rhodospatha moritziana |
| Rhodospatha mukuntakia |
| Rhodospatha oblongata |
| Rhodospatha pellucida |
| Rhodospatha perezii |
| Rhodospatha piushaduka |
| Rhodospatha robusta |
| Rhodospatha statutii |
| Rhodospatha steyermarkii |
| Rhodospatha venosa |
| Rhodospatha wendlandii |
| Sauromatum brevipes |
| Sauromatum brevipilosum |
| Sauromatum diversifolium |
| Sauromatum gaoligongense |
| Sauromatum giganteum |
| Sauromatum hirsutum |
| Sauromatum horsfieldii |
| Sauromatum tentaculatum |
| Sauromatum venosum |
| Scaphispatha gracilis |
| Scaphispatha robusta |
| Schismatoglottis acutifolia |
| Schismatoglottis adoceta |
| Schismatoglottis ahmadii |
| Schismatoglottis ardenii |
| Schismatoglottis asperata |
| Schismatoglottis barbata |
| Schismatoglottis bauensis |
| Schismatoglottis bifasciata |
| Schismatoglottis bogneri |
| Schismatoglottis brevicuspis |
| Schismatoglottis calyptrata |
| Schismatoglottis canaliculata |
| Schismatoglottis ciliata |
| Schismatoglottis clarae |
| Schismatoglottis clausula |
| Schismatoglottis clemensiorum |
| Schismatoglottis confinis |
| Schismatoglottis conoidea |
| Schismatoglottis convolvula |
| Schismatoglottis corneri |
| Schismatoglottis crinitissima |
| Schismatoglottis cyria |
| Schismatoglottis decipiens |
| Schismatoglottis dilecta |
| Schismatoglottis dulosa |
| Schismatoglottis ecaudata |
| Schismatoglottis edanoi |
| Schismatoglottis elegans |
| Schismatoglottis erecta |
| Schismatoglottis eximia |
| Schismatoglottis eymae |
| Schismatoglottis ferruginea |
| Schismatoglottis gamoandra |
| Schismatoglottis gillianiae |
| Schismatoglottis glauca |
| Schismatoglottis grabowskii |
| Schismatoglottis hainanensis |
| Schismatoglottis harmandii |
| Schismatoglottis hayana |
| Schismatoglottis hayi |
| Schismatoglottis hottae |
| Schismatoglottis ifugaoensis |
| Schismatoglottis inculta |
| Schismatoglottis jelandii |
| Schismatoglottis jepomii |
| Schismatoglottis jitinae |
| Schismatoglottis josefii |
| Schismatoglottis kurzii |
| Schismatoglottis lancifolia |
| Schismatoglottis latevaginata |
| Schismatoglottis linae |
| Schismatoglottis lingua |
| Schismatoglottis longispatha |
| Schismatoglottis luzonensis |
| Schismatoglottis maelii |
| Schismatoglottis matangensis |
| Schismatoglottis mayoana |
| Schismatoglottis merrillii |
| Schismatoglottis mindanaoana |
| Schismatoglottis mira |
| Schismatoglottis modesta |
| Schismatoglottis monoplacenta |
| Schismatoglottis moodii |
| Schismatoglottis motleyana |
| Schismatoglottis multiflora |
| Schismatoglottis multinervia |
| Schismatoglottis nervosa |
| Schismatoglottis niahensis |
| Schismatoglottis nicolsonii |
| Schismatoglottis patentinervia |
| Schismatoglottis pectinervia |
| Schismatoglottis penangensis |
| Schismatoglottis petri |
| Schismatoglottis platystigma |
| Schismatoglottis plurivenia |
| Schismatoglottis puberulipes |
| Schismatoglottis pudenda |
| Schismatoglottis pumila |
| Schismatoglottis pusilla |
| Schismatoglottis pyrrhias |
| Schismatoglottis retinervia |
| Schismatoglottis roseospatha |
| Schismatoglottis samarensis |
| Schismatoglottis sarikeensis |
| Schismatoglottis schottii |
| Schismatoglottis scortechinii |
| Schismatoglottis sejuncta |
| Schismatoglottis silamensis |
| Schismatoglottis simonii |
| Schismatoglottis subundulata |
| Schismatoglottis tahubangensis |
| Schismatoglottis tecturata |
| Schismatoglottis tessellata |
| Schismatoglottis thelephora |
| Schismatoglottis trifasciata |
| Schismatoglottis trivittata |
| Schismatoglottis trusmadiensis |
| Schismatoglottis turbata |
| Schismatoglottis ulusarikeiensis |
| Schismatoglottis unifolia |
| Schismatoglottis venusta |
| Schismatoglottis viridissima |
| Schismatoglottis wahaiana |
| Schismatoglottis wallichii |
| Schismatoglottis warburgiana |
| Schismatoglottis wongii |
| Schismatoglottis zonata |
| Schottariella mirifica |
| Scindapsus alpinus |
| Scindapsus altissimus |
| Scindapsus beccarii |
| Scindapsus carolinensis |
| Scindapsus coriaceus |
| Scindapsus crassipes |
| Scindapsus curranii |
| Scindapsus cuscuaria |
| Scindapsus cuscuarioides |
| Scindapsus falcifolius |
| Scindapsus geniculatus |
| Scindapsus glaucescens |
| Scindapsus grandifolius |
| Scindapsus hederaceus |
| Scindapsus javanicus |
| Scindapsus latifolius |
| Scindapsus longipes |
| Scindapsus longistipitatus |
| Scindapsus lucens |
| Scindapsus maclurei |
| Scindapsus mamilliferus |
| Scindapsus marantifolius |
| Scindapsus officinalis |
| Scindapsus perakensis |
| Scindapsus pictus |
| Scindapsus roseus |
| Scindapsus rupestris |
| Scindapsus salomoniensis |
| Scindapsus schlechteri |
| Scindapsus scortechinii |
| Scindapsus splendidus |
| Scindapsus subcordatus |
| Scindapsus suffruticosus |
| Scindapsus sumatranus |
| Scindapsus treubii |
| Spathantheum fallax |
| Spathantheum orbignyanum |
| Spathicarpa gardneri |
| Spathicarpa hastifolia |
| Spathicarpa lanceolata |
| Spathiphyllum atrovirens |
| Spathiphyllum barbourii |
| Spathiphyllum bariense |
| Spathiphyllum blandum |
| Spathiphyllum brent-berlinii |
| Spathiphyllum brevirostre |
| Spathiphyllum buntingianum |
| Spathiphyllum cannifolium |
| Spathiphyllum cochlearispathum |
| Spathiphyllum commutatum |
| Spathiphyllum cuspidatum |
| Spathiphyllum diazii |
| Spathiphyllum dressleri |
| Spathiphyllum floribundum |
| Spathiphyllum friedrichsthalii |
| Spathiphyllum fulvovirens |
| Spathiphyllum gardneri |
| Spathiphyllum gracile |
| Spathiphyllum grandifolium |
| Spathiphyllum grazielae |
| Spathiphyllum humboldtii |
| Spathiphyllum jejunum |
| Spathiphyllum juninense |
| Spathiphyllum kalbreyeri |
| Spathiphyllum kochii |
| Spathiphyllum laeve |
| Spathiphyllum lanceifolium |
| Spathiphyllum lechlerianum |
| Spathiphyllum maguirei |
| Spathiphyllum matudae |
| Spathiphyllum mawarinumae |
| Spathiphyllum minor |
| Spathiphyllum monachinoi var. monachinoi |
| Spathiphyllum monachinoi var. perangustum |
| Spathiphyllum montanum |
| Spathiphyllum neblinae |
| Spathiphyllum ortgiesii |
| Spathiphyllum patinii |
| Spathiphyllum patulinervum |
| Spathiphyllum perezii |
| Spathiphyllum phryniifolium |
| Spathiphyllum pygmaeum |
| Spathiphyllum quindiuense |
| Spathiphyllum schlechteri |
| Spathiphyllum schomburgkii |
| Spathiphyllum silvicola |
| Spathiphyllum solomonense |
| Spathiphyllum tenerum |
| Spathiphyllum uspanapaensis |
| Spathiphyllum wallisii |
| Spathiphyllum wendlandii |
| Spirodela oligorrhiza |
| Spirodela polyrhiza |
| Spirodela punctata |
| Spirodela sichuanensis |
| Stenospermation adsimile |
| Stenospermation ammiticum |
| Stenospermation amomifolium |
| Stenospermation ancuashii |
| Stenospermation andreanum |
| Stenospermation angosturense |
| Stenospermation angustifolium |
| Stenospermation arborescens |
| Stenospermation archeri |
| Stenospermation benavidesae |
| Stenospermation brachypodum |
| Stenospermation crassifolium |
| Stenospermation densiovulatum |
| Stenospermation dictyoneurum |
| Stenospermation ellipticum |
| Stenospermation escobariae |
| Stenospermation flavescens |
| Stenospermation flavum |
| Stenospermation gentryi |
| Stenospermation glaucophyllum |
| Stenospermation gracile |
| Stenospermation hilligii |
| Stenospermation interruptum |
| Stenospermation laevis |
| Stenospermation latifolium |
| Stenospermation longifolium |
| Stenospermation longipetiolatum |
| Stenospermation longispadix |
| Stenospermation maguirei |
| Stenospermation majus |
| Stenospermation marantifolium |
| Stenospermation mathewsii var. mathewsii |
| Stenospermation mathewsii var. stipitatum |
| Stenospermation monsalvae |
| Stenospermation multiovulatum |
| Stenospermation nebulense |
| Stenospermation olgae |
| Stenospermation parvum |
| Stenospermation peripense |
| Stenospermation pittieri |
| Stenospermation popayanense |
| Stenospermation pteropus |
| Stenospermation robustum |
| Stenospermation rusbyi |
| Stenospermation sessile |
| Stenospermation spruceanum |
| Stenospermation subellipticum |
| Stenospermation ulei |
| Stenospermation velutinum |
| Stenospermation wallisii |
| Stenospermation zeacarpium |
| Steudnera assamica |
| Steudnera capitellata |
| Steudnera colocasiifolia |
| Steudnera colocasioides |
| Steudnera discolor |
| Steudnera gagei |
| Steudnera griffithii |
| Steudnera henryana |
| Steudnera kerrii |
| Stylochaeton angolense |
| Stylochaeton bogneri |
| Stylochaeton borumense |
| Stylochaeton crassispathum |
| Stylochaeton cuculliferum |
| Stylochaeton euryphyllum |
| Stylochaeton grande |
| Stylochaeton hypogeum |
| Stylochaeton kornasii |
| Stylochaeton lancifolium |
| Stylochaeton malaissei |
| Stylochaeton milneanum |
| Stylochaeton natalense subsp. natalense |
| Stylochaeton natalense subsp. maximum |
| Stylochaeton natalense subsp. obliquinerve |
| Stylochaeton oligocarpum |
| Stylochaeton pilosum |
| Stylochaeton puberulum |
| Stylochaeton salaamicum |
| Stylochaeton shabaense |
| Stylochaeton tortispathum |
| Stylochaeton zenkeri |
| Symplocarpus egorovii |
| Symplocarpus foetidus |
| Symplocarpus nabekuraensis |
| Symplocarpus nipponicus |
| Symplocarpus renifolius |
| Synandrospadix vermitoxicus |
| Syngonium angustatum |
| Syngonium armigerum |
| Syngonium atrovirens |
| Syngonium auritum |
| Syngonium castroi |
| Syngonium chiapense |
| Syngonium chocoanum |
| Syngonium crassifolium |
| Syngonium dodsonianum |
| Syngonium erythrophyllum |
| Syngonium foreroanum |
| Syngonium gentryanum |
| Syngonium harlingianum |
| Syngonium hastiferum |
| Syngonium hastifolium |
| Syngonium hoffmannii |
| Syngonium laterinervium |
| Syngonium llanoense |
| Syngonium macrophyllum |
| Syngonium mauroanum |
| Syngonium meridense |
| Syngonium neglectum |
| Syngonium oduberi |
| Syngonium podophyllum var. podophyllum |
| Syngonium podophyllum var. peliocladum |
| Syngonium rayi |
| Syngonium sagittatum |
| Syngonium salvadorense |
| Syngonium schottianum |
| Syngonium sparreorum |
| Syngonium standleyanum |
| Syngonium steyermarkii |
| Syngonium triphyllum |
| Syngonium wendlandii |
| Syngonium yurimaguense |
| Taccarum caudatum |
| Taccarum crassispathum |
| Taccarum peregrinum |
| Taccarum ulei |
| Taccarum warmingii |
| Taccarum weddellianum |
| Theriophonum dalzellii |
| Theriophonum danielii |
| Theriophonum fischeri |
| Theriophonum infaustum |
| Theriophonum manickamii |
| Theriophonum minutum |
| Theriophonum sivaganganum |
| Typhonium acetosella |
| Typhonium adnatum |
| Typhonium albidinervium |
| Typhonium albispathum |
| Typhonium alismifolium |
| Typhonium angustilobum |
| Typhonium bachmaense |
| Typhonium baoshanense |
| Typhonium blumei |
| Typhonium bognerianum |
| Typhonium brownii |
| Typhonium bulbiferum |
| Typhonium circinnatum |
| Typhonium cochleare |
| Typhonium conchiforme |
| Typhonium cordifolium |
| Typhonium digitatum |
| Typhonium echinulatum |
| Typhonium eliosurum |
| Typhonium filiforme |
| Typhonium flagelliforme |
| Typhonium fultum |
| Typhonium gagnepainii |
| Typhonium gallowayi |
| Typhonium glaucum |
| Typhonium griseum |
| Typhonium hayatae |
| Typhonium huense |
| Typhonium hunanense |
| Typhonium inopinatum |
| Typhonium jinpingense |
| Typhonium johnsonianum |
| Typhonium jonesii |
| Typhonium laoticum |
| Typhonium liliifolium |
| Typhonium lineare |
| Typhonium listeri |
| Typhonium medusae |
| Typhonium mirabile |
| Typhonium neogracile |
| Typhonium nudibaccatum |
| Typhonium orbifolium |
| Typhonium pedatisectum |
| Typhonium pedunculatum |
| Typhonium peltandroides |
| Typhonium penicillatum |
| Typhonium pottingeri |
| Typhonium praecox |
| Typhonium praetermissum |
| Typhonium pusillum |
| Typhonium reflexum |
| Typhonium roxburghii |
| Typhonium russell-smithii |
| Typhonium sagittariifolium |
| Typhonium saraburiensis |
| Typhonium sinhabaedyae |
| Typhonium smitinandii |
| Typhonium stigmatilobatum |
| Typhonium subglobosum |
| Typhonium taylorii |
| Typhonium trifoliatum |
| Typhonium trilobatum |
| Typhonium tubispathum |
| Typhonium varians |
| Typhonium vermiforme |
| Typhonium violifolium |
| Typhonium watanabei |
| Typhonium weipanum |
| Typhonium wilbertii |
| Typhonodorum lindleyanum |
| Ulearum donburnsii |
| Ulearum sagittatum var. sagittatum |
| Ulearum sagittatum var. viridispadix |
| Urospatha angustiloba |
| Urospatha antisylleptica |
| Urospatha caudata |
| Urospatha edwallii |
| Urospatha friedrichsthalii |
| Urospatha loefgreniana |
| Urospatha meyeri |
| Urospatha riedeliana |
| Urospatha sagittifolia |
| Urospatha somnolenta |
| Urospatha wurdackii |
| Wolffia angusta |
| Wolffia arrhiza |
| Wolffia australiana |
| Wolffia borealis |
| Wolffia brasiliensis |
| Wolffia columbiana |
| Wolffia cylindracea |
| Wolffia elongata |
| Wolffia globosa |
| Wolffia microscopica |
| Wolffia neglecta |
| Wolffiella caudata |
| Wolffiella denticulata |
| Wolffiella gladiata |
| Wolffiella hyalina |
| Wolffiella lingulata |
| Wolffiella neotropica |
| Wolffiella oblonga |
| Wolffiella repanda |
| Wolffiella rotunda |
| Wolffiella welwitschii |
| Xanthosoma acutum |
| Xanthosoma akkermansii |
| Xanthosoma aristeguietae |
| Xanthosoma auriculatum |
| Xanthosoma baguense |
| Xanthosoma bayo |
| Xanthosoma belophyllum |
| Xanthosoma bilineatum |
| Xanthosoma bolivaranum |
| Xanthosoma brasiliense |
| Xanthosoma brevispathaceum |
| Xanthosoma caladioides |
| Xanthosoma caracu |
| Xanthosoma caulotuberculatum |
| Xanthosoma conspurcatum |
| Xanthosoma contractum |
| Xanthosoma cordatum |
| Xanthosoma cordifolium |
| Xanthosoma cubense |
| Xanthosoma daguense var. daguense |
| Xanthosoma daguense var. amargalense |
| Xanthosoma dealbatum |
| Xanthosoma eggersii |
| Xanthosoma exiguum |
| Xanthosoma flavomaculatum |
| Xanthosoma fractum |
| Xanthosoma granvillei |
| Xanthosoma guttatum |
| Xanthosoma hebetatum |
| Xanthosoma helleborifolium |
| Xanthosoma herrerae |
| Xanthosoma hylaeae |
| Xanthosoma latestigmatum |
| Xanthosoma longilobum |
| Xanthosoma lucens |
| Xanthosoma mafaffoides |
| Xanthosoma mariae |
| Xanthosoma maroae |
| Xanthosoma maximiliani |
| Xanthosoma mendozae |
| Xanthosoma mexicanum |
| Xanthosoma narinoense |
| Xanthosoma nitidum |
| Xanthosoma obtusilobum |
| Xanthosoma orinocense |
| Xanthosoma paradoxum |
| Xanthosoma pariense |
| Xanthosoma peltatum |
| Xanthosoma pentaphyllum |
| Xanthosoma platylobum |
| Xanthosoma plowmanii |
| Xanthosoma poeppigii |
| Xanthosoma pottii |
| Xanthosoma puberulum |
| Xanthosoma pubescens |
| Xanthosoma pulchrum |
| Xanthosoma riedelianum |
| Xanthosoma riparium |
| Xanthosoma robustum |
| Xanthosoma sagittifolium |
| Xanthosoma saguasense |
| Xanthosoma seideliae |
| Xanthosoma stenospathum |
| Xanthosoma striatipes |
| Xanthosoma striolatum |
| Xanthosoma syngoniifolium |
| Xanthosoma taioba |
| Xanthosoma tarapotense |
| Xanthosoma trichophyllum |
| Xanthosoma trilobum |
| Xanthosoma ulei |
| Xanthosoma undipes |
| Xanthosoma viviparum |
| Xanthosoma weeksii |
| Xanthosoma wendlandii |
| Xanthosoma yucatanense |
| Zamioculcas zamiifolia |
| Zantedeschia aethiopica |
| Zantedeschia albomaculata subsp. albomaculata |
| Zantedeschia albomaculata subsp. macrocarpa |
| Zantedeschia elliottiana |
| Zantedeschia jucunda |
| Zantedeschia odorata |
| Zantedeschia pentlandii |
| Zantedeschia rehmannii |
| Zantedeschia valida |
| Zomicarpa pythonium |
| Zomicarpa steigeriana |
| Zomicarpella amazonica |
| Zomicarpella maculata |
