# Supplementary material for: A global assessment of a large monocot family highlights the need for group-specific analyses of invasiveness
Source: AoB Plants. 2016 Feb 12;8:plw009. doi: 10.1093/aobpla/plw009 (PMC4804228; doi:10.1093/aobpla/plw009)
Supplement: Additional Information [file supp_plw009_plw009supp_file2.docx]

| **Characteristics** | **Reason for selection** | **Binary code** |
| --- | --- | --- |
| Number of native regions | Species occurring over a larger range have a greater chance of becoming introduced and naturalized (proxy for range size). | Native regions ≥ 3 = 1; Native regions < 3 = 0 |
| Plant life form | Chamaephytes, geophytes, helophytes, hemicryptophytes or hydrophytes were found to be the most important life forms for Araceae to overcome the introduction and naturalization barriers. | Chamaephytes, geophytes, helophytes, hemicryptophytes or hydrophytes = 1; Other life forms = 0 |
| Pollinator type | Fly pollinated species have a higher likelihood of being introduced by humans. | Fly pollinated = 1; Other pollinators = 0 |
| Native floristic regions | Species native to the Polynesian province had a higher likelihood of being introduced. | Polynesian = 1; Non-Polynesian = 0 |
| Flower sexuality | Unisexual flowers were favoured over bisexual flowers. | Unisexual = 1; Bisexual = 0 |
| Habitat | Araceae genera are concentrated in the tropics. Species from tropical moist forests have a greater chance of being introduced. | Tropical moist forests = 1; Other habitats = 0 |
| Number of introduced regions | Species introduced into more regions have a greater chance of becoming naturalized and invasive (proxy for propagule pressure). | Introduced regions ≥ 3 = 1; Introduced regions < 3 = 0 |
| Number of uses | Species with more uses overcame the naturalization barriers. | Number of uses ≥ 3 = 1; Number of uses < 3 = 0 |
