# Supplementary figures and images for: A global assessment of a large monocot family highlights the need for group-specific analyses of invasiveness
Source: AoB Plants. 2016 Feb 12;8:plw009. doi: 10.1093/aobpla/plw009 (PMC4804228; doi:10.1093/aobpla/plw009)

(A)

**
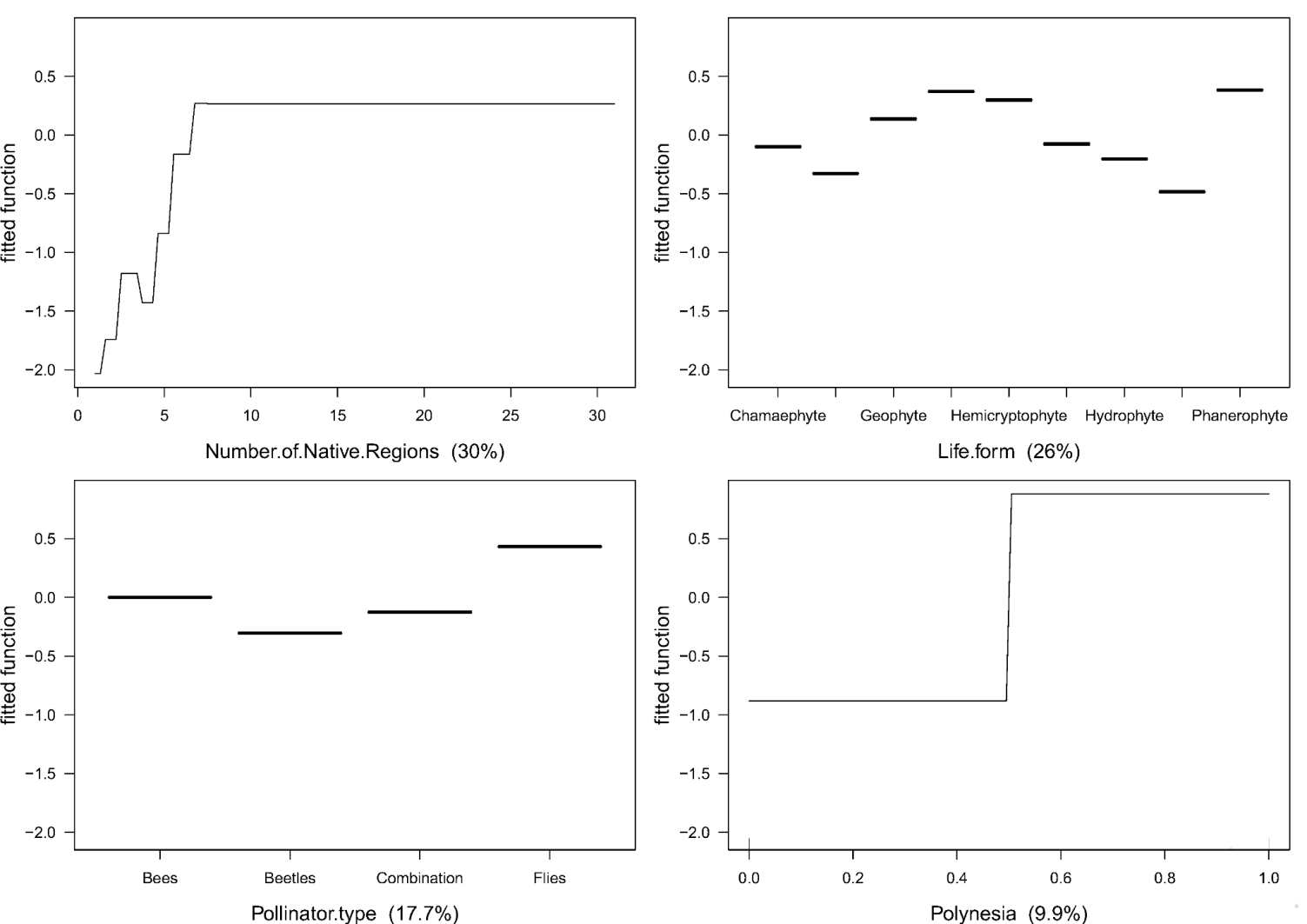
**

**
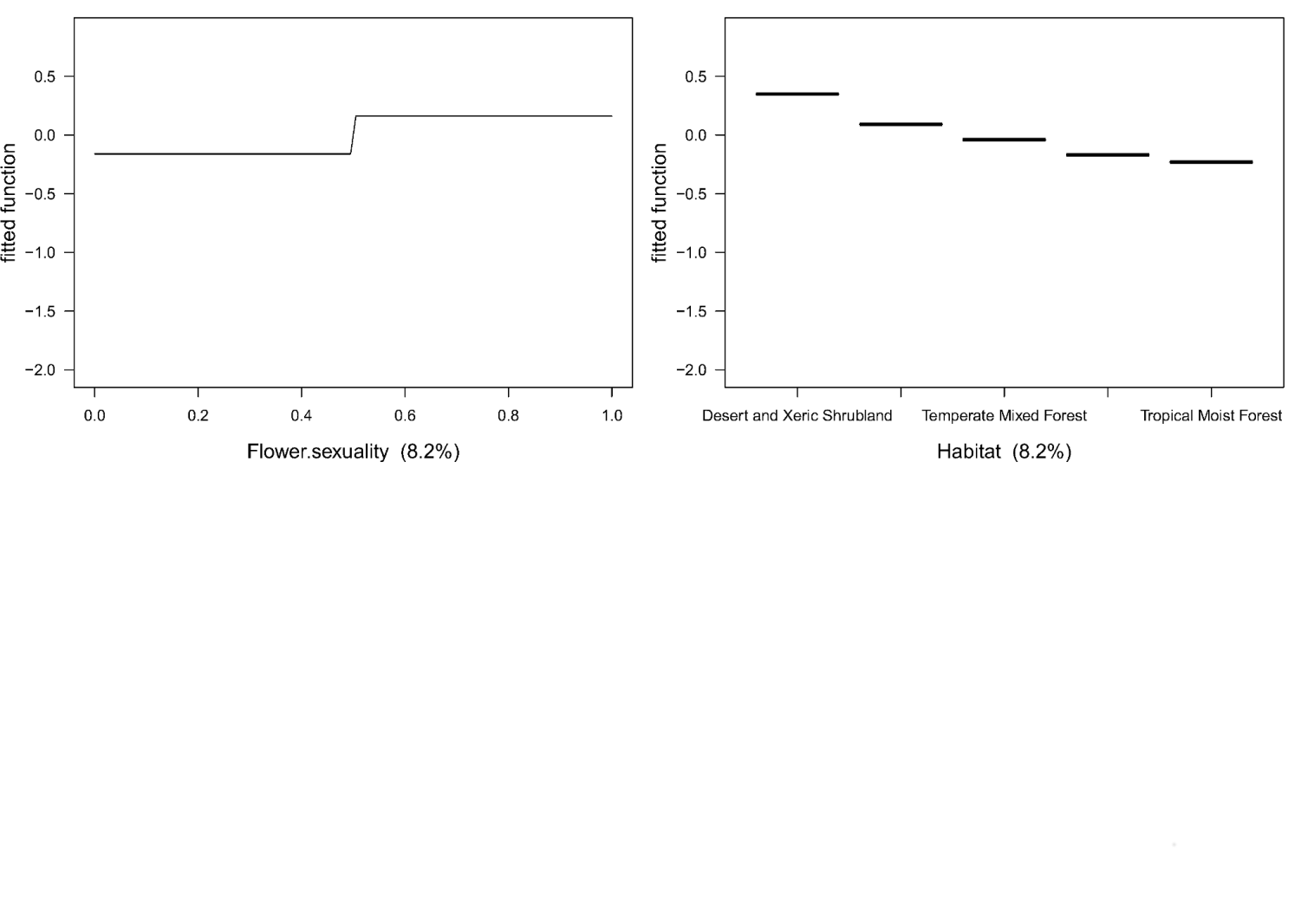
**

(B)

**
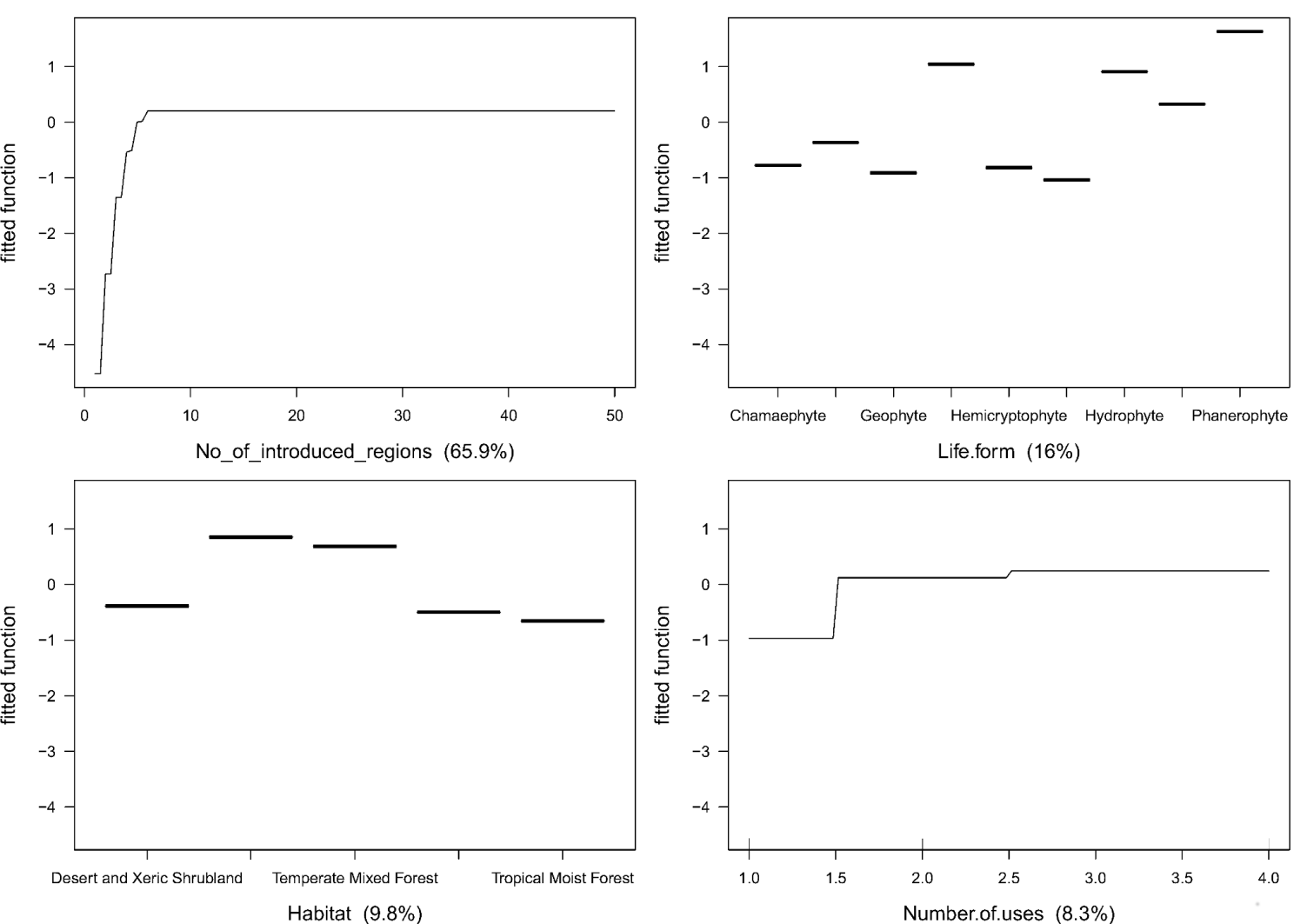
**

(C)

**
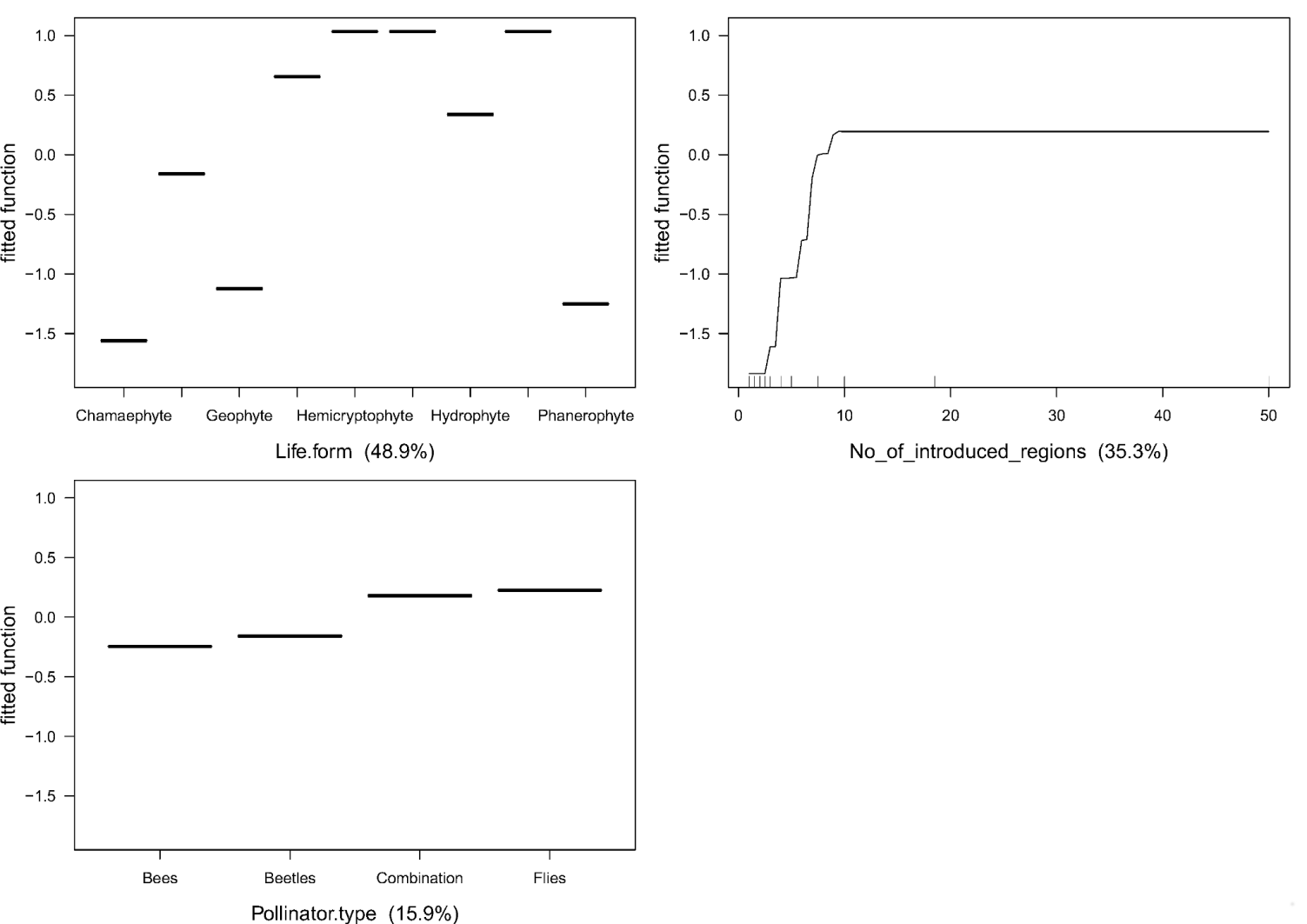
**

Supplement: Additional Information [file supp_plw009_plw009supp_file3.docx]

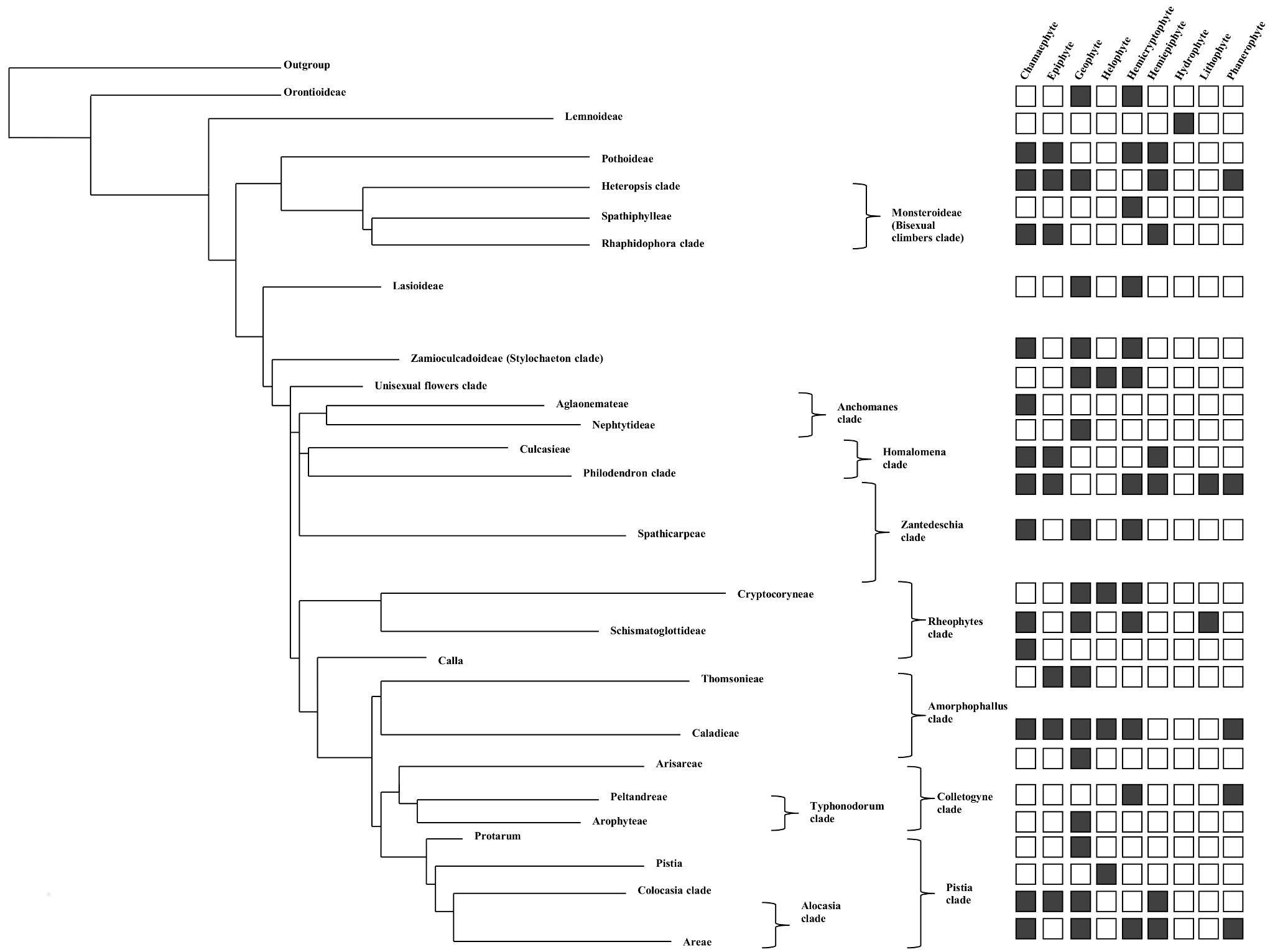

Supplement: Additional Information [file supp_plw009_plw009supp_file4.tif]

(A)


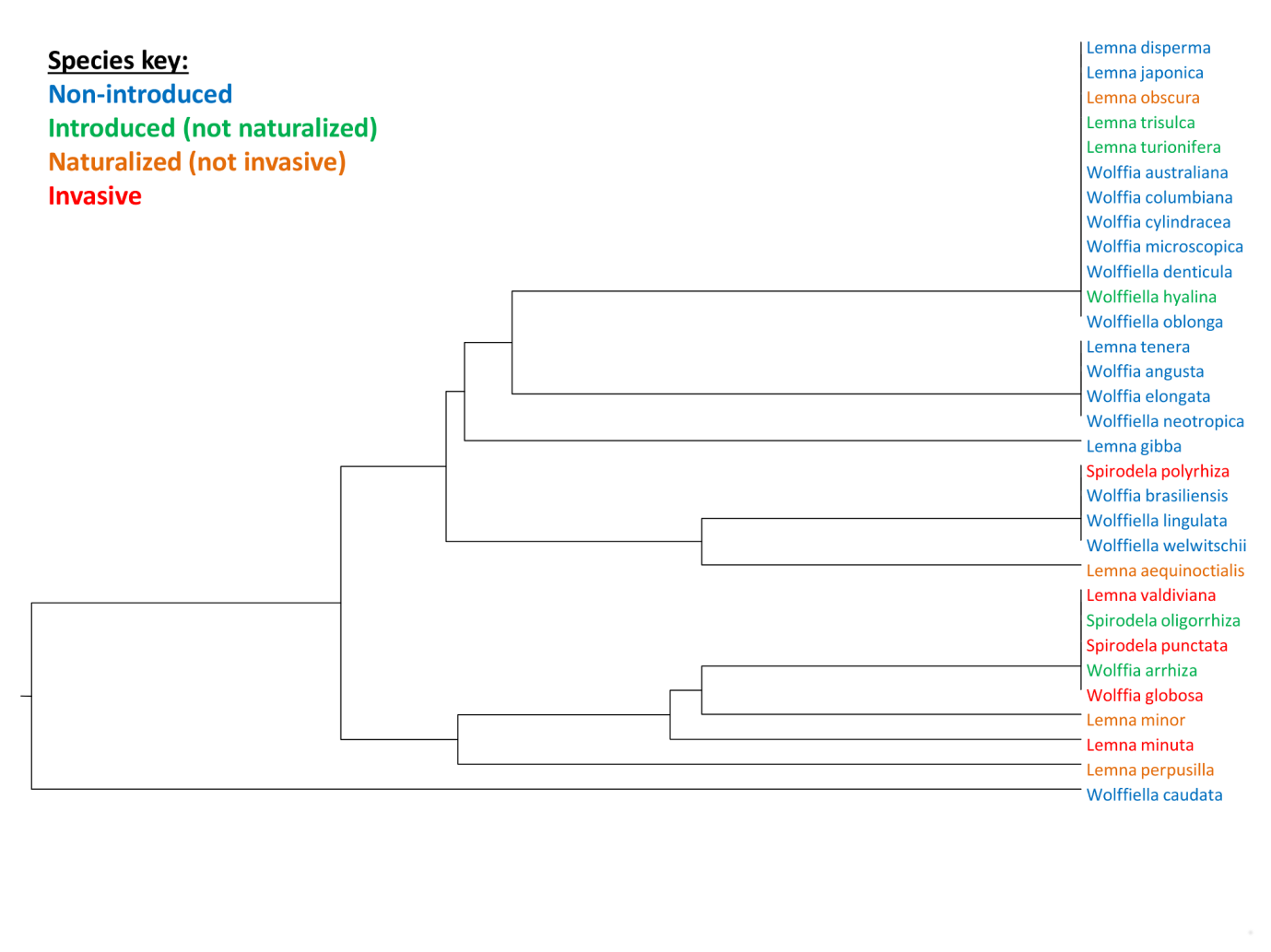


(B)


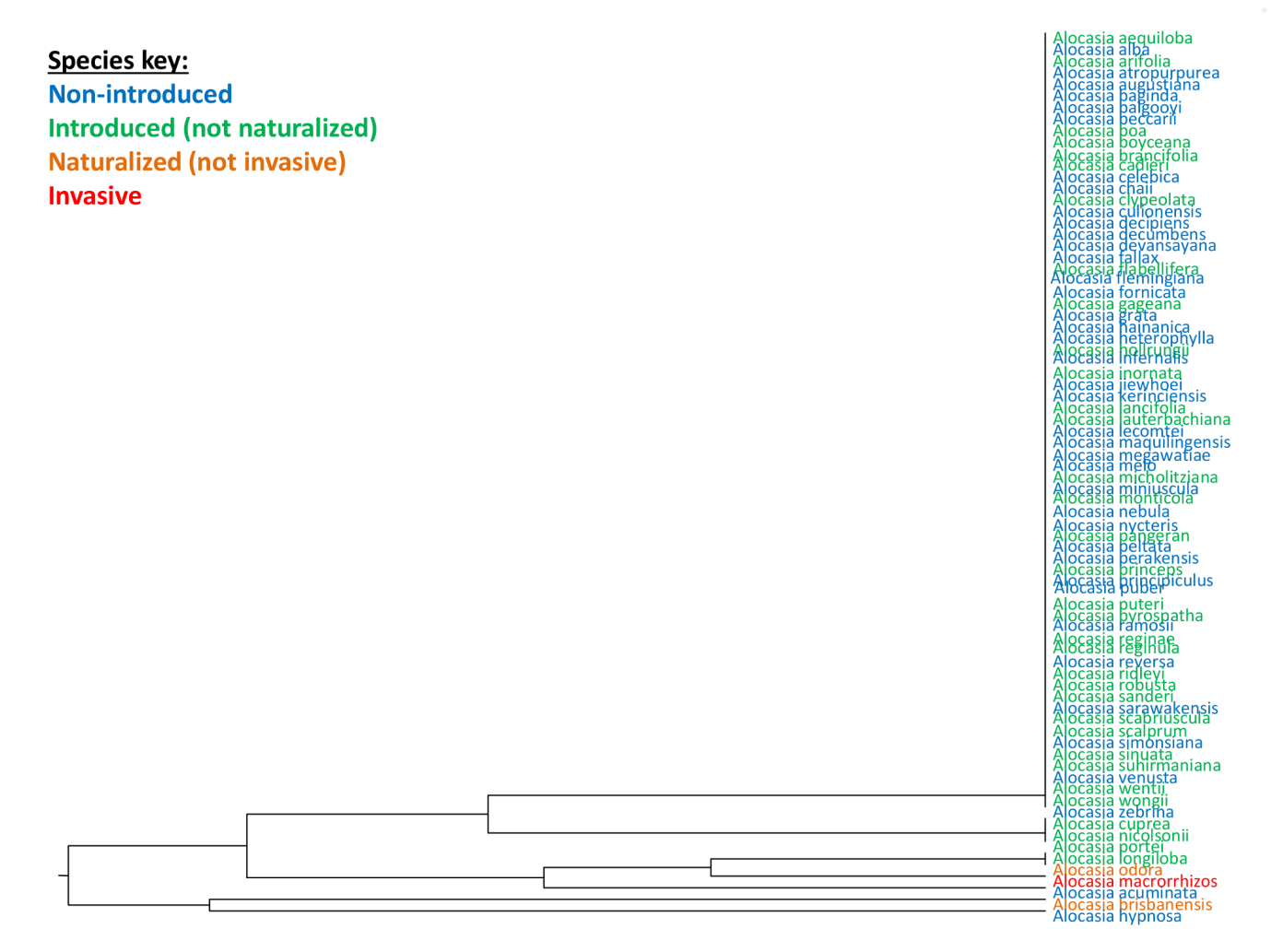


(C)


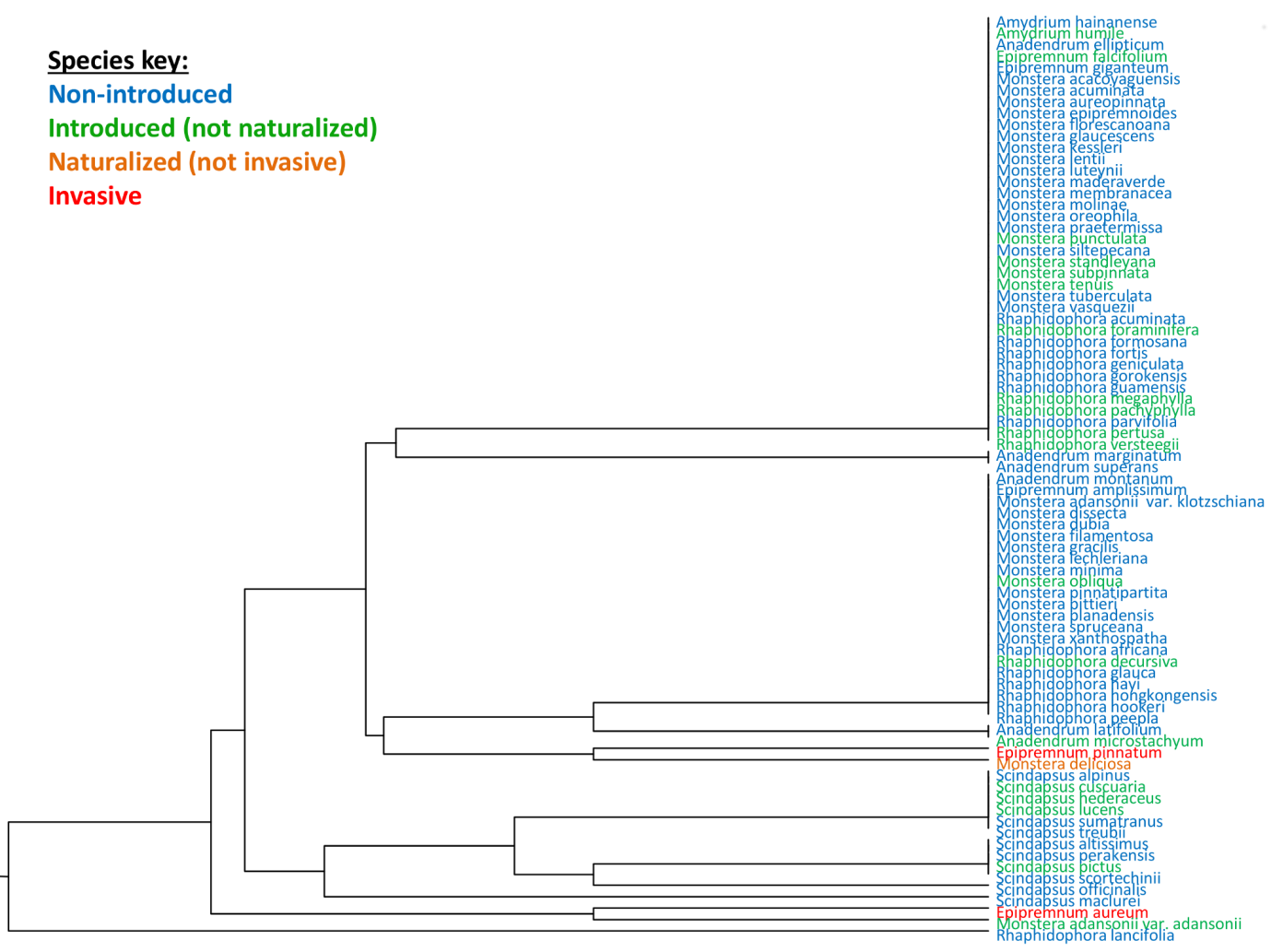


(D)


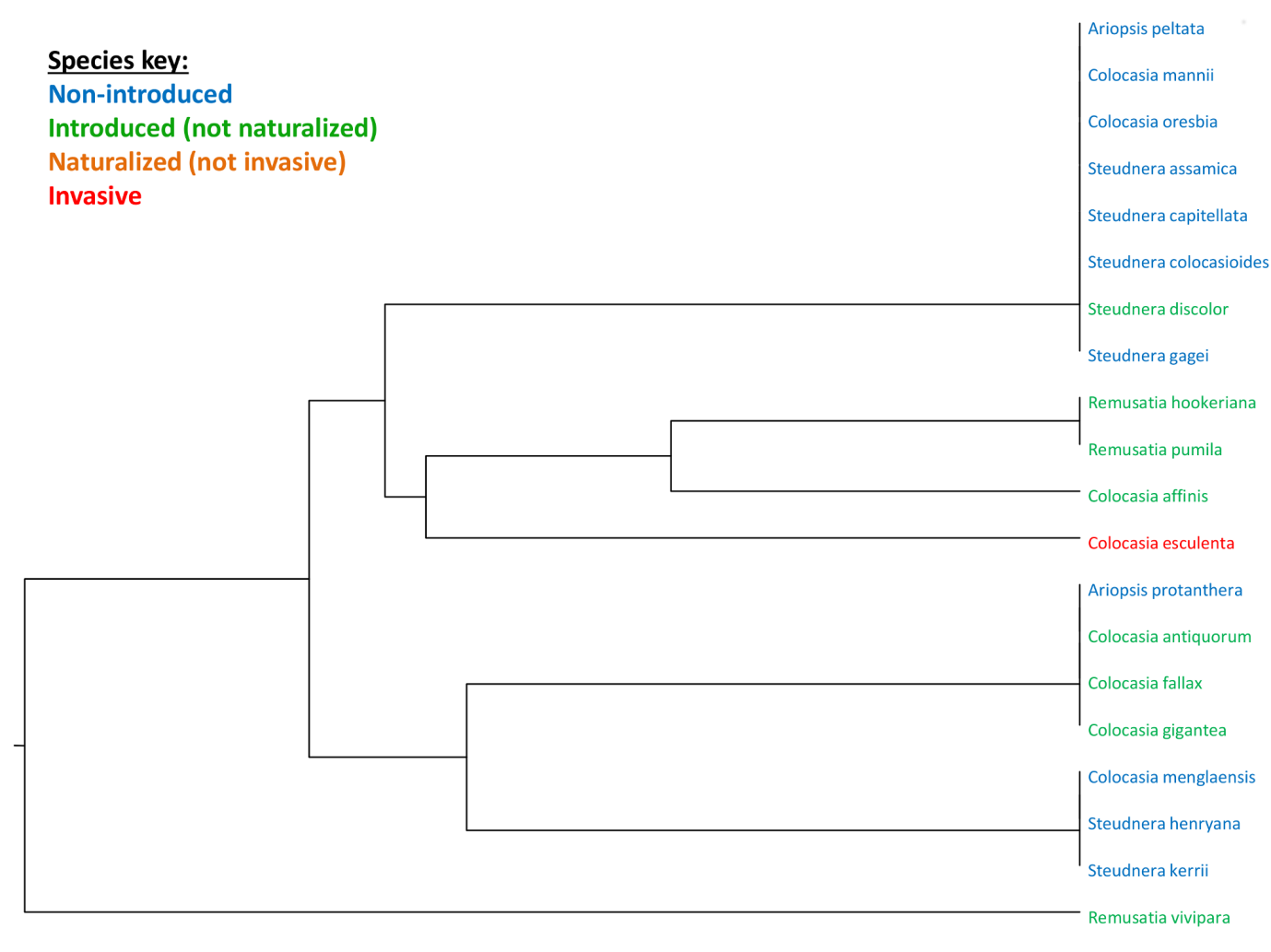


(E)


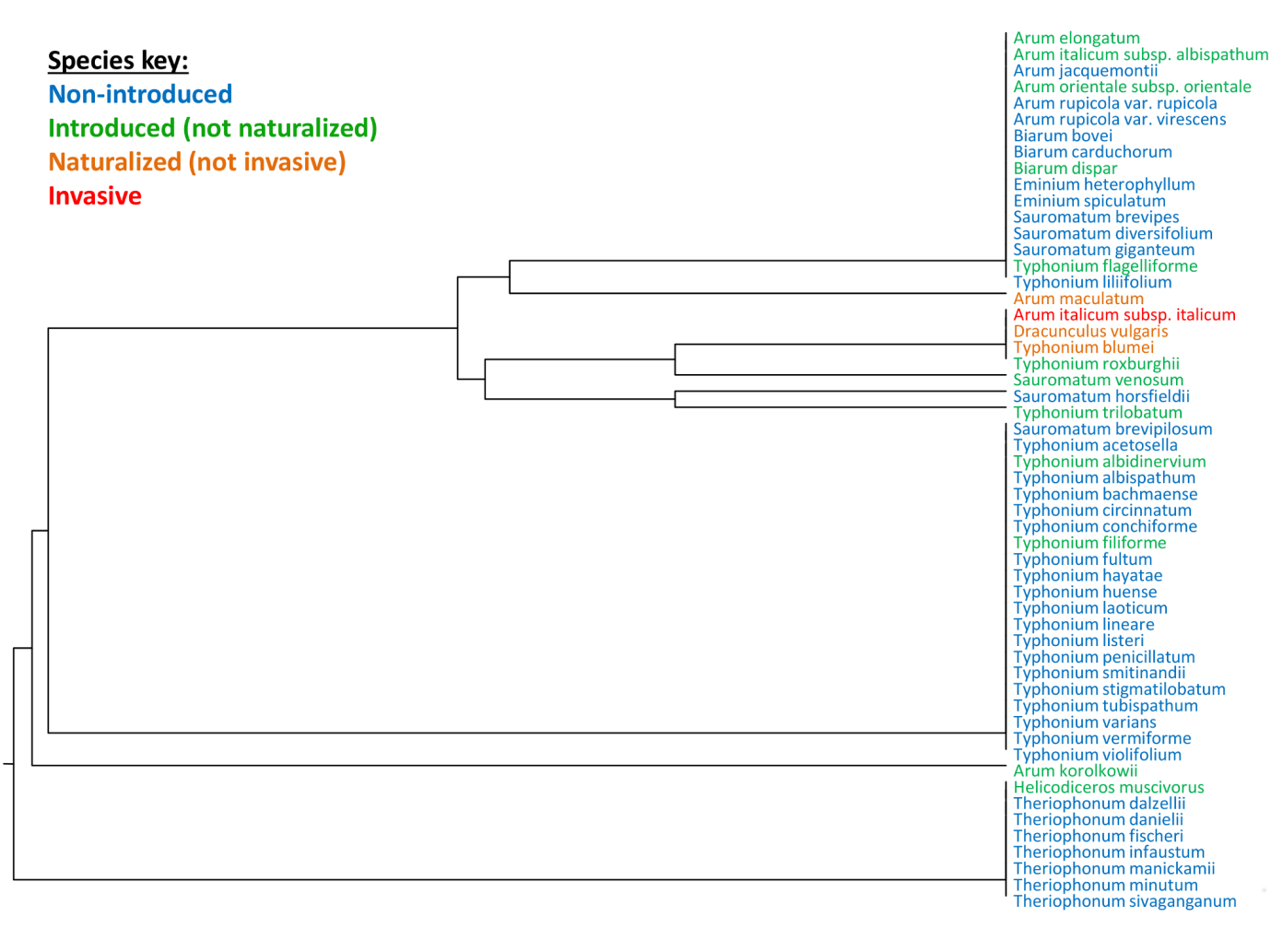


(F)


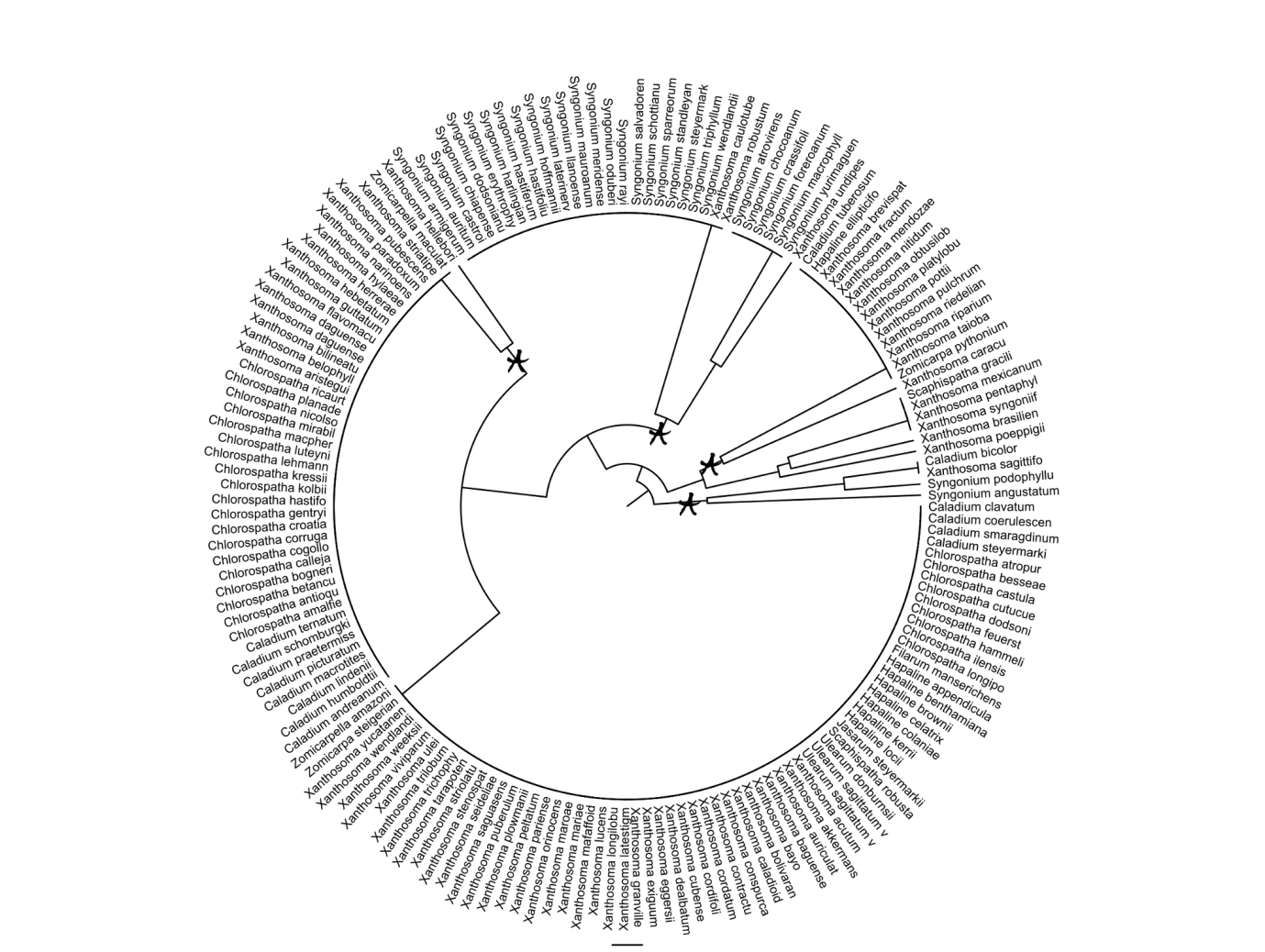


(G)


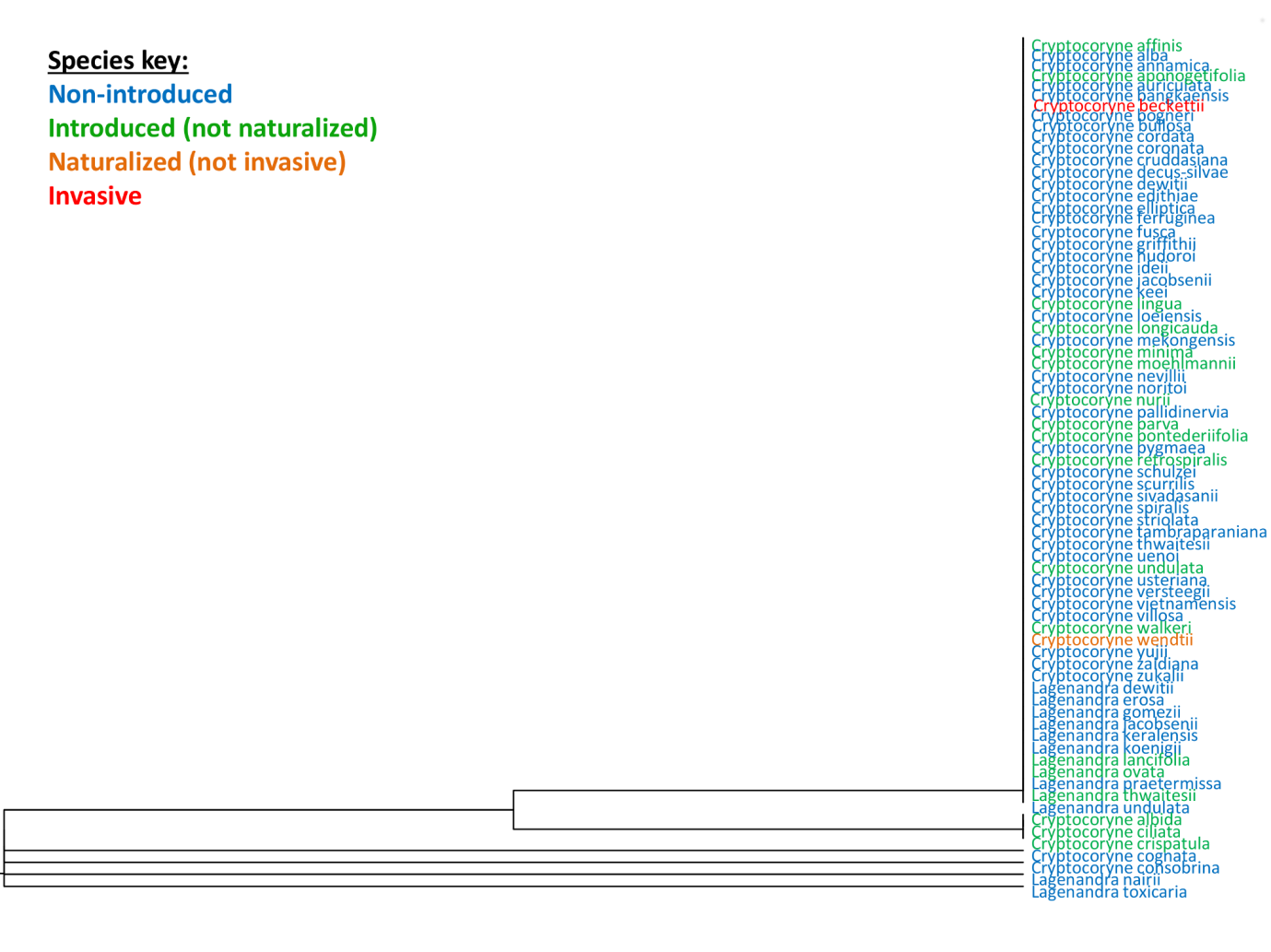


(H)


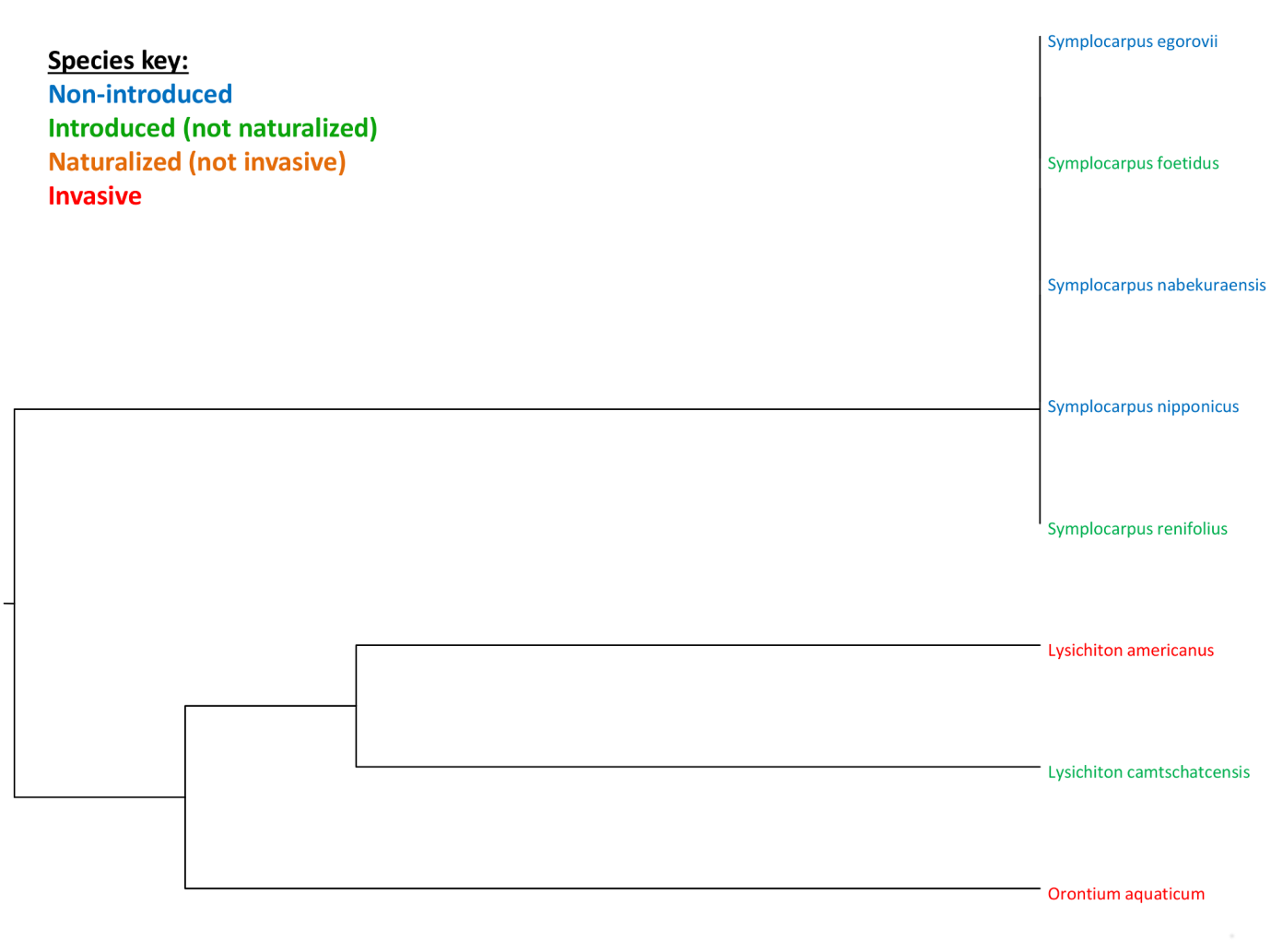

Supplement: Additional Information [file supp_plw009_plw009supp_file5.docx]
